# Scopularides Revisited: Molecular Networking Guided Exploration of Lipodepsipeptides in Australian Marine Fish Gastrointestinal Tract-Derived Fungi

**DOI:** 10.3390/md17080475

**Published:** 2019-08-16

**Authors:** Ahmed H. Elbanna, Zeinab G. Khalil, Paul V. Bernhardt, Robert J. Capon

**Affiliations:** 1 Institute for Molecular Bioscience, The University of Queensland, St Lucia, QLD 4072, Australia; 2 School of chemistry and Molecular Bioscience, The University of Queensland, St Lucia, QLD 4072, Australia

**Keywords:** *Scopulariopsis* sp., *Beauveria* sp., lipodepsipeptides, scopularides, GNPS, marine fungal biodiscovery

## Abstract

Chemical analysis of a cultivation of an Australian Mugil mullet gastrointestinal tract (GIT) derived fungus, *Scopulariopsis* sp. CMB-F458, yielded the known lipodepsipeptides scopularides A (**1**) and B (**2**). A comparative global natural product social (GNPS) molecular networking analysis of ×63 co-isolated fungi, detected two additional fungi producing new scopularides, with *Beauveria* sp. CMB-F585 yielding scopularides C–G (**3**–**7**) and *Scopulariopsis* sp. CMB-F115 yielding scopularide H (**8**). Structures inclusive of absolute configurations were assigned by detailed spectroscopic and C_3_ Marfey’s analysis, together with X-ray analyses of **3** and **8**, and biosynthetic considerations. Scopularides A–H (**1**–**8**) did not exhibit significant growth inhibitory activity against a selection of Gram positive (+ve) and negative (−ve) bacteria, a fungus, or a panel of three human carcinoma cell lines.

## 1. Introduction

**Figure 1:**
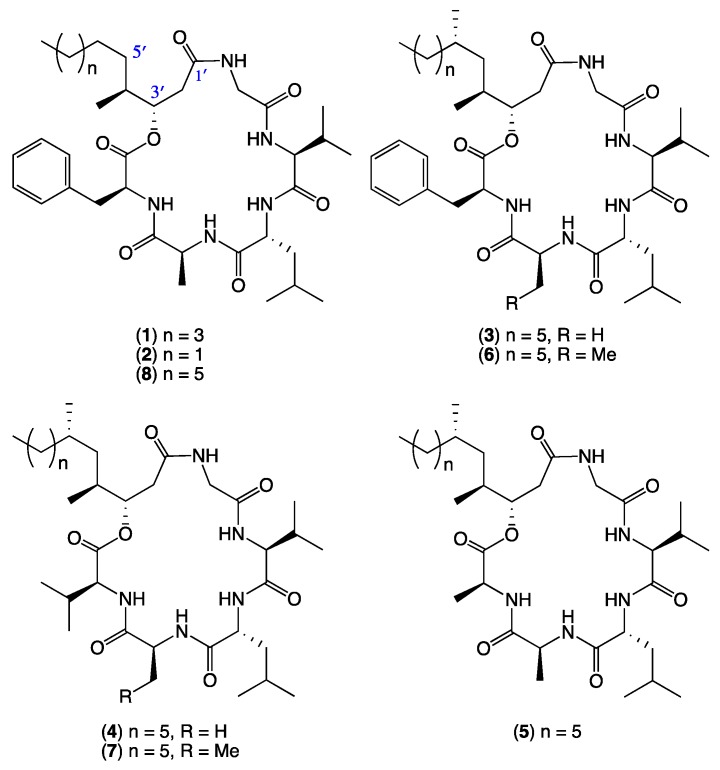
Scopularides A–H (**1**–**8**) from Mugil mullet gastrointestinal tract (GIT)-derived fungi.

As part of our ongoing investigation into secondary metabolites from Australian marine-derived fungi, we speculated that selected bottom feeding fish species may act as natural myco-accumulators, and as such could be a readily accessible source of marine-derived fungi. Using three Mugil mullet acquired from a local fish market, we assembled a library of ~500 chemically-distinct gastrointestinal tract (GIT)-derived fungi. In a preliminary validation of the potential of this resource, we reported on an unprecedented class of hydrazine containing furano Schiff bases, the prolinimines, from *Trichoderma* sp. CMB-F563 [1]. Building on this achievement, we now describe an investigation into scopularide lipodepsipeptides, literature accounts of which are limited to scopularides A–B (**1**–**2**) from the marine sponge-derived fungus *Scopulariopsis brevicaulis* NCPF-2177 [2]. This current study was prompted by a fortuitous re-isolation of scopularides A–B (**1**–**2**) from the Mugil mullet GIT-derived *Scopulariopsis* sp. CMB-F458. Using **1** and **2** as authentic standards, a comparative global natural product social (GNPS) molecular networking [3] analysis of extracts obtained from ×63 co-isolated GIT-derived fungi, enabled the detection, and subsequent scaled-up cultivation, isolation and identification of the new scopularides C–G (**3**–**7**) from *Beauveria* sp. CMB-F585, and the new scopularide H (**8**) from *Scopulariopsis* sp. CMB-F115. Structures were assigned to **3**–**8** (Figure 1) on the basis of detailed spectroscopic and chemical analysis, as summarised below.

## 2. Results and Discussion

**Figure 2:**
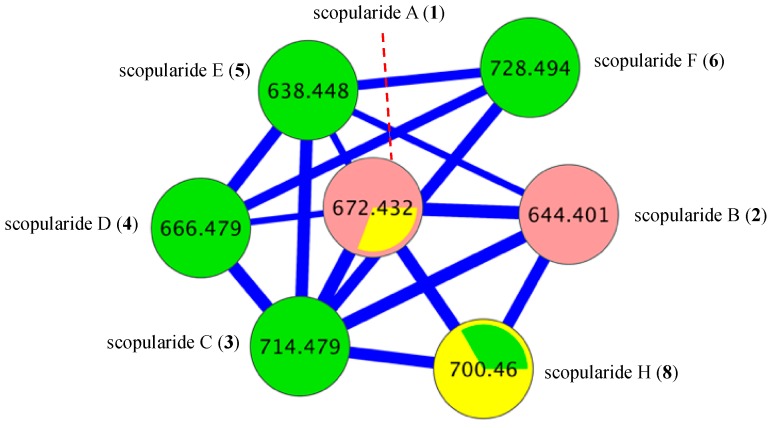
Global natural product social (GNPS) molecular networking cluster for scopularides detected in a comparative analysis of ×63 Mugil mullet GIT-derived fungal extracts. CMB-F458 pink nodes, CMB-F585 green nodes, and CMB-F115 yellow nodes.

The EtOAc extract from a M1S solid phase (20 × agar plate) cultivation of the Mugil mullet GIT-derived fungus *Scopulariopsis* sp. CMB-F458 was subjected to sequential solvent partitioning followed by reversed-phase HPLC to yield scopularides A–B (**1**–**2**). Planar structures for **1**–**2** were independently assigned by detailed spectroscopic analysis, with amino acid residues inclusive of absolute configurations assigned by C_3_ Marfey’s analysis (Figures S46 and S47) [4]. The complete structures for **1**–**2** were confirmed by 1D NMR (methanol-*d*_4_) in comparison to literature data (Tables S1 and S3) [2]. Armed with authentic standards of **1** and **2**, a comparative GNPS analysis of extracts obtained from cultivations of ×63 co-isolated fungi detected a “scopularide” cluster incorporating nodes from *Scopulariopsis* sp. CMB-F458 and two other fungal strains, *Beauveria* sp. CMB-F585 and *Scopulariopsis* sp. CMB-F115 (Figure 2).

**Figure 3:**
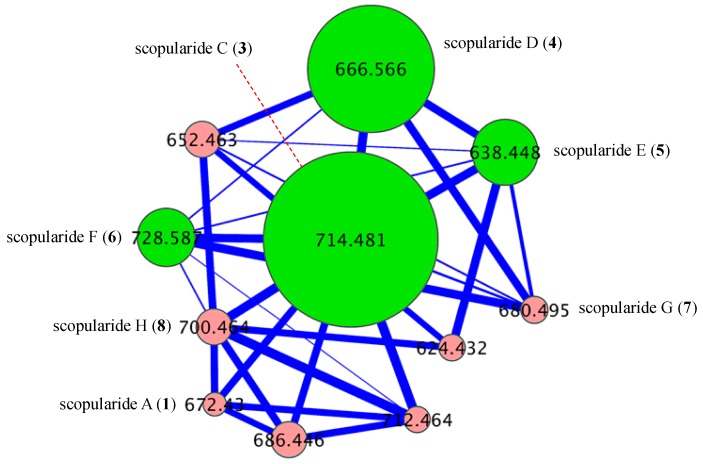
GNPS cluster for scopularides detected in a YES solid phase cultivation of *Beauveria* sp. CMB-F585. Green nodes are scopularides C–F (**3**–**6**), and pink nodes are minor analogues.

**Figure 4:**
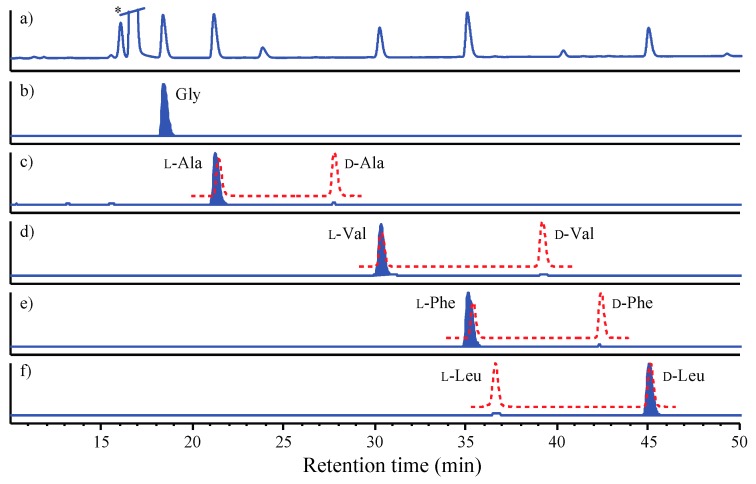
C_3_ Marfey’s analysis of scopularide C (**3**). (a) C_3_ HPLC-DAD (340 nm) chromatogram of l-FDAA amino acid derivatives of acid hydrolysate of an aliquot (50 µg) of **3**. (b–f) HPLC-MS single ion extraction (SIE) chromatograms for l-FDAA derivatives of authentic standards (red broken lines) and the acid hydrolysate of **3** (blue shaded peaks). (b) Gly (SIE *m*/*z* 328), (c) l-Ala (SIE *m*/*z* 342), (d) l-Val (SIE *m*/*z* 370), (e) l-Phe (SIE *m*/*z* 418) and (f) d-Leu (SIE *m*/*z* 384). * Excess Marfey’s reagent.

**Figure 5:**
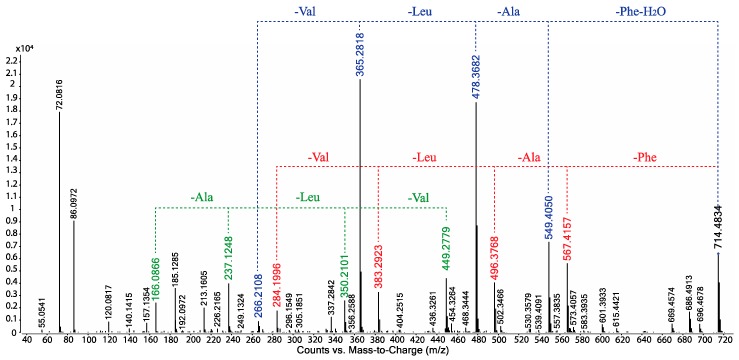
UPLC-QTOF-MS/MS fragmentations for scopularide C (**3**).

**Figure 6:**
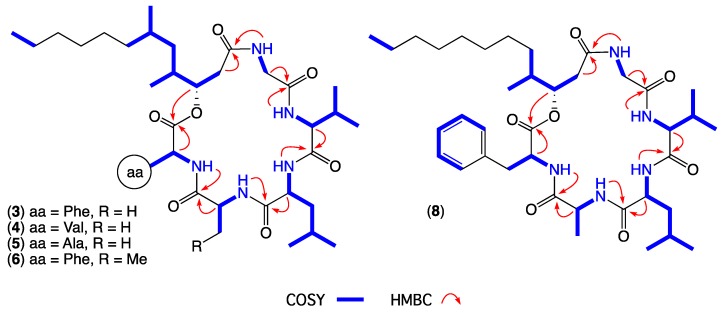
Diagnostic 2D NMR (DMSO-*d*_6_) correlations for scopularides C–F (**3**–**6**) and H (**8**).

**Figure 7:**
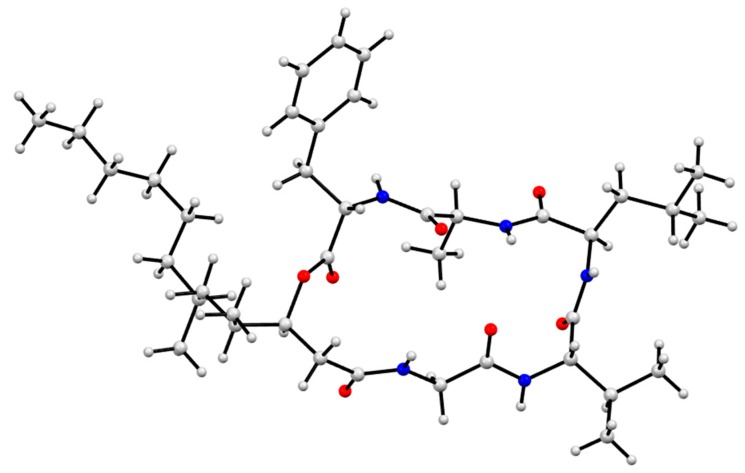
X-ray crystal structure of scopularide C (**3**).

GNPS analysis of the EtOAc extract obtained from a YES solid phase (80 × agar plate) cultivation of *Beauveria* sp. CMB-F585 detected the presence of four prominent new scopularides C–F (**3**–**6**), together with trace amounts of the new scopularides G-H (**7**–**8**) and known scopularide A (**1**) (Figure 3). Solvent extraction and partitioning of the EtOAc extract, followed by reversed-phase HPLC, yielded five new scopularides **3**–**7**. HRESI(+)MS analysis of scopularide C (**3**) revealed a molecular formula (C_39_H_63_N_5_O_7_) consistent with a higher homologue (+C_3_H_6_) of scopularide A (**1**). A C_3_ Marfey’s analysis (Figure 4) supported by diagnostic of MS/MS fragmentations (Figure 5) and 1D and 2D NMR (DMSO-*d*_6_) (Table 1 and Table S5, Figure 6 and Figures S14–S18) established the amino acid sequence l-Phe^1^-L-Ala^2^-d-Leu^3^-l-Val^4^-Gly^5^. The complete structure for scopularide C (**3**), including absolute configuration about the 3*S*,4*S*,6*S*-3-hydroxyl-4,6-dimethyllauric acid (HDMLA) lipid residue, was confirmed by single crystal X-ray analysis (Figure 7 and Figure S55).

HRESI(+)MS analysis of scopularide D (**4**) revealed a molecular formula (C_35_H_63_N_5_O_7_) consistent with an l-Phe to l-Val substitution analogue of **3**, with C_3_ Marfey’s analysis (Figure S49) supported by diagnostic of MS/MS fragmentations (Figure S40) and 1D and 2D NMR (DMSO-*d*_6_) data (Table 1 and Table S6, Figure 6 and Figures S19–S23) establishing the amino acid sequence l-Val^1^-l-Ala^2^-d-Leu^3^-l-Val^4^-Gly^5^. Similarly, HRESI(+)MS analysis of scopularide E (**5**) revealed a molecular formula (C_33_H_59_N_5_O_7_) consistent with an l-Phe to l-Ala substitution analogue of **3**, with C_3_ Marfey’s analysis (Figure S50) supported by diagnostic of MS/MS fragmentations (Figure S41) and 1D and 2D NMR (DMSO-*d*_6_) data (Table 1 and Table S7, Figure 6 and Figures S24–S28) establishing the amino acid sequence l-Ala^1^-l-Ala^2^-d-Leu^3^-l-Val^4^-Gly^5^. Likewise, HRESI(+)MS analysis of scopularide F (**6**) revealed a molecular formula (C_40_H_65_N_5_O_7_) consistent with a homologue (+CH_2_) of **3**, with C_3_ Marfey’s analysis (Figure S51) supported by diagnostic of MS/MS fragmentations (Figure S42) and 1D and 2D NMR (DMSO-*d*_6_) data (Table 2 and Table S8, Figure 6 and Figures S29–S33) establishing the amino acid sequence l-Phe^1^-l-Abu^2^-d-Leu^3^-l-Val^4^-Gly^5^ (where l-Abu is l-alpha-aminobutyric acid). Importantly, the structure and absolute configuration of the lipid residue in **4**–**6** were assigned on the basis of spectroscopic comparisons with **3**, and biosynthetic considerations.

Although scopularide G (**7**) was only isolated at yields that precluded the acquisition of 1D and 2D NMR data, HRESI(+)MS analysis revealed a molecular formula (C_36_H_65_N_5_O_7_) consistent with an l-Phe to l-Val substitution analogue of **6**, with C_3_ Marfey’s analysis (Figure S52) supported by diagnostic of MS/MS fragmentations (Figures S43 and S44) establishing the amino acid sequence l-Val^1^-l-Abu^2^-d-Leu^3^-l-Val^4^-Gly^5^. Based on biosynthetic considerations, the structure and absolute configuration of the lipid residue in **7** was proposed to be in common with **3**. Although the putative scopularide H (**8**) could be detected in the GNPS analysis of *Beauveria* sp. CMB-F585 (Figure 2 and Figure 3), its isolation and structure elucidation were more readily achieved from *Scopulariopsis* sp. CMB-F115 (Figure 2).

**Figure 8:**
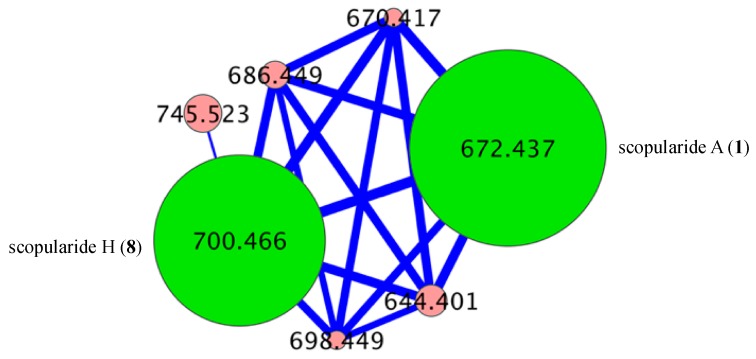
GNPS cluster for scopularides detected in a YES solid phase cultivation of *Scopulariopsis* sp. CMB-F115. Green nodes are scopularides A (**1**) and H (**8**), and pink nodes are minor analogues.

**Figure 9:**
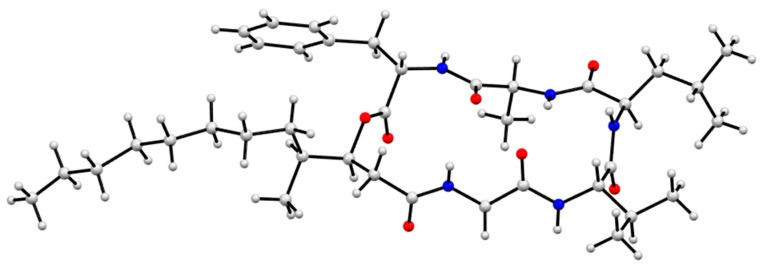
X-ray crystal structure of scopularide H (**8**).

GNPS analysis of the EtOAc extract obtained from a YES solid phase (40 × agar plate) cultivation of *Scopulariopsis sp.* CMB-F115 detected the presence of the prominent new scopularide H (**8**) and the known scopularide A (**1**) (Figure 8). Solvent extraction and partitioning of the EtOAc extract, followed by reversed-phase HPLC, yielded the known scopularide A (**1**) together with the new analogue **8**. HRESI(+)MS analysis of scopularide H (**8**) revealed a molecular formula (C_38_H_61_N_5_O_7_) consistent with a lower homologue (−CH_2_) of **3**. A diagnostic of MS/MS fragmentations (Figure S45) and 1D and 2D NMR (DMSO-*d*_6_) (Table 2 and Table S9, Figure 6 and Figures S34–38) established the amino acid sequence Phe^1^-Ala^2^-Leu^3^-Val^4^-Gly^5^. Finally, the complete structure for scopularide H (**8**), including absolute configuration about the amino acids and 3*S*,4*S*-3-hydroxyl-4-methyllauric acid (3*S*,4*S*- HMLA) lipid residue, was confirmed by single crystal X-ray analysis (Figure 9 and Figure S56).

The scopularides A–H (**1**–**8**) did not exhibit any significant growth inhibition (IC_50_ > 30 μM) when tested against drug sensitive Gram −ve *Escherichia coli* ATCC 11775 and Gram +ve *Staphylococcus aureus* ATCC 9144, or clinical isolates of both methicillin-susceptible and methicillin-resistant *Staphylococcus aureus*, and a clinical isolate of vancomycin-resistant *Enterococcus faecalis*. Likewise, **1**–**8** were not cytotoxic towards the fungus *Candida albicans* ATCC 10231 or human colorectal (SW620), lung (NCI-H460) or hepatocellular (HepG2) carcinoma cells.

Literature accounts of scopularides and related lipodepsipeptides are limited to scopularides A–B (**1**–**2**) from the marine sponge-derived fungus *Scopulariopsis brevicaulis* NCPF-2177 [2]; oryzamides A–E from the marine sponge-derived fungus *Nigrospora oryzae* PF18 [5]; chrysogeamides A–G from the marine coral-derived *Penicillium chrysogenum* CHNSCLM-0003 [6]; emericellamides A–B from the marine green alga-derived fungus *Emericella* sp. CNL-878 co-cultured with the marine sediment-derived actinomycete *Salinispora arenicola* CNH-665 [7]; emericellamides C–F from the non-marine *Aspergillus nidulans* [8]; nodupetide from the insect-derived fungus *Nodulisporum* sp. IFB-A163 [9]; as well as numerous isariins and iso-isariins from *Isaria* spp. and *Beauveria felina* [10,11,12,13,14,15,16,17]. Somewhat surprisingly, they also include arenamides A–C from the marine sediment-derived actinomycete *Salinispora arenicola* CNT-088 [18]. A summary of the amino acid and lipid residue compositions of these metabolites is presented in Table 3 and Table 4.

Notwithstanding the high level of homology shown across Table 3 and Table 4, in addition to comparing the 1D NMR (methanol-*d*_4_) data for our re-isolated samples of **1** and **2** with literature data (Tables S1 and S3) [2], we were surprised to find an equally good match of the 1D NMR (DMSO-*d*_6_) data for **1** and **2** with that reported for arenamides A–B (Tables S2–S4) [18,20]. Whether this latter observation is coincidence, or indicative of a common D-Leu^3^ configuration, remains unclear.

## 3. Materials and Methods

### 3.1. General Experimental Procedures

Chemicals were purchased from Sigma-Aldrich or Merck unless otherwise specified. Analytical-grade solvents were used for solvent extractions. Solvents used for Sephadex chromatography, HPLC, UPLC, and HPLC-MS purposes were of HPLC grade supplied by Labscan or Sigma-Aldrich and filtered/degassed through 0.45 μm polytetrafluoroethylene (PTFE) membrane prior to use. Deuterated solvents were purchased from Cambridge Isotopes (Tewksbury, MA, USA).

Microorganisms were manipulated under sterile conditions using a Laftech class II biological safety cabinet and incubated in either MMM Friocell incubators (Lomb Scientific, NSW, Australia) or an Innova 42R incubator shaker (John Morris, NSW, Australia) with temperature set at 26.5 °C. Applikon micro-bioreactors were purchased from Enztech Pty Ltd. (Woollahra, NSW, Australia).

Liquid chromatography-diode array-mass spectrometry (HPLC-DAD-MS) data were acquired on an Agilent 1100 series separation module equipped with an Agilent 1100 series HPLC/MSD mass detector and diode array multiple wavelength detector. Semi-preparative and preparative HPLCs were performed using Agilent 1100 series HPLC instruments with corresponding detectors, fraction collectors and software inclusively. UPLC chromatograms were obtained on Agilent 1290 infinity UPLC system equipped with diode array multiple wavelength detector (Zorbax C8 RRHD 1.8 μm column, 50 × 2.1 mm, eluting with 0.417 mL/min 90% H_2_O/MeCN to 100% MeCN (with isocratic 0.01% TFA modifier) over 2.50 min). Ultra-high-performance-liquid-chromatography quadrupole-time-of-flight-mass-spectrometry (UHPLC-QTOF) analysis was performed on a UHPLC-QTOF instrument comprising an Agilent 1290 Infinity II UHPLC (Zorbax C_8_ RRHD 1.8 μm column, 50 × 2.1 mm, eluting with 0.5 mL/min of isocratic 90% H_2_O/MeCN for 0.5 min followed by gradient elution to 100% MeCN over 4.5 min (with isocratic 0.1% formic acid modifier) coupled to an Agilent 6545 Q-TOF. MS/MS analysis was performed on the same instrument for ions detected in the full scan at an intensity above 1000 counts at 10 scans/s, with an isolation width of 4 ~*m*/*z* using a fixed collision energy and a maximum of 3 selected precursors per cycle.

Chiroptical measurements ([α]D) were obtained on a JASCO P-1010 polarimeter in a 100 × 2 mm cell at specified temperatures. Nuclear magnetic resonance (NMR) spectra were acquired on a Bruker Avance 600 MHz spectrometer with either a 5 mm PASEL 1H/D-13C Z-Gradient probe or 5 mm CPTCI 1H/19F-13C/15N/DZ-Gradient cryoprobe, controlled by TopSpin 2.1 software. In all cases spectra were acquired at 25 °C in DMSO with referencing to residual ^1^H or ^13^C signals (δ_H_ 2.50 and δ_C_ 39.51 ppm) in the deuterated solvent, and in MeOH with referencing to residual ^1^H or ^13^C signals (δ_H_ 3.31 and δ_C_ 49.15 ppm) in the deuterated solvent. Electrospray ionization mass spectrometry (ESIMS) experiments were carried out on an Agilent 1100 series LC/MSD (quadrupole) instrument in both positive and negative modes. High-resolution ESIMS spectra were obtained on a Bruker micrOTOF mass spectrometer by direct injection in MeOH at 3 μL/min using sodium formate clusters as an internal calibrant.

### 3.2. Fungal Strain Isolation and Taxonomy

The fungal isolates CMB-F458, CMB-F585 and CMB-F115 were isolated from the gastrointestinal tract of a specimen of Mugil mullet fish, on M1 agar plates in presence of 3.3% artificial sea salt (M1S) incubated at 26.5 °C for 8 days. Genomic DNA for the three fungi were extracted from their corresponding mycelia using the DNeasy Plant Mini Kit (Qiagen) as per the manufacturers protocol. The 18s rRNA genes were amplified by PCR using the universal internal transcribed spacer primers ITS-1 (5′-TCCGTAGGTGAACCTGCGG-3′) and ITS-4 (5′-TCCTCCGCTTATTGATATGC-3′) purchased from Sigma-Aldrich. The PCR mixture (50 μL) contained 1 μL of genomic DNA (20–40 ng), 200 μM of each deoxynucleoside triphosphate (dNTP), 1.5 mM MgCl_2_, 0.3 μM of each primer, 1 U of *Taq* DNA polymerase (Fisher Biotec) and 5 μL of PCR buffer. PCR was performed using the following conditions: initial denaturation at 95 °C for 3 min, 30 cycles in series of 94 °C for 30 s (denaturation), 55 °C for 60 s (annealing) and 72 °C for 60 s (extension), followed by one cycle at 72 °C for 6 min. PCR products were purified with PCR purification kit (Qiagen, Victoria, Australia). Amplification products were examined by agarose gel electrophoresis. The DNA sequencing was performed by the Australian Genome Research Facility (AGRF) at the University of Queensland. BLAST analyses (NCBI database) on the ITS gene sequence for (a) CMB-F458 (GenBank accession no. MN080404) revealed 99% identity with the fungal strain *Scopulariopsis* sp.; (b) CMB-F585 (GenBank accession no. MN080403) revealed 98% identity with the fungal strain *Beauveria* sp.; and (c) CMB-F115 (GenBank accession no. MN080405) revealed 98% identity with the fungal strain *Scopulariopsis* sp.

### 3.3. Scale up Cultivation of Scopulariopsis sp. CMB-F458

A single colony of CMB-F458 was used to inoculate M1S agar plates (×20), which were incubated at 26.5 °C for 8 days, diced, extracted with EtOAc (400 mL), filtered and concentrated in vacuo at 40 °C to yield a crude extract (80 mg). The crude extract was triturated (defatted) with *n*-hexane (2 × 10 mL), after which the insolubles (35 mg) were subjected to preparative reversed-phase HPLC (Phenomenex Luna-C_18_ 21.2 mm × 25 cm × 10 µm column, 20 mL/min gradient elution over 20 min from 90% H_2_O/MeCN to 100% MeCN with a constant isocratic 0.01% TFA/MeCN) to yield scopularides A (**1**) (5 mg, 6.25%) and B (**2**) (1.3 mg, 1.62%) (Scheme S1).

### 3.4. Scale-Up Cultivation of Beauveria sp. CMB-F585

A single colony of CMB-F585 was used to inoculate YES agar plates (×80), which were incubated at 26.5 °C for 8 days, after which the agar was diced, extracted with EtOAc (2 × 500 mL), filtered and concentrated in vacuo at 40 °C to yield a crude extract (1.3 g). The crude extract was sequentially triturated with *n*-hexane (3 × 50 mL), DCM (3 × 50 mL) and MeOH (3 × 50 mL) to afford, after concentration in vacuo, *n*-hexane (400 mg), DCM (800 mg) and MeOH (130 mg) solubles. A portion of the DCM solubles (700 mg) was subjected to gel chromatography (Sephadex^®^ LH-20 2.5 × 70 cm column, gravity elution with isocratic MeOH). Fractions (10 mL) were monitored by UPLC-QTOF analysis to reveal fraction -2-1 (200 mg) as rich in target lipodepsipeptides, after which an aliquot (60 mg) was subjected to semi-preparative reversed-phase HPLC (Zorbax Eclipse XDB-C_8_ 9.4 mm × 25 cm × 5 µm, 3 mL/min isocratic elution over 40 min with 35% H_2_O/MeCN and 0.1% TFA/MeCN) to yield scopularides C (**3**) (11.2 mg, 2.8%), D (**4**) (5.5 mg, 1.4%), E (**5**) (2.5 mg, 0.64%), F (**6**) (1.7 mg, 0.43%) and G (**7**) (0.3 mg, 0.07%) (Scheme S2).

### 3.5. Scale-Up Cultivation of Scopulariopsis sp. CMB-F115

A single colony of CMB-F115 was used to inoculate YES agar plates (×40) which were incubated at 26.5 °C for 8 days, diced, extracted with EtOAc (400 mL × 2), filtered and concentrated in vacuo at 40 °C, to yield a crude extract (300 mg). The crude extract was sequentially triturated with *n*-hexane (2 × 50 mL), DCM (2 × 50 mL) and MeOH (2 × 50 mL) to afford, after concentration in vacuo, *n*-hexane (60 mg), DCM (135 mg) and MeOH (65 mg) solubles. The DCM solubles, rich in target lipodepsipeptides, were subjected to gel chromatography (Sephadex^®^ LH-20 1.5 × 22 cm, isocratic gravity elution with MeOH). Fractions (10 mL) were monitored by UPLC-QTOF analysis to reveal fraction -2-1 (26 mg) as rich in target lipodepsipeptides, after which an aliquot (20 mg) was subjected to semi-preparative reversed-phase HPLC (Zorbax Eclipse SB-C_3_ 9.4 mm × 25 cm × 5 µm, 3 mL/min isocratic elution over 32 min with 45% H_2_O/MeCN and 0.1% TFA/MeCN) to yield scopularide A (**1**) (5 mg, 2.1%) and H (**8**) (3.8 mg, 1.6%) (Scheme S3). (Note: Percentage yields estimated on a mass-to-mass basis against the weight of the crude EtOAc extract partition).

### 3.6. Metabolite Charcterization

Scopularide A (**1**); white powder; [α]_D_^22.8^ − 34.9 (*c* 0.25, MeOH); NMR (600 MHz, MeOH-*d*_4_) see Table S1, Figures S6 and S7; NMR (600 MHz, DMSO-*d*_6_) see Table S2, Figures S8 and S9; ESI(+)MS *m*/*z* 672 [M + H]^+^; HRESI(+)MS *m*/*z* 694.4149 [M + Na]^+^ (calcd. for C_36_H_57_N_5_O_7_Na, 694.4150); GNPS library CCMSLIB00005436489.
Scopularide B (**2**); white powder; [α]_D_^22.2^ − 19.8 (*c* 0.05, MeOH); NMR (600 MHz, MeOH-*d*_4_) see Table S3, Figures S10 and S11; NMR (600 MHz, DMSO-*d*_6_) see Table S4, Figures S12 and S13; ESI(+)MS *m*/*z* 644 [M + H]^+^; HRESI(+)MS *m*/*z* 666.3842 [M + Na]^+^ (calcd. for C_34_H_53_N_5_O_7_Na, 666.3837); GNPS library CCMSLIB00005436490.
Scopularide C (**3**); white powder; [α]_D_^22.2^ − 32.0 (*c* 0.13, MeOH); NMR (600 MHz, DMSO-*d*_6_) see Table 1 and Table S5, Figures S14–S18; ESI(+)MS *m*/*z* 714 [M + H]^+^; HRESI(+)MS *m*/*z* 736.4638 [M + Na]^+^ (calcd. for C_39_H_63_N_5_O_7_Na, 736.4620); GNPS library CCMSLIB00005436483.
Scopularide D (**4**); white powder; [α]_D_^21.9^ − 40.9 (*c* 0.13, MeOH); NMR (600 MHz, DMSO-*d*_6_) see Table 1 and Table S6, Figures S19–S23; ESI(+)MS *m*/*z* 666 [M + H]^+^; HRESI(+)MS *m*/*z* 688.4620 [M + Na]^+^ (calcd. for C_35_H_63_N_5_O_7_Na, 688.4620); GNPS library CCMSLIB00005436484.
Scopularide E (**5**); white powder; [α]_D_^21.6^ − 19.2 (*c* 0.13, MeOH); NMR (600 MHz, DMSO-*d*_6_) see Table 1 and Table S7, Figures S24–S28; ESI(+)MS *m*/*z* 638 [M + H]^+^; HRESI(+)MS *m*/*z* 660.4313 [M + Na]^+^ (calcd. for C_33_H_59_N_5_O_7_Na, 660.4307); GNPS library CCMSLIB00005436485.
Scopularide F (**6**); white powder; [α]_D_^24.2^ − 17.5 (*c* 0.1, MeOH); NMR (600 MHz, DMSO-*d*_6_) see Table 2 and Table S8, Figures S29–S33; ESI(+)MS *m*/*z* 728 [M + H]^+^; HRESI(+)MS *m*/*z* 750.4787 [M + Na]^+^ (calcd. for C_40_H_65_N_5_O_7_Na, 750.4776); GNPS library CCMSLIB00005436486.
Scopularide G (**7**); white powder; ESI(+)MS *m*/*z* 680 [M + H]^+^; HRESI(+)MS *m*/*z* 702.4789 [M + Na]^+^ (calcd. for C_36_H_65_N_5_O_7_Na, 702.4776) GNPS library CCMSLIB00005436487.
Scopularide H (**8**); white powder; [α]_D_^22.2^ − 31.0 (*c* 0.15, MeOH); NMR (600 MHz, DMSO-*d*_6_) see Table 2 and Table S9, Figures S34–S38; ESI(+)MS *m*/*z* 700 [M + H]^+^; HRESI(+)MS *m*/*z* 722.4464 [M + Na]^+^ (calcd. for C_38_H_61_N_5_O_7_Na, 722.4463); GNPS library CCMSLIB00005436488.

### 3.7. C_3_ Marfey’s Analyses

Individual aliquots (50 μg) of **1**–**8** in 6 M HCl (100 μL) were heated to 100 °C in sealed vials for 8 to 12 h, after which the hydrolysates were concentrated to dryness at 40 °C under a stream of dry N_2_. The hydrolysates were then treated with 1 M NaHCO_3_ (20 μL) and l-FDAA (1% solution in acetone, 40 μL) at 40 °C for 1 h, after which each reaction was neutralized with 1 M HCl (20 μL) and filtered (0.45 μm PTFE) prior to HPLC-DAD-ESIMS analysis. An aliquot (10 μL) of each analyte was injected into an Agilent Zorbax SB-C_3_ column, 5 μm, 150 × 4.6 mm, 50 °C, with a 1 mL/min, 55 min linear gradient elution from 85% to 40% H_2_O/MeOH with a 5% isocratic modifier of 1% formic acid/MeCN. The analyte amino acid content was assessed by UV (340 nm) and ESI(±)MS monitoring, supported by SIE, with comparison to authentic standards. Amino acid standards were subjected to derivatization with l- and d-FDAA and HPLC-DAD-ESIMS analysis as described above for **1–8**.

### 3.8. Global Natural Product Social (GNPS) Molecular Networking

A GNPS analysis of ×63 Mugil mullet GIT-derived fungal isolates was carried out on UPLC-QTOF-(+)MS/MS data acquired on EtOAc crude extracts. Aliquots (1 μL) of test solutions comprising 100 μg/mL of analyte in 100 μL MeOH were analysed on an Agilent 6545 Q-TOF LC/MS equipped with an Agilent 1290 infinity II UPLC system, utilising an Agilent SB-C_8_ 1.8 μm, 2.1 × 50 mm column, 0.5 min isocratic elution of 90% H_2_O/MeCN followed by 4.5 min gradient elution to 100% MeCN with a flow rate of 0.5 mL/min and a constant isocratic 0.1% formic acid/MeCN modifier. UPLC-QTOF-(+)MS/MS data acquired for all samples at collision energy of 40 eV were converted from Agilent MassHunter data files (.d) to mzXML file format using MSConvert software, and transferred to the GNPS server (gnps.ucsd.edu). Molecular networking was performed using the GNPS data analysis workflow using the spectral clustering algorithm with a cosine score of 0.7 and a minimum of 6 matched peaks. The resulting spectral network was imported into Cytoscape version 3.5.1, where nodes corresponding to media components and solvent were subtracted. Remaining nodes represented parent (+) *m*/*z* of metabolites detected in analysed extracts, with node size indicating metabolite abundance and edge thickness corresponding to cosine scores (Figure 2, Figure 3, Figure 8, Figures S53 and S54). Figure S53 represents the molecular network for ×63 GIT-derived fungal isolates, with the “scopularides” cluster represented in Figure 2. Similarly, scopularides molecular family clusters detected in CMB-F585, CMB-F115 and CMB-F458 crude extracts are represented in Figure 3, Figure 8 and Figure S54.

The data were deposited to the GNPS-MassIVE Datasets and are publicly accessible through the links listed below:

Fish microbial library molecular networking
  MASSIVE: MSV000084190 (https://massive.ucsd.edu/ProteoSAFe/dataset.jsp?task=479a543e9b45408bbe93414769058784).
*GNPS Molecular Networking jobs:* (https://gnps.ucsd.edu/ProteoSAFe/status.jsp?task=1abcf2c49b9a43cf85e19b8573eeb8a7) (https://gnps.ucsd.edu/ProteoSAFe/status.jsp?task=2794a93e36724eaebafa0e1c3fb1aeb9) (https://gnps.ucsd.edu/ProteoSAFe/status.jsp?task=430bfc8c34974e418156d8c6e711bd35).
CMB-F585 molecular networking
  MASSIVE: MSV000084191 (https://massive.ucsd.edu/ProteoSAFe/dataset.jsp?task=4dee2595568c4296a1c4393de4758b24).
*GNPS Molecular Networking job:* (https://gnps.ucsd.edu/ProteoSAFe/status.jsp?task=ae56648aefa54f789e04cfc314cb59fc).
CMB-F115 molecular networking
  MASSIVE: MSV000084192 (https://massive.ucsd.edu/ProteoSAFe/dataset.jsp?task=74da14126ec24f2ca62ba443a05af7d2).
*GNPS Molecular Networking job:* (https://gnps.ucsd.edu/ProteoSAFe/status.jsp?task=e90bdf9596ff4bbfb9e4eb200435a2b1).
CMB-F458 molecular networking
  MASSIVE: MSV000084193 (https://massive.ucsd.edu/ProteoSAFe/dataset.jsp?task=4b0ec7ee53c34a299fe1828588ead84d).
*GNPS Molecular Networking:* (https://gnps.ucsd.edu/ProteoSAFe/status.jsp?task=3c2af674f0a248c39149993d91a8149e)

### 3.9. X-Ray Analysis of Scopularides C (***3***) and H (***8***)

Single crystals of both **3** and **8** were obtained by slow evaporation from 20% aqueous MeOH at room temperature. Crystallographic data (CuKα, 2θ_max_ = 125°) were collected on an Oxford Diffraction Gemini S Ultra CCD diffractometer with the crystal cooled to 190 K with an Oxford Cryosystems Desktop Cooler. Data reduction and empirical absorption corrections were carried out with the CrysAlisPro program (Oxford Diffraction vers. 171.38.46). The structures were solved by direct methods with SHELXT and refined with SHELXL [21]. The thermal ellipsoid diagrams were generated with ORTEP3 [22], while Figure 7 and Figure 9 were generated by Mercury 4.1.0. All crystallographic calculations were carried out within the WinGX graphical user interface [23]. The crystal structures have been deposited in the CCDC database (scoupularide C (**3**): CCDC 1936745 and scopularide H (**8**): CCDC 1936746) (Figure 7, Figure 9, Figures S55 and S56).

### 3.10. Antibacterial Assay

The bacterium to be tested was streaked onto a tryptic soy agar plate and was incubated at 37 °C for 24 h. One colony was then transferred to fresh tryptic soy broth (15 mL) and the cell density was adjusted to 10^4^–10^5^ CFU/mL. The compounds to be tested were dissolved in DMSO and diluted with H_2_O to give 600 µM stock solution (20% DMSO), which was serially diluted with 20% DMSO to give concentrations from 600 to 0.2 µM in 20% DMSO. An aliquot (10 µL) of each dilution was transferred to a 96-well microtiter plate and freshly prepared microbial broth (190 µL) was added to each well to give final concentrations of 30 to 0.01 µM in 1% DMSO. The plates were incubated at 37 °C for 24 h and the optical density of each well was measured spectrophotometrically at 600 nm using POLARstar Omega plate (BMG LABTECH, Offenburg, Germany). Each test compound was screened against the Gram −ve bacteria *Escherichia coli* ATCC 11775 and the Gram +ve bacteria *Staphylococcus aureus* ATCC 9144 and three clinical isolates of methicillin-susceptible *Staphylococcus aureus*, methicillin-resistant *Staphylococcus aureus* and vancomycin-resistant *Enterococcus faecalis*. Rifampicin, ampicillin, daptomycin and methicillin were used as a positive control (30 µM in 10% DMSO). The IC_50_ value was calculated as the concentration of the compound or antibiotic required for 50% inhibition of the bacterial cells using Prism 7.0 (GraphPad Software Inc., La Jolla, CA, USA).

### 3.11. Antifungal Assay

The fungus *Candida albicans* ATCC 10231 was streaked onto a Sabouraud agar plate and was incubated at 37 °C for 48 h. One colony was then transferred to fresh Sabouraud broth (15 mL) and the cell density adjusted to 10^4^–10^5^ CFU/mL. Test compounds were dissolved in DMSO and diluted with H_2_O to give a 600 µM stock solution (20% DMSO), which was serially diluted with 20% DMSO to give concentrations from 600 to 0.2 µM in 20% DMSO. An aliquot (10 µL) of each dilution was transferred to a 96-well microtiter plate and freshly prepared fungal broth (190 µL) was added to each well to give final concentrations of 30 to 0.01 µM in 1% DMSO. The plates were incubated at 37 °C for 24 h and the optical density of each well was measured spectrophotometrically at 600 nm using POLARstar Omega plate (BMG LABTECH, Offenburg, Germany). Ketoconazole was used as a positive control (30 µg/mL in 10% DMSO). Where relevant, IC_50_ value were calculated as the concentration of the compound or antifungal drug required for 50% inhibition of the fungal cells using Prism 7.0 (GraphPad Software Inc., La Jolla, CA, USA).

### 3.12. Cytotoxicity Assay

Adherent SW620 (human colorectal carcinoma) and NCI-H460 (human lung carcinoma) cells were cultured in Roswell Park Memorial Institute (RPMI) 1640 medium, while HepG2 (adherent human hepatocellular carcinoma) cells were cultured in Dulbecco’s modified Eagle’s medium (DMEM). All cells were cultured as adherent mono-layers in flasks supplemented with 10% foetal bovine serum, l-glutamine (2 mM), penicillin (100 unit/mL) and streptomycin (100 µg/mL), in a humidified 37 °C incubator supplied with 5% CO_2_. Briefly, cells were harvested with trypsin and dispensed into 96-well microtiter assay plates at 3000 cells/well, after which they were incubated for 18 h at 37 °C with 5% CO_2_ (to allow cells to attach as adherent mono-layers). Test compounds were dissolved in 20% DMSO in PBS (*v*/*v*) and aliquots (10 µL) applied to cells over a series of final concentrations ranging from 10 nM to 30 µM. After 48 h incubation at 37 °C with 5% CO_2_, an aliquot (10 µL) of 3-(4,5-dimethylthiazol-2-yl)-2,5-diphenyltetrazolium bromide (MTT) in phosphate buffered saline (PBS, 5 mg/mL) was added to each well (final concentration 0.5 mg/mL), and microtiter plates were incubated for a further 4 h at 37 °C with 5% CO_2_. After final incubation, the medium was aspirated and precipitated formazan crystals dissolved in DMSO (100 µL/well). The absorbance of each well was measured at 600 nm with a POLARstar Omega plate (BMG LABTECH, Offenburg, Germany). Where relevant, IC_50_ values were calculated using Prism 8.0, as the concentration of analyte required for 50% inhibition of cancer cell growth (compared to negative controls). Negative control was 1% aqueous DMSO, while positive control was vinblastine (30 µM). All experiments were performed in duplicate.

## Figures and Tables

**Table 1:** 1D NMR (600 MHz, DMSO-*d*_6_) data for scopularides C–E (**3**–**5**).

	(3)		(4)		(5)	
Position	δ_H_, mult (*J* in Hz)	δ_C_	δ_H_, mult (*J* in Hz)	δ_C_	δ_H_, mult (*J* in Hz)	δ_C_
	l-Phe^1^		l-Val^1^		l-Ala^1^	
1	---	170.8	---	170.7	---	171.7 ^g^
2	4.38, q (*7.1*)	54.2	4.15, dd (*7.8, 5.3*)	57.3	4.13, dq (*7.4, 6.0*)	48.1
3	a 3.01, dd (*13.9, 6.1*) b 2.93, d (*13.9, 8.7*)	36.7	2.08, m	29.7	1.28, d (*7.4*)	16.7
4	---	137.2	0.87, d (*6.8*)	19.0		
5/9	7.25, m	129.0	0.85^a^, d (*7.0*)	17.4		
6/8	7.28, m	128.2				
7	7.20, m	126.5				
NH	7.96, d (*7.1*)	---	7.41, d (*7.1*)	---	7.80, d (*6.0*)	---
	l-Ala^2^		l-Ala^2^		l-Ala^2^	
1	---	171.7^f^	---	171.8	---	171.5
2	4.16, dq (*7.4, 7.1*)	47.6	4.19, dq (*8.0, 7.1*)	47.9	4.18, dq (*7.1, 6.5*)	47.5
3	1.15, d (*7.1*)	17.6	1.21, d (*7.1*)	17.5	1.19, d (*7.1*)	17.4
NH	7.84, d (*7.4*)	---	8.03, d (*8.0*)	---	7.89, d (*6.5*)	---
	d-Leu^3^		d-Leu^3^		d-Leu^3^	
1	---	171.0	---	171.3	---	171.1
2	4.03, m	52.0	4.04, dd (*10.1, 6.5*)	51.9	4.03, dd (*11.4, 6.3*)	51.8
3	1.46, m	38.7	1.48, m	38.7	a 1.49, m b 1.47 ^a^, m	38.6
4	1.63, m	24.1	1.63, m	24.1	1.63, m	24.1
5	0.88, d (*6.5*)	22.9	0.89 ^b^, d (*6.7*)	22.9	0.89, d (*6.7*)	23.0
6	0.81, d (*6.6*)	21.0	0.81, d (*6.6*)	21.0	0.81, d (*6.5*)	21.1
NH	8.62, d (*6.1*)	---	8.63, d (*6.5*)	---	8.64, d (*6.3*)	---
	l-Val^4^		l-Val^4^		l-Val^4^	
1	---	171.7^f^	---	171.6	---	171.6 ^g^
2	4.10, dd (*8.6, 7.6*)	58.3	4.06 ^c^, m	58.6	4.11, dd (*8.2, 6.6*)	58.1
3	1.88, m	29.8	1.86, m	29.5	1.85, m	29.9
4	0.87, d (*6.4*)	18.9	0.88 ^b^, d (*6.8*)	19.0	0.87, d (*6.9*)	18.8
5	0.83, d (*6.6*)	18.7	0.83 ^d^, d (*6.8*)	18.7	0.83, d (*6.7*)	18.7
NH	7.92 ^a^, d (*7.6*)	---	8.06, d (*7.8*)	---	7.87, d (*6.6*)	---
	Gly^5^		Gly^5^		Gly^5^	
1	---	168.9	---	169.0	---	168.8
2	a 4.07, dd (*16.7, 6.6*) b 3.41, dd (*16.7, 3.9*)	42.3	a 4.06 ^c^, m b 3.44, dd (*17.1, 3.8*)	42.0	a 4.06, dd (*16.5, 6.6*) b 3.43, dd (*16.5, 4.0*)	42.3
NH	7.91 ^a^, dd (*6.5, 3.9*)	---	7.90, dd (*5.8, 3.8*)	---	7.82, dd (*6.1, 4.0*)	---
	HDMLA		HDMLA		HDMLA	
1′	---	169.8	---	170.0	---	169.8
2′	a 2.51, dd (*15.2, 9.8*) b 2.25, d (*15.2, 1.4*)	37.7	a 2.52, dd (*14.5, 9.6*) b 2.23, dd (*14.5, 1.4*)	38.0	a 2.53, dd (*14.7, 10.1*) b 2.24, dd (*14.7, 1.8*)	37.7
3′	4.94, ddd (*9.1, 5.1, 1.8*)	74.8	4.92, ddd (*9.3, 4.8, 1.4*)	75.2	4.97, ddd (*10.1, 4.8, 1.8*)	74.2
4′	1.68, m	33.7	1.79, m	33.9	1.75, m	33.8
5′	a 1.18 ^b^, m b 0.75, m	39.5^h^	a 1.29, m b 0.87, m	39.6^h^	a 1.26, m b 0.84, m	39.6 ^h^
6′	1.41, m	29.3	1.44, m	29.3	1.46 ^a^, m	29.2
7′	a 1.19 ^b^, m b 0.95, m	35.7	a 1.22 ^e^, m b 0.98, m	36.0	a 1.23 ^b^, m b 0.99, m	35.8
8′	1.20 ^b^, m	26.1	a 1.26, m b 1.16, m	26.1	a 1.24, m b 1.16, m	26.1
9′	1.22 ^c^, m	29.1	a 1.22 ^e^, m b 1.19, m	29.1	1.23 ^b^, m	29.0
10′	1.22 ^c^, m	31.3	1.23 ^e^, m	31.3	1.23 ^b^, m	31.3
11′	1.24, m	22.1	1.25, m	22.1	1.26, m	22.1
12′	0.84, t (*6.9*)	13.9	0.85 ^a^, t (*7.9*)	13.9	0.85, t (*7.1*)	13.9
4′-Me	0.70, d (*6.7*)	14.8	0.84 ^d^, d (*8.0*)	15.1	0.82, d (*6.4*)	15.0
6′-Me	0.80, d (*6.6*)	20.2	0.83 ^d^, d (*7.4*)	20.0	0.83, d (*6.7*)	20.2

^a–e^ Overlapping resonances within the same letter and column, ^f-g^ assignments are interchangeable within the same letter and column, ^h^ obscured by solvent. HDMLA = 3*S*,4*S*,6*S*-3-hydroxyl-4,6-dimethyllauric acid

**Table 2:** 1D NMR (600 MHz, DMSO-*d*_6_) data for scopularides F and H (**6** and **8**).

	(6)		(8)	
Position	δ_H_, mult (*J* in Hz)	δ_C_	δ_H_, mult (*J* in Hz)	δ_C_
	l-Phe^1^		l-Phe^1^	
1	---	170.9	---	170.8
2	4.38, m	54.3	4.34, q (*7.4*)	54.5
3	a 2.97, dd (*13.9, 6.1*) b 2.95, dd (*13.9, 8.4*)	36.7	2.98, m	36.7
4	---	137.1	---	137.2
5/9	7.26, m	128.9	7.26, m	129.1
6/8	7.28, m	128.3	7.26, m	128.2
7	7.21, m	126.6	7.20, m	126.5
NH	8.14, d (*6.5*)	---	8.03, d (*6.7*)	---
	l-Abu^2^		l-Ala^2^	
1	---	171.0	---	171.8
2	4.08 ^a^, m	52.9	4.19, dq (*8.1, 7.1*)	47.6
3	a 1.74, m b 1.48 ^b^, m	24.4	1.15, d (*7.1*)	17.6
4	0.73, t *(7.2)*	9.5		
NH	7.67, d (*8.0*)	---	7.83, d (*8.1*)	---
	d-Leu^3^		d-Leu^3^	
1	---	171.1	---	171.0
2	4.09 ^a^, m	51.8	4.03, m	52.0
3	1.47 ^b^, m	38.6	a 1.47, m b 1.44, m	38.7
4	1.61, m	24.1	1.63, m	24.1
5	0.88, d (*6.5*)	22.8	0.89, d (*6.5*)	22.9
6	0.81, d (*6.8*)	21.2	0.82 ^d^, d (*6.5*)	21.1
NH	8.62, d (*6.3*)	---	8.63, d (*6.0*)	---
	l-Val^4^		l-Val^4^	
1	---	171.6	---	171.7
2	4.15, dd (*8.9, 8.7*)	58.1	4.10, dd (*8.6, 7.9*)	58.3
3	1.88, m	29.9	1.87, m	29.8
4	0.86, d (*6.7*)	18.8	0.88 ^c^, d (*6.2*)	18.9
5	0.83 ^f^, d (*6.6*)	18.7	0.81 ^d^, d (*6.1*)	18.7
NH	7.86, d (*8.9*)	---	7.88, d (*7.9*)	---
	Gly^5^		Gly^5^	
1	---	168.6	---	168.9
2	a 4.01, dd (*16.7, 6.3*) b 3.42, dd (*16.7, 4.7*)	42.4	a 4.07, dd (*16.6, 6.4*) b 3.40, dd (*16.6, 4.1*)	42.3
NH	7.85, dd (*5.6, 4.7*)	---	7.93, dd (*6.4, 4.1*)	---
	HDMLA		HMLA	
1′	---	169.8	---	169.8
2′	a 2.53, dd (*14.8, 9.4*) b 2.24, dd (*14.8, 1.2*)	37.9	a 2.48, dd (*14.9, 9.2*) b 2.24, dd (*14.9, 1.8*)	37.0
3′	4.94, ddd (*9.1, 3.2, 1.8*)	74.7	4.90, ddd (*9.2, 5.7, 1.8*)	75.1
4′	1.66, m	33.9	1.50, m	36.1
5’	a 1.17 ^e^, m b 0.75, m	39.6^f^	a 1.19 ^b^, m b 0.87 ^c^, m	31.4
6′	1.41, m	29.2	a 1.19 ^b^, m b 1.09, m	26.5
7′	a 1.19, m b 0.95, m	35.8	1.18 ^b^, m	29.3
8′	a 1.25 ^c^, m b 1.17 ^e^, m	26.1	1.23 ^a^, m	28.9
9′	1.22 ^d^, m	29.1	1.23 ^a^, m	28.7
10′	1.22 ^d^, m	31.3	1.23 ^a^, m	31.3
11′	1.24 ^c^, m	22.1	1.25, m	22.1
12′	0.83 ^f^_,_ t (*6.9*)	13.9	0.85, t (*6.6*)	13.9
4′-Me	0.69, d (*6.7*)	14.8	0.67, d (*6.9*)	14.5
6′-Me	0.80, d (*6.8*)	20.1		

^a–e^ Overlapping resonances within the same letter and column, ^f^ obscured by solvent. HDMLA = 3*S*,4*S*,6*S*-3-hydroxyl-4,6-dimethyllauric acid. HMLA = 3*S*,4*S*-3-hydroxyl-4-methyllauric acid.

**Table 3:**
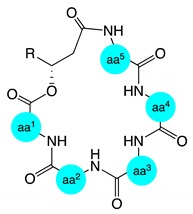
Amino acid sequence homology for natural lipodepsipeptides related to scopularides.

Compound	aa^1^	aa^2^	aa^3^	aa^4^	aa^5^	Compound	aa^1^	aa^2^	aa^3^	aa^4^	aa^5^
scopularide A [2]	l-Phe	l-Ala	d-Leu	l-Val	Gly	iso-isariin D [17]	l-Ala	l-Ala	d-Leu	l-Val	Gly
scopularide B [2]	l-Phe	l-Ala	d-Leu	l-Val	Gly	isariin G2 [15]	l-Ala	l-Ala	d-Leu	l-Val	Gly
scopularide C	l-Phe	l-Ala	d-Leu	l-Val	Gly	scopularide E	l-Ala	l-Ala	d-Leu	l-Val	Gly
scopularide H	l-Phe	l-Ala	d-Leu	l-Val	Gly	isariin F1 [15]	Abu/Aib	l-Ala	d-Leu	l-Val	Gly
chrysogeamide A [6]	l-Val	l-Ala	d-Leu	l-Val	Gly	scopularide F	l-Phe	l-Abu	d-Leu	l-Val	Gly
chrysogeamide B [6]	l-Val	l-Ala	d-Leu	l-Val	Gly	chrysogeamide C [6]	l-Phe	l-Ala	d-Leu	d-Leu	Gly
scopularide D	l-Val	l-Ala	d-Leu	l-Val	Gly	chrysogeamide D [6]	l-Phe	l-Ala	d-Leu	d-Leu	Gly
nodupetide [9]	l-Val	l-Ala	d-Leu	l-Val	Gly	chrysogeamide E [6]	l-Phe	l-Ala	d-Leu	d-Leu	Gly
isariin A [10,11]	l-Val	l-Ala	d-Leu	l-Val	Gly	chrysogeamide F [6]	l-Phe	l-Ala	d-Leu	l-Pro	Gly
isariin B [12,13,14]	l-Val	l-Ala	d-Leu	l-Val	Gly	chrysogeamide G [6]	l-Phe	l-Ala	d-Leu	l-Pro	Gly
iso-isariin B [16]	l-Val	l-Ala	d-Leu	l-Val	Gly	scopularide G	l-Val	l-Abu	d-Leu	l-Val	Gly
isariin C2 [15]	l-Val	l-Ala	d-Leu	l-Val	Gly	emericellamide A [7]	l-Ala	l-Ala	l-Leu	l-Val	Gly
isariin E [15]	l-Val	l-Ala	d-Leu	l-Val	Gly	emericellamide B [7]	l-Ala	l-Ala	l-Leu	l-Val	Gly
isariin F2 [15]	l-Val	l-Ala	d-Leu	l-Val	Gly	emericellamide C [8]	l-Ala	l-Ala	l-Leu	l-Val	Gly
isariin G1 [15]	l-Val	l-Ala	d-Leu	l-Val	Gly	emericellamide D [8]	l-Ala	l-Ala	l-Leu	l-Val	Gly
oryzamide A [5]	l-Leu	l-Ala	d-Leu	l-Val	Gly	emericellamide E [8]	l-Ala	l-Ala	l-Leu	l-Val	Gly
oryzamide B [5]	l-Tyr	l-Ala	d-Leu	l-Val	Gly	emericellamide F [8]	l-Ala	l-Ala	l-Leu	l-Val	Gly
oryzamide C [5]	l-Met	l-Ala	d-Leu	l-Val	Gly	arenamide A [18]	l-Phe	l-Ala	l-Leu	l-Val	Gly
isariin C [13,14]	l-Ala	l-Ala	d-Leu	l-Val	Gly	arenamide B [18]	l-Phe	l-Ala	l-Leu	l-Val	Gly
isariin D [13,14]	l-Ala	l-Ala	d-Leu	l-Val	Gly	arenamide C [18]	l-Met	l-Ala	l-Leu	l-Val	Gly

**Table 4:**
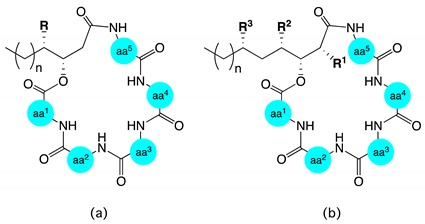
Lipid residue homology for natural lipodepsipeptides related to scopularides.

**Structure a**									
**compound**	**n**	**R**			**compound**	**n**	**R**		
isariin E * [15]	1	H			iso-isariin B * [16]	3	Me		
iso-isariin D [17]	1	Me			isariin B [12,13,14]	4	H		
nodupetide [9]	1	Me			isariin C * [13,14]	4	H		
chrysogeamide D [6]	1	Me			isariin F1* [15]	4	H		
isariin C2 * [15]	2	H			isariin G1 * [15]	5	H		
isariin D * [13,14]	2	H			isariin A [10,11,19]	7	H		
isariin F2 * [15]	3	H			isariin G2 * [15]	7	H		
**Structure b**									
**compound**	**n**	**R^1^**	**R^2^**	**R^3^**	**compound**	**n**	**R^1^**	**R^2^**	**R^3^**
scopularide B [2]	1	H	Me	H	arenamide A [18,20]	3	H	Me	H
chrysogeamide A [6]	1	H	Me	H	arenamide C [18,20]	3	H	Me	H
chrysogeamide E [6]	1	H	Me	H	emericellamide A [7]	3	Me	Me	H
chrysogeamide F [6]	1	H	Me	H	emericellamide C [8]	3	Me	H	H
arenamide B [18,20]	1	H	Me	H	emericellamide E [8]	5	Me	H	H
scopularide A [2]	3	H	Me	H	emericellamide F [8]	5	H	Me	H
chrysogeamide B [6]	3	H	Me	H	scopularide H	5	H	Me	H
chrysogeamide C [6]	3	H	Me	H	scopularide C	5	H	Me	Me
chrysogeamide G [6]	3	H	Me	H	scopularide D	5	H	Me	Me
oryzamide A [5]	3	H	Me	H	scopularide E	5	H	Me	Me
oryzamide B [5]	3	H	Me	H	scopularide F	5	H	Me	Me
oryzamide C [5]	3	H	Me	H	scopularide G	5	H	Me	Me
emericellamide D [8]	3	H	Me	H	emericellamide B [7]	5	Me	Me	Me

* Absolute configuration of C-3′ and/or C-4′ was not assigned.

## Supplementary Materials

The following are available online at https://www.mdpi.com/1660-3397/17/8/475/s1, MS/MS fragmentations, Marfey’s analyses, together with tabulated 1D and 2D NMR data, annotated 1D NMR and selected 2D NMR spectra, and bioassay data.

## References

[1] Mohamed O.G., Khalil Z.G., Capon R.J. (2018). Prolinimines: N-Amino-L-Pro-methyl ester (hydrazine) Schiff bases from a fish gastrointestinal tract-derived fungus, *Trichoderma* sp. CMB-F563. Org. Lett..

[2] Yu Z., Lang G., Kajahn I., Schmaljohann R., Imhoff J.F. (2008). Scopularides A and B, cyclodepsipeptides from a marine sponge-derived fungus, *Scopulariopsis brevicaulis*. J. Nat. Prod..

[3] Wang M., Carver J.J., Phelan V.V., Sanchez L.M., Garg N., Peng Y., Nguyen D.D., Watrous J., Kapono C.A., Luzzatto-Knaan T. (2016). Sharing and community curation of mass spectrometry data with Global Natural Products Social Molecular Networking. Nat. Biotechnol..

[4] Vijayasarathy S., Prasad P., Fremlin L.J., Ratnayake R., Salim A.A., Khalil Z., Capon R.J. (2016). C_3_ and 2D C_3_ Marfey’s methods for amino acid analysis in natural products. J. Nat. Prod..

[5] Ding L.-J., Yuan W., Liao X.-J., Han B.-N., Wang S.-P., Li Z.-Y., Xu S.-H., Zhang W., Lin H.-W. (2016). Oryzamides A–E, cyclodepsipeptides from the sponge-derived fungus *Nigrospora oryzae* PF18. J. Nat. Prod..

[6] Hou X.-M., Li Y.-Y., Shi Y.-W., Fang Y.W., Chao R., Gu Y.-C., Wang C.-Y., Shao C.-L. (2019). Integrating molecular networking and ^1^H NMR to target the isolation of chrysogeamides from a library of marine-derived *Penicillium* fungi. J. Org. Chem..

[7] Oh D.-C., Kauffman C.A., Jensen P.R., Fenical W. (2007). Induced production of emericellamides A and B from the marine-derived fungus *Emericella* sp. in competing co-culture. J. Nat. Prod..

[8] Chiang Y.-M., Szewczyk E., Nayak T., Davidson A.D., Sanchez J.F., Lo H.-C., Ho W.-Y., Simityan H., Kuo E., Praseuth A. (2008). Molecular genetic mining of the *Aspergillus* secondary metabolome: Discovery of the emericellamide biosynthetic pathway. Chem. Biol..

[9] Wu H.M., Lin L.P., Xu Q.L., Han W.B., Zhang S., Liu Z.W., Mei Y.N., Yao Z.-J., Tan R.X. (2017). Nodupetide, a potent insecticide and antimicrobial from *Nodulisporium* sp. associated with *Riptortus pedestris*. Tetrahedron Letts..

[10] Vining L.C., Taber W.A. (1962). Isariin, a new depsipeptide from *Isaria cretacea*. Can. J. Chem..

[11] Ióca L.P., Nicacio K.J., Berlinck R.G.S., Ióca L.P., Nicacio K.J., Berlinck R.G.S. (2018). Natural Products from marine invertebrates and microorganisms in Brazil between 2004 and 2017: Still the challenges, more rewards. J. Braz. Chem. Soc..

[12] Lira S.P., Vita-marques A.M., Seleghim M.H.R., Bugni T.S., Labarbera D.V., Sette L.D., Sponchiado S.R.P., Ireland C.M., Berlinck R.G.S. (2006). New destruxins from the marine-derived fungus *Beauveria felina*. J. Antibiot..

[13] Baute R., Deffieux G., Merlet D., Baute M.A., Neveu A. (1981). New insecticidal cyclodepsipeptides from the fungus *Isaria felina*: I. Production, isolation and insecticidal properties of isariins B, C and D. J. Antibiot..

[14] Deffieux G., Merlet D., Baute R., Bourgeois G., Baute M.A., Neveu A. (1981). New insecticidal cyclodepsipeptides from the fungus *Isaria felina* II. Structure elucidation of isariins B, C and D. J. Antibiot..

[15] Sabareesh V., Ranganayaki R.S., Raghothama S., Bopanna M.P., Balaram H., Srinivasan M.C., Balaram P. (2007). Identification and characterization of a library of microheterogeneous cyclohexadepsipeptides from the fungus *Isaria*. J. Nat. Prod..

[16] Langenfeld A., Blond A., Gueye S., Herson P., Nay B., Dupont J., Prado S. (2011). Insecticidal cyclodepsipeptides from *Beauveria felina*. J. Nat. Prod..

[17] Du F.-Y., Li X.-M., Zhang P., Li C.-S., Wang B.-G. (2014). Cyclodepsipeptides and other O-containing heterocyclic metabolites from *Beauveria felina* EN-135, a marine-derived entomopathogenic fungus. Mar. Drugs.

[18] Asolkar R.N., Freel K.C., Jensen P.R., Fenical W., Kondratyuk T.P., Park E.-J., Pezzuto J.M. (2009). Arenamides A–C, cytotoxic NFkappaB inhibitors from the marine actinomycete *Salinispora arenicola*. J. Nat. Prod..

[19] Hardy P.M., Prout R.A., Rydon H.N. (1974). Polypeptides. Part XXV. Synthesis of isariin. J. Chem. Soc. Perkin Trans. 1.

[20] Chandrasekhar S., Pavankumarreddy G., Sathish K. (2009). Total synthesis of arenamide A and its diastereomer. Tetrahedron Letts..

[21] Sheldrick G.M. (2008). A short history of SHELX. Acta Crystallogr. Sect. A Found. Crystallogr..

[22] Farrugia L.J. (1997). ORTEP-3 for Windows—A version of ORTEP-III with a Graphical User Interface (GUI). J. Appl. Crystallogr..

[23] Farrugia L.J. (1999). WinGX suite for small-molecule single-crystal crystallography. J. Appl. Crystallogr..

