# Supplementary material for: Scopularides Revisited: Molecular Networking Guided Exploration of Lipodepsipeptides in Australian Marine Fish Gastrointestinal Tract-Derived Fungi
Source: Mar Drugs. 2019 Aug 16;17(8):475. doi: 10.3390/md17080475 (PMC6722657; doi:10.3390/md17080475)
Supplement: Supplementary file 1 [file marinedrugs-17-00475-s001.pdf]

Supplementary Material

Scopularides revisited: Molecular networking guided exploration of lipodepsipeptides in Australian marine fish gastrointestinal tract-derived fungi

Ahmed H. Elbanna,[1] Zeinab G. Khalil,[1] Paul V. Bernhardt[2] and Robert J. Capon[1*]

[1]Institute for Molecular Bioscience, The University of Queensland, St Lucia, QLD, Australia

[2]School of chemistry and Molecular Bioscience, The University of Queensland, St Lucia, QLD, Australia

*Corresponding author. Tel.: +61 7 3346 2979; fax: +61 7 3346 2090; e-mail: r.capon@uq.edu.au

Table of content

Table of content i
List of figures ii
List of tables iv
List of schemes iv
1 Experimental 1
1.1 Fungal isolation and DNA taxonomic analysis 1
1.1.1 CMB-F458 1
1.1.2 CMB-F585 3
1.1.3 CMB-F115 4
1.2 Phylogenetic tree 5
1.3 Cultivation of the fungal strains, production and isolation of compounds **1-8** 7
2 Spectroscopic characterization of metabolites 1-6 and 8 8
2.1 Scopularide A (**1**) 8
2.2 Scopularide B (**2**) 14
2.3 Scopularide C (**3**) 20
2.4 Scopularide D (**4**) 24
2.5 Scopularide E (**5**) 28
2.6 Scopularide F (**6**) 32
2.7 Scopularide H (**8**) 36
3 MS/MS fragmentation of scopularides C-H (3-8) 40
4 Marfey's analysis of scopularides A-H (1-8) 46
5 GNPS molecular networking 50
5.1 GNPS networking of fish crude extract library 50
5.2 GNPS networking of CMB-F458 50
6 X-ray analysis of scopularide C (3) and scopularide H (8) 51
6.1 Scopularide C (**3**) 51
6.2 Scopularide H (**8**) 57
7 Biological assays 61
7.1 Antibacterial and antifungal assays 61
7.2 Cytotoxicity assay 62
8 References 63

List of figures

Figure S1. CMB-F458 (a), CMB-F585 (b) and CMB-F115 (c) fungal strains cultured on M1S agar plates....1
Figure S2. Blast search (closest match) for CMB-F458....2
Figure S3. Blast search (closest match) for CMB-F585....4
Figure S4. Blast search (closest match) for CMB-F115....5
Figure S5. Phylogenetic tree by PhyML Maximum Likehood analysis of 18s rRNA sequences showing the relationship of CMB-F458, CMB-F585 and CMB-F115 among selected reference strains (RefSeq GenBank) with their accession numbers....6
Figure S6. ^{1}H NMR (MeOH-d_4) spectra for a) scopularide A (**1**) (600 MHz) compared to b) reported scopularide A....9
Figure S7. ^{13}C NMR (MeOH-d_4) spectra for a) scopularide A (**1**) (150 MHz) compared to b) reported scopularide A....10
Figure S8. ^{1}H NMR (DMSO-d_6) spectrua for scopularide A (**1**) compared to b) reported arenamide A....12
Figure S9. ^{13}C NMR (DMSO-d_6) spectra for scopularide A (**1**) compared to b) reported arenamide A....13
Figure S10. ^{1}H NMR (MeOH-d_4) spectra for scopularide B (**2**) compared to b) reported scopularide B....15
Figure S11. ^{13}C NMR (MeOH-d_4) spectra for scopularide B (**2**) compared to b) reported scopularide B....16
Figure S12. ^{1}H NMR (DMSO-d_6) spectrum for scopularide B (**2**) compared to b) reported arenamide B....18
Figure S13. ^{13}C NMR (DMSO-d_6) spectrum for scopularide B (**2**) compared to b) reported arenamide B....19
Figure S14. ^{1}H NMR (DMSO-d_6) spectrum for scopularide C (**3**)....21
Figure S15. ^{13}C NMR (DMSO-d_6) spectrum for scopularide C (**3**)....21
Figure S16. HSQC NMR (DMSO-d_6) spectrum for scopularide C (**3**)....22
Figure S17. HMBC NMR (DMSO-d_6) spectrum for scopularide C (**3**)....22
Figure S18. COSY NMR (DMSO-d_6) spectrum for scopularide C (**3**)....23
Figure S19. ^{1}H NMR (DMSO-d_6) spectrum for scopularide D (**4**)....25
Figure S20. ^{13}C NMR (DMSO-d_6) spectrum for scopularide D (**4**)....25
Figure S21. HSQC NMR (DMSO-d_6) spectrum for scopularide D (**4**)....26
Figure S22. HMBC NMR (DMSO-d_6) spectrum for scopularide D (**4**)....26
Figure S23. COSY NMR (DMSO-d_6) spectrum for scopularide D (**4**)....27
Figure S24. ^{1}H NMR (DMSO-d_6) spectrum for scopularide E (**5**)....29
Figure S25. ^{13}C NMR (DMSO-d_6) spectrum for scopularide E (**5**)....29
Figure S26. HSQC NMR (DMSO-d_6) spectrum for scopularide E (**5**)....30
Figure S27. HMBC NMR (DMSO-d_6) spectrum for scopularide E (**5**)....30
Figure S28. COSY NMR (DMSO-d_6) spectrum for scopularide E (**5**)....31
Figure S29. ^{1}H NMR (DMSO-d_6) spectrum for scopularide F (**6**)....33
Figure S30. ^{13}C NMR (DMSO-d_6) spectrum for scopularide F (**6**)....33
Figure S31. HSQC NMR (DMSO-d_6) spectrum for scopularide F (**6**)....34
Figure S32. HMBC NMR (DMSO-d_6) spectrum for scopularide F (**6**)....34
Figure S33. COSY NMR (DMSO-d_6) spectrum for scopularide F (**6**)....35
Figure S34. ^{1}H NMR (DMSO-d_6) spectrum for scopularide H (**8**)....37
Figure S35. ^{13}C NMR (DMSO-d_6) spectrum for scopularide H (**8**)....37
Figure S36. HSQC NMR (DMSO-d_6) spectrum for scopularide H (**8**)....38
Figure S37. HMBC NMR (DMSO-d_6) spectrum for scopularide H (**8**)....38

Figure S38. COSY NMR (DMSO-d_6) spectrum for scopularide H (**8**)................................39
Figure S39. MS/MS fragmentation of scopularide C (**3**)..40
Figure S40. MS/MS fragmentation of scopularide D (**4**) 41
Figure S41. MS/MS fragmentation of scopularide E (**5**)..42
Figure S42. MS/MS fragmentation of scopularide F (**6**)..43
Figure S43. MS/MS fragmentation of scopularide G (**7**) 44
Figure S44. MS/MS fragmentation of a) scopularide G (**7**) compared to b) scopularide F (**6**) 44
Figure S45. MS/MS fragmentation of scopularide H (**8**) 45
Figure S46. Marfey's analysis of scopularide A (**1**)...46
Figure S47. Marfey's analysis of scopularide B (**2**)...46
Figure S48. Marfey's analysis of scopularide C (**3**)...47
Figure S49. Marfey's analysis of scopularide D (**4**)...47
Figure S50. Marfey's analysis of scopularide E (**5**)...48
Figure S51. Marfey's analysis of scopularide F (**6**) 48
Figure S52. Marfey's analysis of scopularide G (**7**)...49
Figure S53. GNPS molecular network for a comparative analysis of ×63 Mugil mullet GIT-derived fungal extracts...50
F**igure S54.** GNPS cluster for scopularides detected in a M1S solid phase cultivation of *Scopulariopsis* sp. CMB-F458 50
Figure S55. X-ray crystal structure of scopularide C (**3**)..51
Figure S56. X-ray crystal structure of scopularide H (**8**)..57
Figure S57. Growth inhibitory activity of scopularides A-H (**1**-**8**) 61
Figure S58. Cytotoxicity assay of the scopularides A-H (**1**-**8**) 62

List of tables

Table S1. NMR (600 MHz, MeOH-d_4) data for scopularide A (**1**)..8
Table S2. NMR (600 MHz, DMSO-d_6) data for scopularide A (**1**) 11
Table S3. NMR (600 MHz, MeOH-d_4) data for scopularide B (**2**) 14
Table S4. NMR (600 MHz, DMSO-d_6) data for scopularide B (**2**)......................................17
Table S5. NMR (600 MHz, DMSO-d_6) data for scopularide C (**3**)......................................20
Table S6. NMR (600 MHz, DMSO-d_6) data for scopularide D (**4**) 24
Table S7. NMR (600 MHz, DMSO-d_6) data for scopularide E (**5**)......................................28
Table S8. NMR (600 MHz, DMSO-d_6) data for scopularide F (**6**)......................................32
Table S9. NMR (600 MHz, DMSO-d_6) data for scopularide H (**8**) 36
Table S10. Crystal data and structure refinement for scopularide C (**3**)...............................52
Table S11. Crystal data and structure refinement for scopularide H (**8**) 58

List of schemes

Scheme S1. Isolation scheme of scopularides A (**1**) and B (**2**) 7
Scheme S2. Isolation scheme of scopularides C-G (**3**-**7**)...7
Scheme S3. Isolation scheme of scopularide H (**8**) 7

1 Experimental

1.1 Fungal isolation and DNA taxonomic analysis

Three fungi *Scopulariopsis sp.* CMB-F458, *Beauveria sp.* CMB-F585 and *Scopulariopsis sp.* CMB-F115 were isolated from the gastrointestinal tract of a specimen of *Mugil* mullet fish, on M1 agar plate in presence of 3.3% artificial sea salt (M1S) and incubated at 26.5 °C for 8 days.

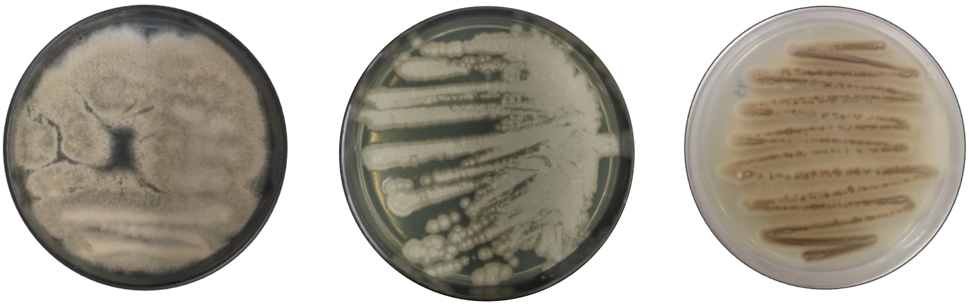

a) **b)** **c)**

Figure S1. CMB-F458 (**a**), CMB-F585 (**b**) and CMB-F115 (**c**) fungal strains cultured on M1S agar plates

1.1.1 CMB-F458

A BLAST analysis (NCBI database) on the resulting ITS gene sequence (GenBank accession no. MN080404) for CMB-F458 revealed 99% identity with the fungal strain *Scopulariopsis sp.*

ITS gene sequence

ATGAAAATAAAAGGTTCGACGGCTGGCCTTCGCCGGGACCCGATGCGAGATGTAGGATCTAC
TACGCAGGGGGCGCCGCGGCTGGACCGCCACTACATTTCGGGGACTGCGGGGGGGACGAGCC
CCACCCGTAGAGCCCCAACACCGGGCGACGGCAGGCCCCGTAGGGCTAGCCGCGCTCGAGGG
AAGAAATGACGCTCGGACAGGCATGCCCGGCAGATTGCTGCCGGGCGCAATGTGCGTTCAAA
GATTCGATGATTCACTGAATTCTGCAATTCACATTACTTATCGCATTTCGCTGCGTTCTTCA
TCGATGCCAGAACCAAGAGATCCGTTGTTAAAAGTTTTGACTTGTTTTTTGTTTTGAATCAG
AACGTGCAGTACGCTTTTTCAAATTTAGAGTTTGGCGCGGCCGGCGGGGACGGGGACGGGGA
CGGCGGCCCGGTGAGGGGCGCGCCGAACCCGAGCCCCGCCTCCCCGCCGAGGCAACGCGCGG
CAAGAGGTAAGGTTCACAATGGGTTTTGAAGGGTAACTTCGGTAATGATCCCTCCGCAGGTT
CACCTACGGA (**569 bp**)

BLAST search (1st closest match)

Scopulariopsis sp. isolate SFC101893 small subunit ribosomal RNA gene, partial sequence; internal transcribed spacer 1 and 5.8S ribosomal RNA gene, complete sequence; and internal transcribed spacer 2, partial sequence
Sequence ID: MF186101.1 **Length:** 578 **Number of Matches:** 1

Range 1: 1 to 568 GenBank Graphics ▼ Next Match ▲ Previous Match

Score	Expect	Identities	Gaps	Strand
1033 bits(559)	0.0	565/568(99%)	0/568(0%)	Plus/Minus

```
Query  1    ATGAAAATAAAAGGTTCGACGGCTGGCCTTCGCCGGGACCCGATGCGAGATGTAGGATCT  60
            ||||||||||||||||||||||||||||||||||||||||||||||||||||||||||||
Sbjct  568  ATGAAAATAAAAGGTTCGACGGCTGGCCTTCGCCGGGACCCGATGCGAGATGTAGGATCT  509

Query  61   ACTACGCAGGGGGCGCCGCGGCTGGACCGCCACTACATTTCGGGGACTGCggggggACG  120
            ||||||||||||||||||||||||||||||||||||||||||||||||||||||||||||
Sbjct  508  ACTACGCAGGGGGCGCCGCGGCTGGACCGCCACTACATTTCGGGGACTGCGGGGGGGACG  449

Query  121  AGCCCCACCCGTAGAGCCCCAACACCGGGCGACGGCAGGCCCCGTAGGGCTAGCCGCGCT  180
            ||||||||||||||||||||||||||||||||||||||||||||||||||||||||||||
Sbjct  448  AGCCCCACCCGTAGAGCCCCAACACCGGGCGACGGCAGGCCCCGTAGGGCTAGCCGCGCT  389

Query  181  CGAGGGAAGAAATGACGCTCGGACAGGCATGCCCGGCAGATTGCTGCCGGGCGCAATGTG  240
            ||||||||||||||||||||||||||||||||||||||||||||||||||||||||||||
Sbjct  388  CGAGGGAAGAAATGACGCTCGGACAGGCATGCCCGGCAGATTGCTGCCGGGCGCAATGTG  329

Query  241  CGTTCAAAGATTCGATGATTCACTGAATTCTGCAATTCACATTACTTATCGCATTTCGCT  300
            ||||||||||||||||||||||||||||||||||||||||||||||||||||||||||||
Sbjct  328  CGTTCAAAGATTCGATGATTCACTGAATTCTGCAATTCACATTACTTATCGCATTTCGCT  269

Query  301  GCGTTCTTCATCGATGCCAGAACCAAGAGATCCGTTGTTAAAAGTTTTGACTTGTTTTTT  360
            ||||||||||||||||||||||||||||||||||||||||||||||||||||||||||||
Sbjct  268  GCGTTCTTCATCGATGCCAGAACCAAGAGATCCGTTGTTAAAAGTTTTGACTTGTTTTTT  209

Query  361  GTTTTGAATCAGAACGTGCAGTACGCTTTTTCAAATTTAGAGTTTggcgcggccggcggg  420
            ||||||||||||||||||||||||||||||||||||||||||||||||||||||||||||
Sbjct  208  GTTTTGAATCAGAACGTGCAGTACGCTTTTTCAAATTTAGAGTTTGGCGCGGCCGGCGGG  149

Query  421  gacggggacggggacggcggcccggtgagggggcgcgCCGAACCCGAGCCCCGCCTCCCCG  480
            |||||| |||||||||||||||||||||||||||||||||||||||||||||||||||||
Sbjct  148  GACGGGAACGGGGACGGCGGCCCGGTGAGGGGCGCGCCGAACCCGAGCCCCGCCTCCCCG  89

Query  481  CCGAGGCAACGCGCGGCAAGAGGTAAGGTTCACAATGGGTTTTGAAGGGTAACTTCGGTA  540
            ||||||||||||||||||||||||||||||||||||||||||||||||||||||||||||
Sbjct  88   CCGAGGCAACGCGCGGCAAGAGGTAAGGTTCACAATGGGTTTTGAAGGGTAACTTCGGTA  29

Query  541  ATGATCCCTCCGCAGGTTCACCTACGGA  568
            ||||||||||||| |||||||| |||||
Sbjct  28   ATGATCCCTCCGCTGGTTCACCAACGGA  1
```

Scopulariopsis sp. isolate SFC101893 small subunit ribosomal RNA gene, partial sequence; internal transcribed spacer 1 and 5.8S ribosomal RNA gene, complete sequence; and internal transcribed spacer 2, partial sequence

GenBank: MF186101.1

FASTA Graphics PopSet

Go to:

```
LOCUS       MF186101                 578 bp    DNA     linear   PLN 01-JUN-2018
DEFINITION  Scopulariopsis sp. isolate SFC101893 small subunit ribosomal RNA
            gene, partial sequence; internal transcribed spacer 1 and 5.8S
            ribosomal RNA gene, complete sequence; and internal transcribed
            spacer 2, partial sequence.
ACCESSION   MF186101
VERSION     MF186101.1
KEYWORDS    .
SOURCE      Scopulariopsis sp.
  ORGANISM  Scopulariopsis sp.
            Eukaryota; Fungi; Dikarya; Ascomycota; Pezizomycotina;
            Sordariomycetes; Hypocreomycetidae; Microascales; Microascaceae;
            Scopulariopsis.
REFERENCE   1  (bases 1 to 578)
  AUTHORS   Park,M.S., Oh,S.-Y., Lee,S. and Lim,Y.W.
  TITLE     Marine fungi and enzyme activity associated with sailfin sandfish
            egg masses in Korea
  JOURNAL   Unpublished
REFERENCE   2  (bases 1 to 578)
  AUTHORS   Park,M.S., Oh,S.-Y., Lee,S. and Lim,Y.W.
  TITLE     Direct Submission
  JOURNAL   Submitted (29-MAY-2017) School of Biological Sciences, Seoul
            National University, 1 Gwanak-ro, Gwanak-gu, Seoul 08826, Republic
            of Korea
```

Figure S2. Blast search (closest match) for CMB-F458

1.1.2 CMB-F585

A BLAST analysis (NCBI database) on the resulting ITS gene sequence (GenBank accession no. MN080403) for CMB-F585 revealed 98% identity with the fungal strain *Beauveria sp.*

ITS gene sequence

CCGAGGTCACCTTGAGAAGTAGGGTGTTTCACGGCGTGGCCGCGCCGCCTTCCCGCCGCGAG
GTGAGTTATTACTACGCGGGGGAGGTCGCGACGTGACCGCCACTGTATTTCAGGGCCGGCCG
GCCTCTGTGGGCGCGGCCGATCCCCAACGCCAGGTCCCGCGGACGGGCCCTGAGGGTTGAAA
TGACGCTCGGACAGGCATGCCCGCCAGGATGCTGGCGGGCGCAATGTGCGTTCAAAGATTCG
ATGATTCACTGAATTCTGCAATTCACATTACTTATCGCATTTTGCTGCGTTCTTCATCGATG
CCAGAGCCAAGAGATCCGTTGCTGAAAGTTTTGATTTATTGTATATCAAACTCTGAAGAGTC
CACTATGGATAAAATGGTTTGGGTCCCTCGGCGGGCGCCCCGGGGGGTTCCCGCCGAAGCAA
CGGTGGTATGTTCACAAAGGTTTGGAGTTCGGAGACTCTGTAATGATCCCTCCGCAGGTTAC
CCTACGG (**504 bp**)

BLAST search (1st closest match)

Download GenBank Graphics ▼ Next ▲ Previous ▲ Descriptions

Beauveria felina isolate BfGZ5003 internal transcribed spacer 1, partial sequence; 5.8S ribosomal RNA gene and internal transcribed spacer 2, complete sequence; and large subunit ribosomal RNA gene, partial sequence
Sequence ID: MH483812.1 **Length:** 532 **Number of Matches:** 1

Related Information

Range 1: 6 to 516 GenBank Graphics ▼ Next Match ▲ Previous Match

Score	Expect	Identities	Gaps	Strand
891 bits(482)	0.0	502/511(98%)	4/511(0%)	Plus/Plus

```
Query  1    TCCAACCTTTGTGACATACCACCGTTGCTTCGGCGGGAACCCCCC--GGGGCGCCCGCCG  58
Sbjct  6    TCCAACCTTTGTGACATACCACCGTTGCTTCGGCGGGAACCCCCCCGGGGGCGCCCGCCG  65
Query  59   AGGGACCCAAACCATTTTATCCATAGTGGACTCTTCAGAGTTTGATATACAATAAATCAA  118
Sbjct  66   AGGGACCCAAACCATTTTATCCATAGTGGACTCTTCAGAGTTTGATACACAATCAATCAA  125
Query  119  AACTTTCAGCAACGGATCTCTTGGCTCTGGCATCGATGAAGAACGCAGCAAAATGCGATA  178
Sbjct  126  AACTTTCAGCAACGGATCTCTTGGCTCTGGCATCGATGAAGAACGCAGCAAAATGCGATA  185
Query  179  AGTAATGTGAATTGCAGAATTCAGTGAATCATCGAATCTTTGAACGCACATTGCGCCCGC  238
Sbjct  186  AGTAATGTGAATTGCAGAATTCAGTGAATCATCGAATCTTTGAACGCACATTGCGCCCGC  245
Query  239  CAGCATCCTGGCGGGCATGCCTGTCCGAGCGTCATTTCAA-CCCTCAGGGCCCGTCCGCG  297
Sbjct  246  CAGCATCCTGGCGGGCATGCCTGTCCGAGCGTCATTTCAACCCCTCGGGGCCCGTCCGCG  305
Query  298  GGACCTGGCGTTGGGGATCGGCCGCGCCCACAGAGGCCGGCCGGCCCTGAAATACAGTGG  357
Sbjct  306  GGACCTGGCGTTGGGGATCGGCCGCGCCCACAGAGGCCGGCCGGCCCTGAAATGCAGTGG  365
Query  358  CGGTCACGTCGCGACCTCCCCCGCGTAGTAATAACTCACCTCGCGGCGGGAAGGCGGCGC  417
Sbjct  366  CGGTCACGTCGCGACCTCCCCCGCGTAGTAATAACTCACCTCGCGGCGGGAAGGCGGCGC  425
Query  418  GGCCACGCCGTGAAACACCCTACTTCTCAAGGTTGACCTCGGATCAGGTAGGAATACCCG  477
Sbjct  426  GGCCACGCCGTGAAACACCCAACTTCTCAAGGTTGACCTCGGATCAGGTAGGAATACCCG  485
Query  478  CTGAACTTAAGCATATCAATAA-GCGGAGGA  507
Sbjct  486  CTGAACTTAAGCATATCAATAAAGCGGAGGA  516
```

Beauveria felina isolate BfGZ5003 internal transcribed spacer 1, partial sequence; 5.8S ribosomal RNA gene and internal transcribed spacer 2, complete sequence; and large subunit ribosomal RNA gene, partial sequence

GenBank: MH483812.1

FASTA Graphics PopSet

Go to:

```
LOCUS       MH483812                 532 bp    DNA     linear   PLN 10-JUL-2018
DEFINITION  Beauveria felina isolate BfGZ5003 internal transcribed spacer 1,
            partial sequence; 5.8S ribosomal RNA gene and internal transcribed
            spacer 2, complete sequence; and large subunit ribosomal RNA gene,
            partial sequence.
ACCESSION   MH483812
VERSION     MH483812.1
KEYWORDS    .
SOURCE      Beauveria felina
  ORGANISM  Beauveria felina
            Eukaryota; Fungi; Dikarya; Ascomycota; Pezizomycotina;
            Sordariomycetes; Hypocreomycetidae; Hypocreales; Cordycipitaceae;
            Beauveria.
REFERENCE   1  (bases 1 to 532)
  AUTHORS   Hu,Q.
  TITLE     Direct Submission
  JOURNAL   Submitted (13-JUN-2018) PESTICIDE SCIENCE, SOUTH CHINA AGRICULTURAL
            UNIVERSITY, WUSHAN 483, GUANGZHOU, GUANGDONG 510642, China
```

Figure S3. Blast search (closest match) for CMB-F585

1.1.3 CMB-F115

A BLAST analysis (NCBI database) on the resulting ITS gene sequence (GenBank accession no. MN080405) for CMB-F115 revealed 98% identity with the fungal strain *Scopulariopsis sp.*

ITS gene sequence

CTCCTGTGACCCTACCCCTCGCCGCGCGGTTGCCTCGGCGCGGGGGTCCGGGGCGGGGCTCA
GGCCCCTGCCCCCGGGTCCCCCGCCGGCCGCGCCCAACTCTTCCTTCTGCAAAGCGGACTGC
ATGTTCTGATTGCAACTTGAAAAACAAGTCAAAACTTTCAACAACGGATCTCTTGGTTCTGG
CATCGATGAAGAACGCAGCGAAATGCGATAAGTAATGTGAATTGCAGAATTCAGTGAATCAT
CGAATCTTTGAACGCACATTGCGCCCGGCAGTAATCTGCCGGGCATGCCTGTCCGAGCGTCA
TTTCTGCCCTCGAGCGCGGTCCCCGTACCCTCAGGGGCACGGGGCCGCCGCCCGGTGTTGGG
GCGCTGCGGGCCTAGAGCCCGGCAGGCCCCGAAATGAAGTGGCGGTCCCGCCGCGGCGCCCC
CTGCGTAGTAAGTCTCAGGACCTCGCATCGGGCCCCGGCGTCGGCCAGCCGTCGAACCTGTC
TCTCTGATGGTTTGACCTCGGATCAGGTAGGGTTACCCGCTGAACTTAAGCATATCAATAAG
CGGAGG (**565 bp**)

BLAST search (1st closest match)

Scopulariopsis sp. BMU01837 genomic DNA sequence contains 18S rRNA gene, ITS1, 5.8S rRNA gene, ITS2 and 28S rRNA gene, strain BMU01837

Sequence ID: LN850760.1 **Length:** 645 **Number of Matches:** 1

▷ See 1 more title(s)

Range 1: 76 to 644 GenBank Graphics ▼ Next Match ▲ Previous Match

Score	Expect	Identities	Gaps	Strand
990 bits(536)	0.0	559/569(98%)	5/569(0%)	Plus/Plus

```
Query  1    CTCCTGTG-ACCCTACCCCTCGCCGCGCGGTTGCCTcggcgcggggtccggggcggggc  59
Sbjct  76   CTCCTGTGAACCCTACCCCTCGCCGCGCGGTTGCCTCGGCGCGGGGTCCGGGGCGGGGC  135
Query  60   t-caggcccctgc-ccccgggtcccccgccggccgcgcccAACTCTTCCTTCTGCAAAGC  117
Sbjct  136  TTCAGGCCCCCGCTCCCCGGGTCCCCCGCCGGCCGCGCCCAACTCTTCCTTCTGCAAAGC  195
Query  118  GGACTGCATGTTCTGATTGCAACTTGAAAAACAAGTCAAAACTTTCAACAACGGATCTCT  177
Sbjct  196  GGACTGCACGTTCTGATTGCAACTTGAAAAACAAGTCAAAACTTTCAACAACGGATCTCT  255
Query  178  TGGTTCTGGCATCGATGAAGAACGCAGCGAAATGCGATAAGTAATGTGAATTGCAGAATT  237
Sbjct  256  TGGTTCTGGCATCGATGAAGAACGCAGCGAAATGCGATAAGTAATGTGAATTGCAGAATT  315
Query  238  CAGTGAATCATCGAATCTTTGAACGCACATTGCGCCCGGCAGTAATCTGCCGGGCATGCC  297
Sbjct  316  CAGTGAATCATCGAATCTTTGAACGCACATTGCGCCCGGCAGCAATCTGCCGGGCATGCC  375
Query  298  TGTCCGAGCGTCATTTCTGCCCTCGAGCGCGGTCCCCGTACC--CTCAGGGGCACGGGGC  355
Sbjct  376  TGTCCGAGCGTCATTTCTGCCCTCGAGCGCGGTTCCCGTACCCTCTCAGGGGCACGGGGC  435
Query  356  CGCCGCCCGGTGTTGGGGCGCTGCGGGCCTAGAGCCCGGCAGGCCCCGAAATGAAGTGGC  415
Sbjct  436  CGCCGCCCGGTGTTGGGGCGCTGCGGGCCTAGAGCCCCGCAGGCCCCGAAATGAAGTGGC  495
Query  416  GGTCCCGCCGCGGCGCCCCCTGCGTAGTAAGTCTCAGGACCTCGCATCGGGCCCCGGCGT  475
Sbjct  496  GGTCCCGCCGCGGCGCCCCCTGCGTAGTAAGTCTCAGGACCTCGCATCGGGCCCCGGCGT  555
Query  476  CGGCCAGCCGTCGAACCTGTCTCTCTGATGGTTTGACCTCGGATCAGGTAGGGTTACCCG  535
Sbjct  556  CGGCCAGCCGTCGAACCTGTCTCTCTGATGGTTTGACCTCGGATCAGGTAGGGTTACCCG  615
Query  536  CTGAACTTAAGCATATCAATAAGCGGAGG  564
Sbjct  616  CTGAACTTAAGCATATCAATAAGCGGAGG  644
```

Scopulariopsis sp. BMU01837 genomic DNA sequence contains 18S rRNA gene, ITS1, 5.8S rRNA gene, ITS2 and 28S rRNA gene, strain BMU01837

GenBank: LN850760.1

FASTA Graphics

Go to:

```
LOCUS       LN850760                 645 bp    DNA     linear   PLN 13-APR-2016
DEFINITION  Scopulariopsis sp. BMU01837 genomic DNA sequence contains 18S rRNA
            gene, ITS1, 5.8S rRNA gene, ITS2 and 28S rRNA gene, strain
            BMU01837.
ACCESSION   LN850760
VERSION     LN850760.1
KEYWORDS    .
SOURCE      Microascus chinensis
  ORGANISM  Microascus chinensis
            Eukaryota; Fungi; Dikarya; Ascomycota; Pezizomycotina;
            Sordariomycetes; Hypocreomycetidae; Microascales; Microascaceae;
            Microascus.
REFERENCE   1
  AUTHORS   Jagielski,T., Sandoval-Denis,M., Yu,J., Yao,L., Bakula,Z.,
            Kalita,J., Skora,M., Krzysciak,P., de Hoog,G.S., Guarro,J. and
            Gene,J.
  TITLE     Molecular taxonomy of scopulariopsis-like fungi with description of
            new clinical and environmental species
  JOURNAL   Fungal Biol 120 (4), 586-602 (2016)
   PUBMED   27020159
REFERENCE   2  (bases 1 to 645)
  AUTHORS   Sandoval-Denis,M.
  TITLE     Direct Submission
  JOURNAL   Submitted (07-MAY-2015) Microbiology Unit, UNIVERSITAT ROVIRA I
            VIRGILI, Microbiology Unit, Universitat Rovira i Virgili, Sant
            Llorent 21, Reus., 43201, 43201, SPAIN
```

Figure S4. Blast search (closest match) for CMB-F115

1.2 Phylogenetic tree

Phylogenetic tree obtained by PhyML Maximum Likelihood analysis was constructed using the top similar 18S rRNA sequences displayed after BLAST on Refseq RNA NCBI database using CMB-F458, CMB-F585 and CMB-115 18S rRNA as queries. The JC69 model was used to infer phylogeny sequences [1]. Sequences alignments were produced with the MUSCLE program [2]. Phylogenetic tree was constructed with UGENE software using the aforementioned models and visualized using Ugene's tree view [3].

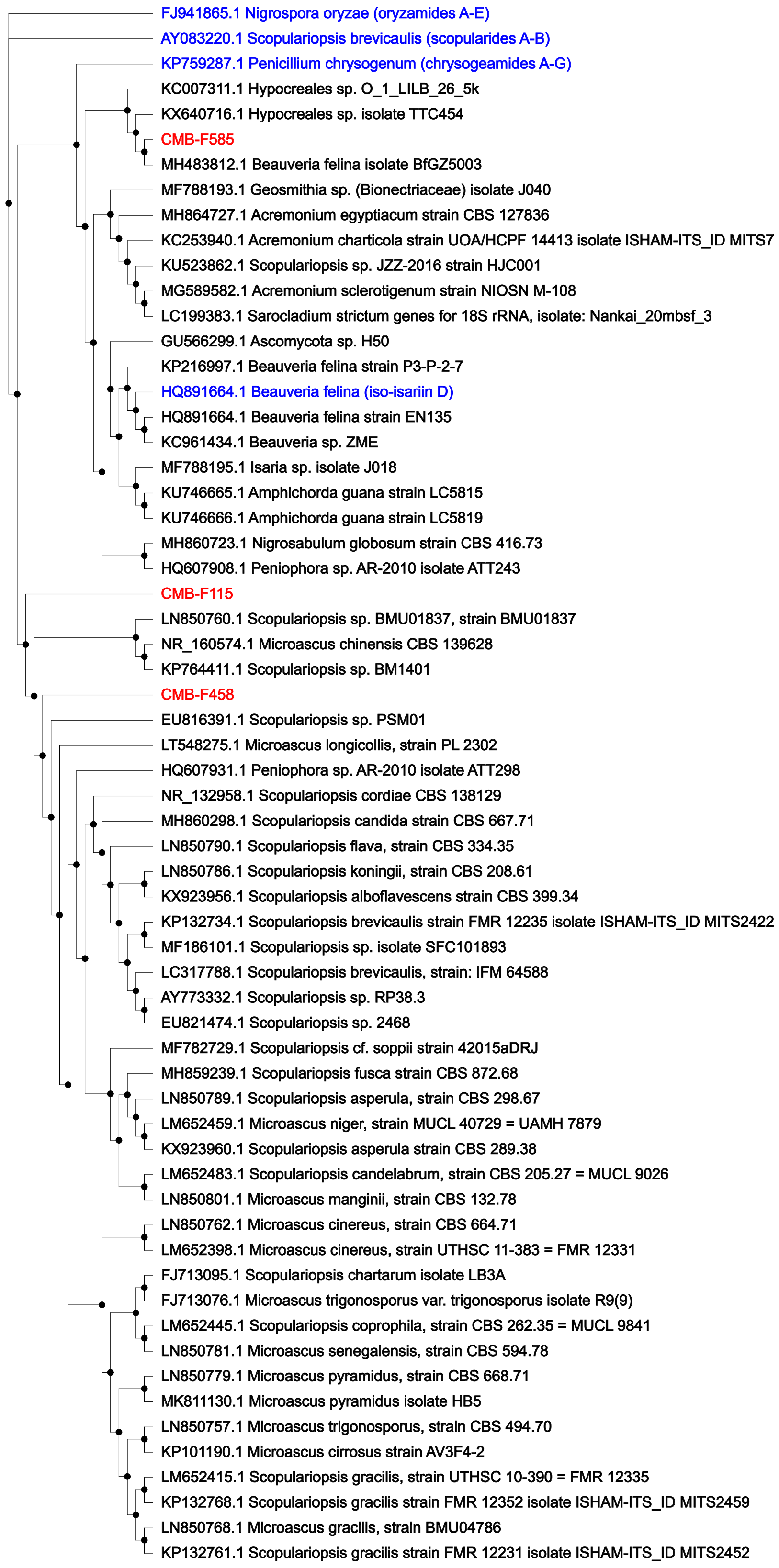

Figure S5. Phylogenetic tree by PhyML Maximum Likehood analysis of 18s rRNA sequences showing the relationship of CMB-F458, CMB-F585 and CMB-F115 among selected reference strains (RefSeq GenBank) with their accession numbers

1.3 Cultivation of the fungal strains, production and isolation of compounds 1-8

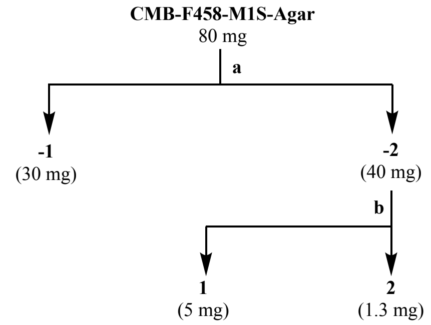

Scheme S1. Isolation scheme of scopularides A (**1**) and B (**2**): a) Defatting of crude extract with hexane (-1) to yield defatted crude extract (-2); b) Preparative HPLC purification.

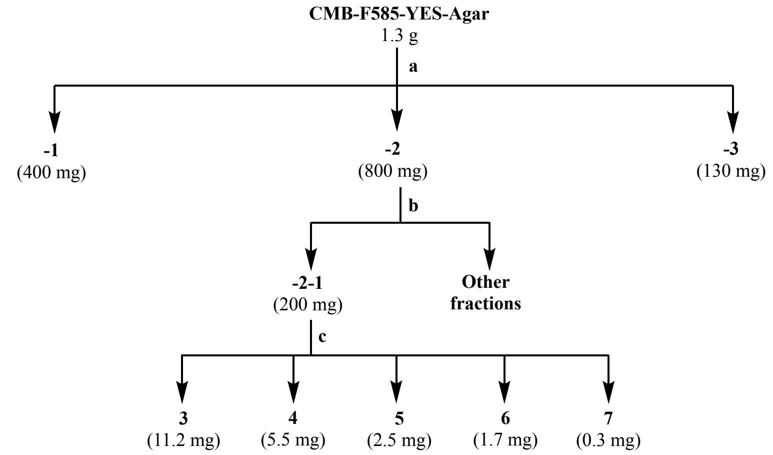

Scheme S2. Isolation scheme of scopularides C-G (**3-7**): a) trituration of the crude extract using hexane (-1), DCM (-2) and MeOH (-3); b) column chromatography: Sephadex® LH-20 (2.5 cm × 70 cm) for 700 mg, isocratic elution with MeOH (100 %); c) semi-preparative HPLC purification for 60 mg.

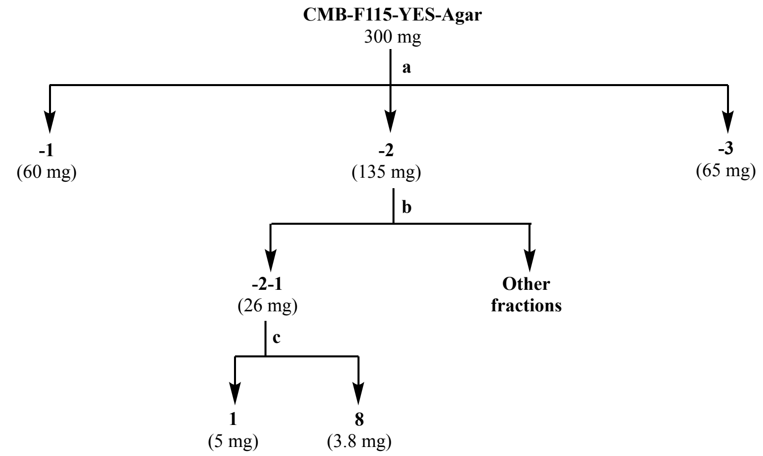

Scheme S3. Isolation scheme of scopularide H (**8**): a) trituration of crude extract between hexane (-1), DCM (-2) and MeOH (-3); b) column chromatography: Sephadex® LH-20 (1.5 cm × 22 cm), isocratic elution with MeOH (100 %); c) semi-preparative HPLC purification for 20 mg.

2 Spectroscopic characterization of metabolites 1-6 and 8

2.1 Scopularide A (1)

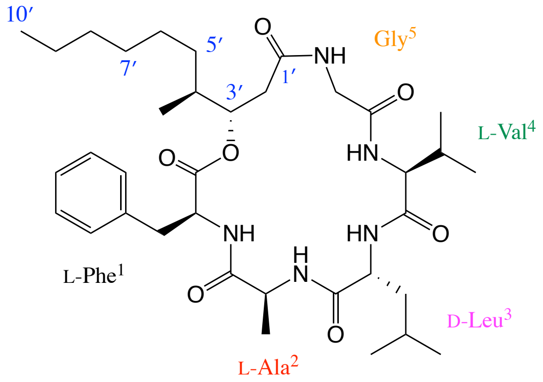

Table S1. NMR (600 MHz, MeOH-d_4) data for scopularide A (1)

Position		Scopularide A (1)		Reported scopularide A [7]	
		δ_H, mult (*J* in Hz)	δ_C	δ_H, mult (*J* in Hz)	δ_C
L-Phe[1]	1	---	173.8	---	173.6
	2	4.78, dd (*8.2, 7.7*)	55.4	4.79, m	55.3
	3	a 3.18, dd (*13.5, 7.7*)	39.5	3.18, dd (*13.5, 7.7*)	39.4
		b 3.03, dd (*13.5, 8.2*)		3.03, dd (*13.5, 8.2*)	
	4	---	137.9	---	137.8
	5/9	7.24, m	130.5	7.24, m	130.3
	6/8	7.28, m	129.8	7.28, m	129.6
	7	7.21, m	128.2	7.21, m	128.0
L-Ala[2]	1	---	174.2	---	174.1
	2	4.29, dq (*7.3, 7.3*)	50.4	4.29, q (*7.3*)	50.3
	3	1.28[b], d (*7.3*)	17.7	1.27, d (*7.3*)	17.6
D-Leu[3]	1	---	175.3	---	175.1
	2	4.19, dd (*8.0, 7.3*)	54.8	4.19, m	54.7
	3	1.58, dd (*7.3, 6.9*)	40.4	1.58, m	40.3
	4	1.68, m	26.0	1.68, m	26.0
	5	1.00, d (*6.5*)	23.2	1.00, d (*6.6*)	23.1
	6	0.95[a], d (*6.5*)	22.5	0.95, d (*6.6*)	22.3
L-Val[4]	1	---	173.6	---	173.4
	2	4.20, d (*6.0*)	60.0	4.20, m	59.9
	3	2.18, m	31.2	2.19, m	31.0
	4	0.98, d (*6.4*)	19.1	0.98, d (*6.4*)	19.0
	5	0.97, d (*6.4*)	19.8	0.97, d (*6.4*)	19.7
Gly[5]	1	---	172.0	---	171.9
	2a	a 4.24, d (*16.8*)	44.1	4.24, d (*16.8*)	44.0
	b	b 3.52, d (*16.8*)		3.53, d (*16.8*)	
HMDA*	1′	---	174.7	---	174.5
	2′	2.41, m	41.0	2.42, m	40.9
	3′	4.75, m	78.7	4.77, m	78.6
	4′	1.53, m	39.1	1.54, m	39.0
	5′	a 1.21, m	33.7	1.22, m	33.6
		b 0.96[a], m		0.95, m	
	6′	1.28[b], m	30.7	1.30, m	30.6
	7′	a 1.26[c], m	28.4	1.29, m	28.2
		b 1.16, m			
	8′	1.26[c], m	33.1	1.26, m	33.0
	9′	1.32, m	23.9	1.31, m	23.7
	10′	0.90, t (*7.2*)	14.6	0.91, t (*7.2*)	14.42
	4′-Me	0.83, d (*6.9*)	14.5	0.83, d (*6.9*)	14.41

*HMDA: *3S*, *4S*-3-Hydroxy-4-methyldecanoic acid

(a – c) overlapping resonances within the same letter

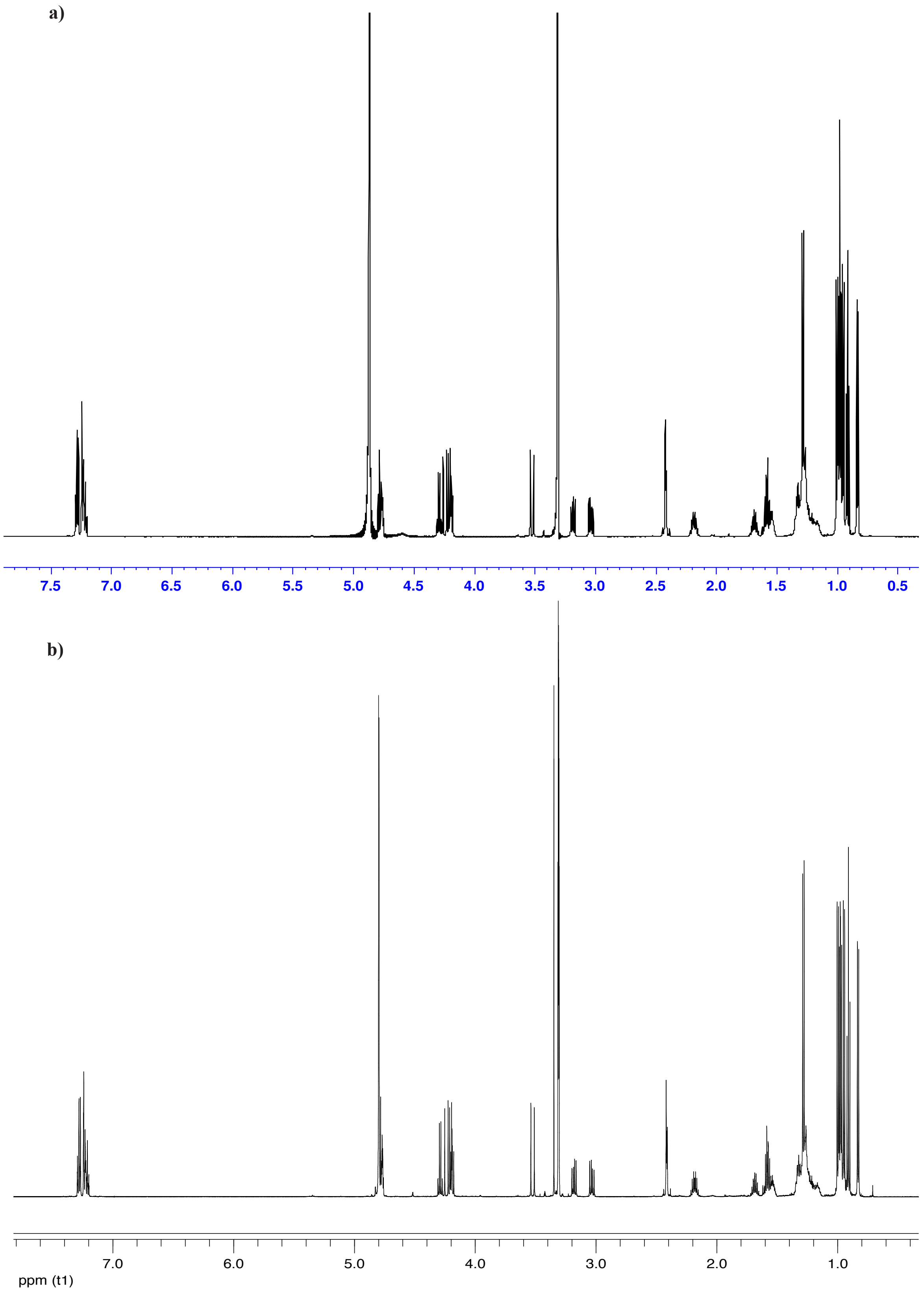

Figure S6. ^{1}H NMR (MeOH-d_4) spectra for a) scopularide A (**1**) (600 MHz) compared to b) reported scopularide A [7]

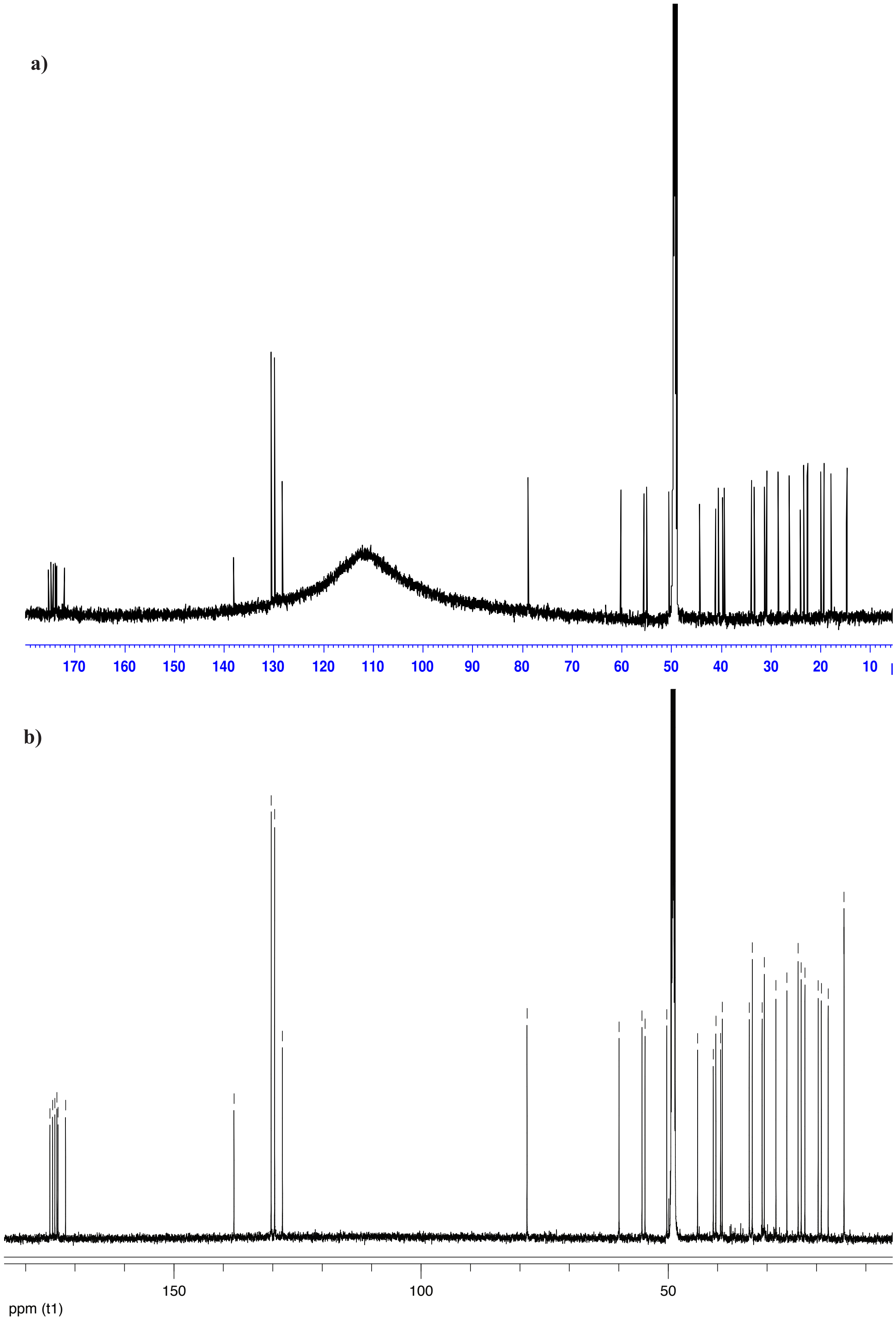

Figure S7. ^{13}C NMR (MeOH-d_4) spectra for a) scopularide A (**1**) (150 MHz) compared to b) reported scopularide A [7]

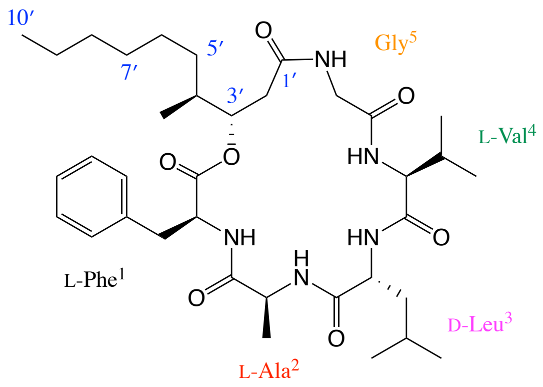

Table S2. NMR (600 MHz, DMSO-d_6) data for scopularide A (1)

Position		Scopularide A (1)		reported arenamide A**[8]	
		δ_H, mult (*J* in Hz)	δ_C	δ_H, mult (*J* in Hz)	δ_C
L-Phe[1]	1	---	170.8	---	169.8
	2	4.34, q (14.7, 7.1)	54.5	4.34, q (14.1, 7.2)	55.6
	3	a 2.97, dd (14.1, 7.1)	36.7	2.97, dd (14.1, 7.2)	38.1
		b ND		2.95, dd (14.1, 8.2)	
	4	---	137.2	---	137.2
	5/9	7.26, m	129.0	7.26, m	129.1
	6/8	7.26, m	128.3	7.26, m	128.2
	7	7.20, m	126.5	7.20, m	126.5
	NH	8.03, d (7.1)	---	8.03, d (6.4)	---
L-Ala[2]	1	---	171.8	---	168.9
	2	4.19, dq (7.9, 7.2)	47.6	4.19, p (14.6, 7.2)	49.0
	3	1.15, d (7.2)	17.7	1.15, d (7.2)	17.8
	NH	7.83, d (7.9)	---	7.83, d (7.6)	---
D-Leu[3]	1	---	171.0	---	170.8
	2	4.03, dd (10.6, 6.0)	52.0	4.03, m	52.6
	3	1.46, m	38.7	1.5, m	39.7
	4	1.63, m	24.1	1.63, m	25.1
	5	0.89, d (6.1)	22.9	0.89, d (6.3)	22.9
	6	0.81, d (6.1)	21.1	0.84, d (6.3)	21.4
	NH	8.63, d (6.0)	---	8.63, d (7.2)	---
L-Val[4]	1	---	171.7	---	171.0
	2	4.10, dd (8.8, 7.9)	58.3	4.10, dd (8.5, 7.9)	59.2
	3	1.87, m	29.8	1.87, m	30.7
	4	0.88, d (7.1)	18.9	0.88, d (6.8)	18.9
	5	0.84, d (7.1)	18.7	0.86, d (6.8)	18.9
	NH	7.88, d (7.9)	---	7.88, d (8.1)	---
Gly[5]	1	---	168.9	---	171.7
	2	a 4.07, d (16.6)	42.3	4.05, dd (16.4, 5.4)	43.5
		b 3.41, dd (16.6, 4.0)		3.42, dd (16.4, 4.3)	
	NH	7.93, dd (6.3, 4.1)	---	7.93, dd (4.3, 5.4)	---
HMDA*	1′	---	169.8	---	171.8
	2′	a 2.47, dd (14.6, 9.3)	37.1	2.46, d (9.3)	38.4
		b 2.24, dd (14.6, 1.8)		2.24, d (14.4)	
	3′	4.90, ddd (9.1, 4.0, 1.8)	75.0	4.9, dd (9.3, 2.1)	75.8
	4′	1.50, m	36.1	1.51, m	37.1
	5′	1.20, m	31.4	1.16, m	32.5
	6′	a 1.18[a], m	26.5	1.18, m	26.9
		b 1.09, m			
	7′	1.18[a], m	28.9	1.14, m	30.8
	8′	1.20, m	31.2	1.16, m	32.1
	9′	1.25, m	22.1	1.26, m	23.1
	10′	0.85, t (7.0)	14.0	0.83, t (6.7)	13.9
	4′-Me	0.67, d (6.8)	14.5	0.67, d (6.8)	14.5

*HMDA: *3S*, *4S*-3-Hydroxy-4-methyldecanoic acid, ** NMR data (500 MHz, DMSO-d_6)
ND signal not detected. (a) overlapping resonances

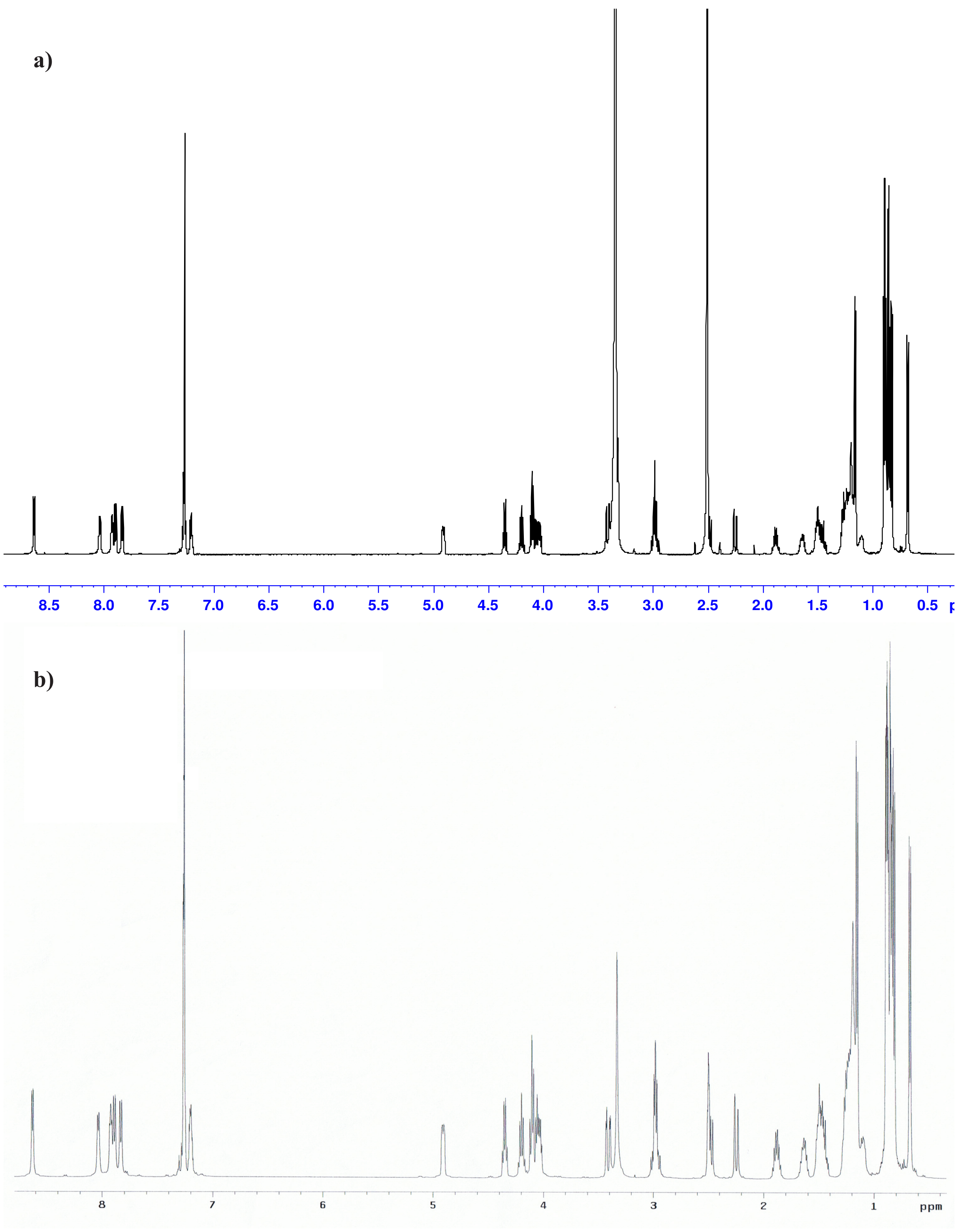

Figure S8. ^{1}H NMR (DMSO-d_6) spectra for scopularide A (**1**) compared to b) reported arenamide A [8]

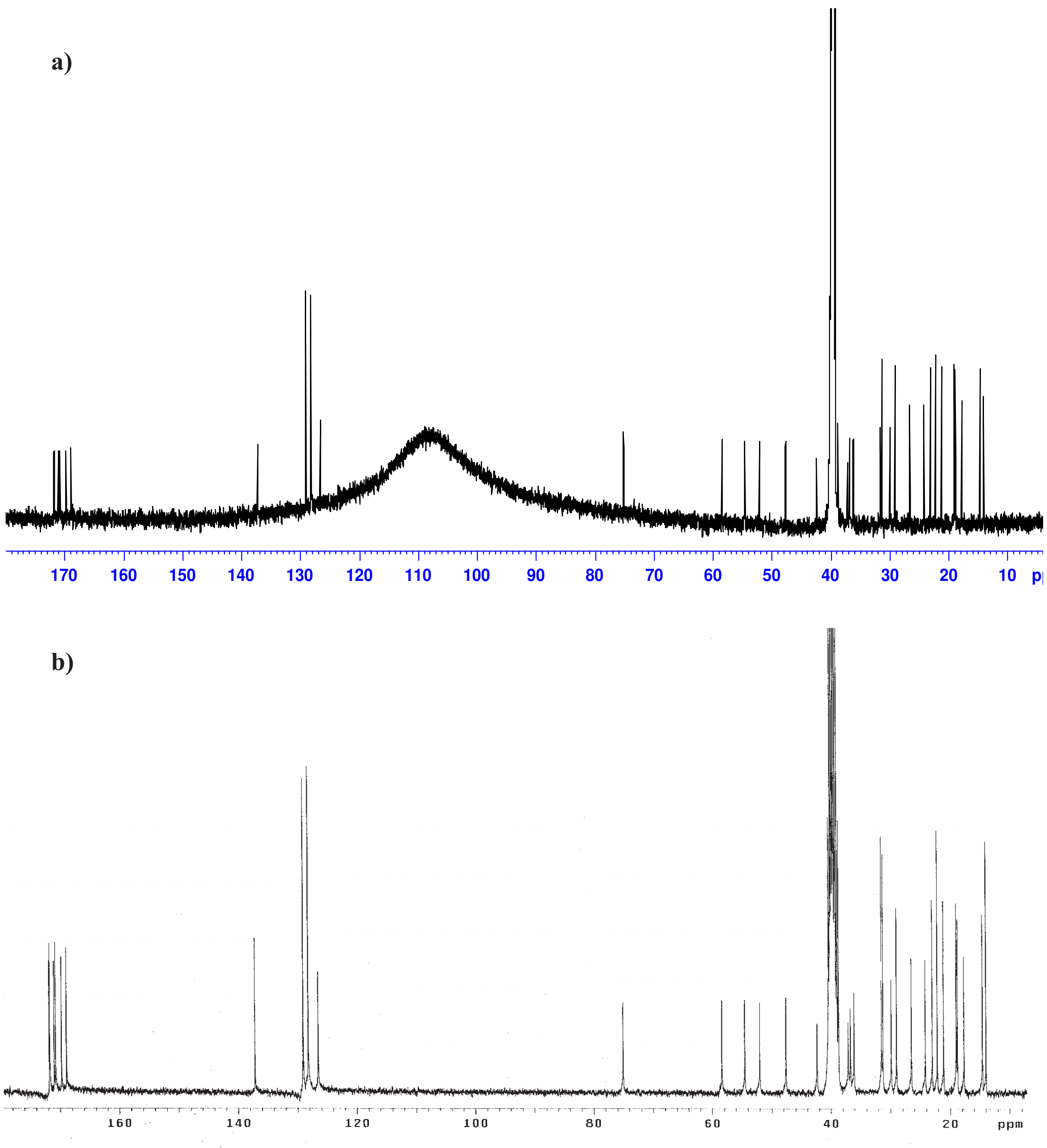

Figure S9. ^{13}C NMR (DMSO-d_6) spectra for scopularide A (**1**) compared to b) reported arenamide A [8]

2.2 Scopularide B (2)

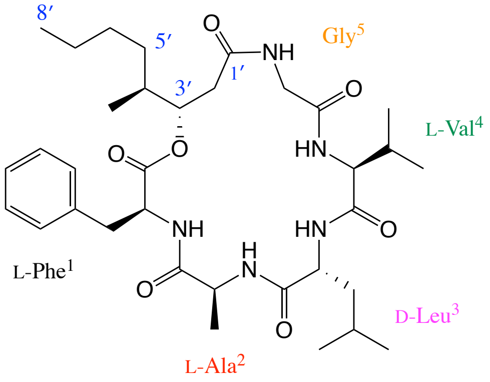

Table S3. NMR (600 MHz, MeOH-d_4) data for scopularide B (2)

Position		Scopularide B (2)		reported scopularide B[7]	
		δ_H, mult (*J* in Hz)	δ_C	δ_H, mult (*J* in Hz)	δ_C
**L-Phe¹**	**1**	---	173.8	---	173.6
	**2**	4.78, dd (*16.6, 7.8*)	55.4	4.78, m	55.3
	**3**	a 3.18, dd (*13.5, 7.8*)	39.5	3.18, dd (*13.5, 7.5*)	39.4
		b 3.03, dd (*13.5, 7.8*)		3.03, dd (*13.5, 8.2*)	
	**4**	---	137.9	---	137.8
	**5/9**	7.23, m	130.5	7.24, m	130.3
	**6/8**	7.28, m	129.8	7.28, m	129.6
	**7**	7.20, m	128.2	7.21, m	128.0
**L-Ala²**	**1**	---	174.2	---	174.1
	**2**	4.29, dq (*7.1, 7.1*)	50.4	4.29, q (*7.3*)	50.3
	**3**	1.28, d (*7.1*)	17.7	1.28, d (*7.3*)	17.6
**D-Leu³**	**1**	---	175.3	---	175.1
	**2**	4.19, m	54.8	4.19, m	54.7
	**3**	1.58, m	40.4	1.58, m	40.4
	**4**	1.68, m	26.0	1.69, m	26.0
	**5**	1.00, d (*6.5*)	23.2	0.99, d (*6.6*)	23.1
	**6**	0.95, d (*6.5*)	22.5	0.95, d (*6.6*)	22.3
**L-Val⁴**	**1**	---	173.6	---	173.4
	**2**	4.20, m	60.0	4.20, m	59.9
	**3**	2.18, m	31.2	2.18, m	31.0
	**4**	0.98, d (*6.5*)	19.1	0.98, d (*6.7*)	19.0
	**5**	0.97, d (*6.5*)	19.8	0.97, d (*6.7*)	19.7
**Gly⁵**	**1**	---	172.0	---	171.9
	**2**	a 4.24, d (*17.0*)	44.1	4.24, d (*16.8*)	44.0
		b 3.52, d (*17.0*)		3.53, d (*16.8*)	
**HMOA***	**1′**	---	174.7	---	174.5
	**2′**	2.41, m	41.0	2.41, m	40.9
	**3′**	4.75, m	78.7	4.77, m	78.6
	**4′**	1.53, m	39.1	1.54, m	39.0
	**5′**	a 1.22, m	33.4	1.22, m	33.3
		b 0.96, m		0.96, m	
	**6′**	a 1.29, m	30.6	1.32, m	30.5
		b 1.15, m		1.32, m	
	**7′**	1.27, m	24.0	1.16, m	23.8
	**8′**	0.90, t (*7.2*)	14.5	0.90, t (*7.2*)	14.38
	**4′-Me**	0.83, d (*6.5*)	14.5	0.83, d (*6.9*)	14.41

* HMOA: *3S*, *4S*-3-Hydroxy-4-methyloctanoic acid

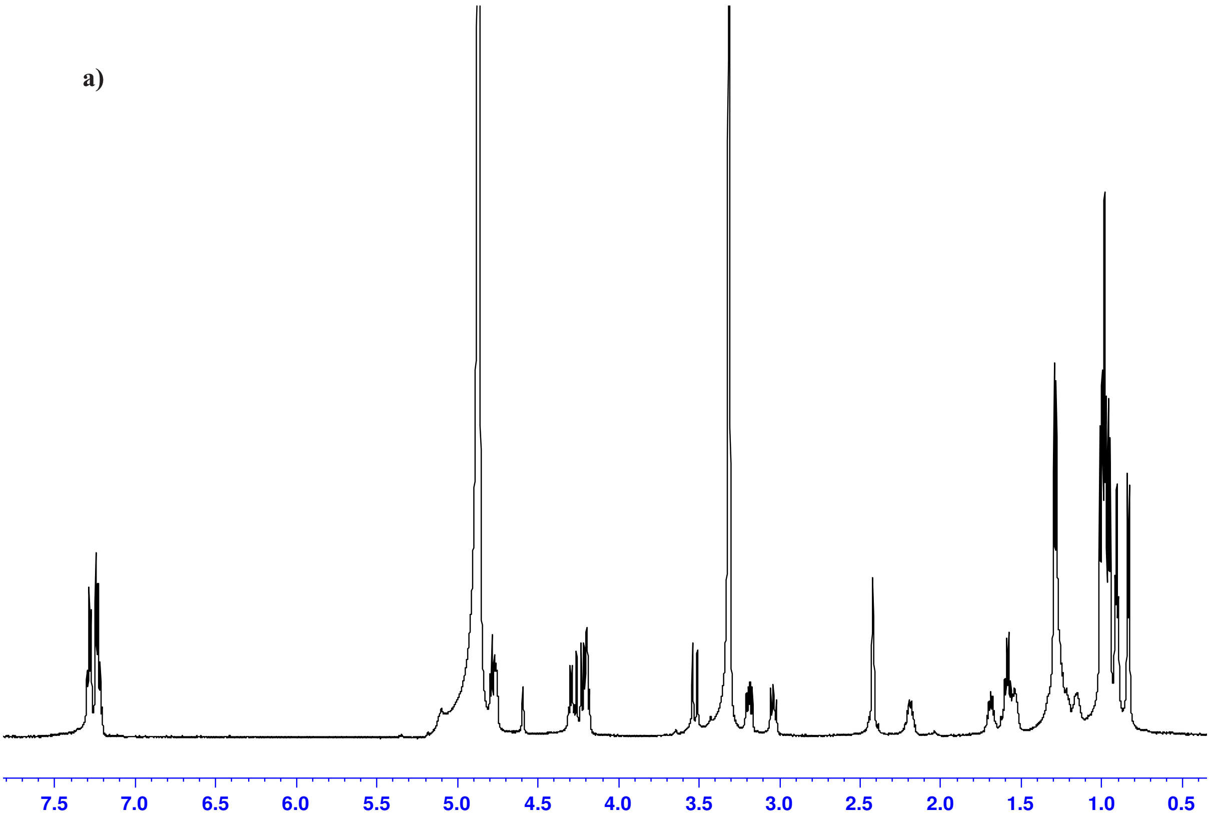

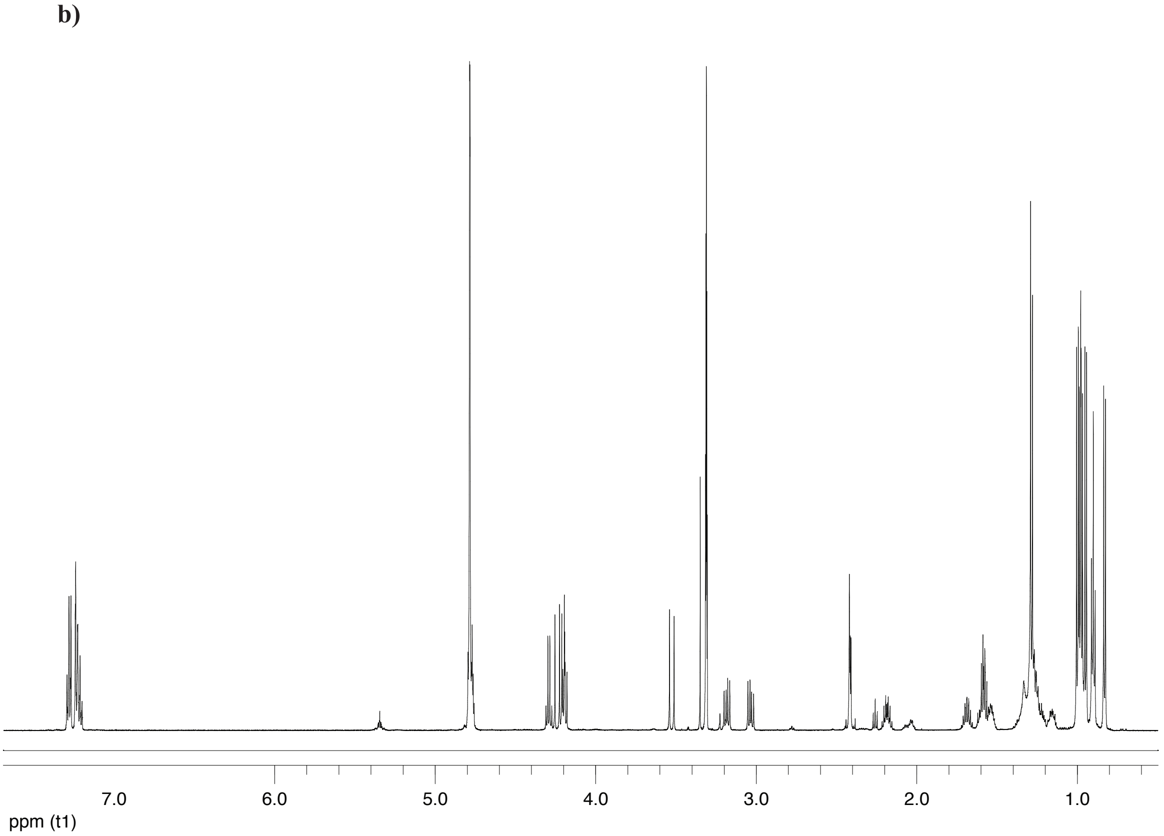

Figure S10. ^{1}H NMR (MeOH-d_4) spectra for scopularide B (**2**) (600 MHz) compared to b) reported scopularide B [7]

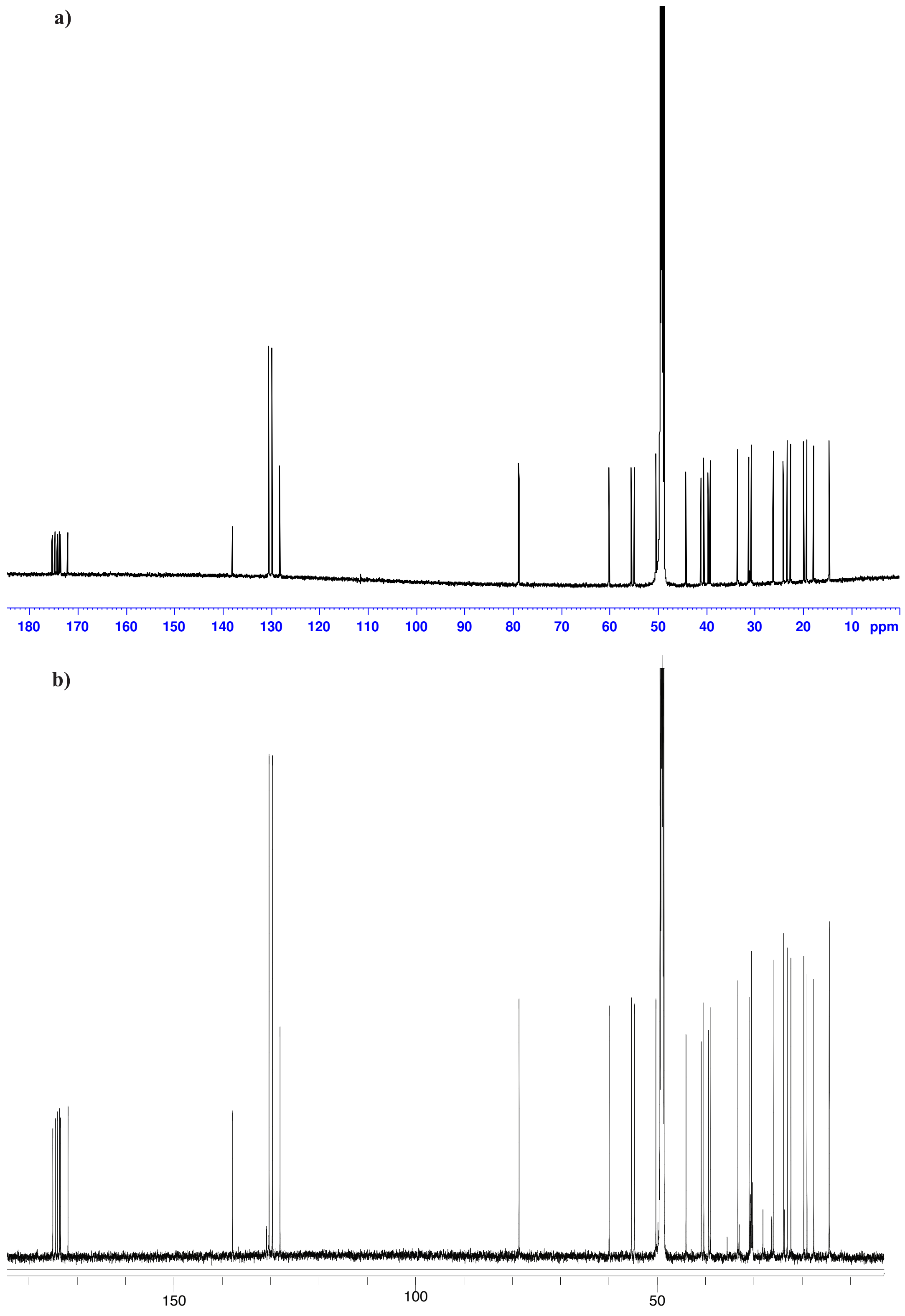

Figure S11. ^{13}C NMR (MeOH-d_4) spectra for scopularide B (**2**) (150 MHz) compared to b) reported scopularide B [7]

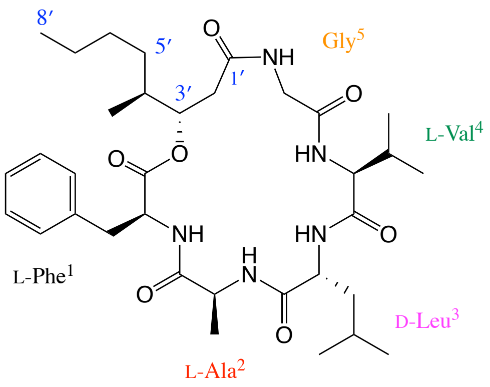

Table S4. NMR (600 MHz, DMSO-d_6) data for scopularide B (2)

Position		Scopularide B (2)		reported arenamide B**[8]	
		δ_H, mult (*J* in Hz)	δ_C	δ_H, mult (*J* in Hz)	δ_C
**L-Phe[1]**	**1**	---	170.8	---	169.8
	**2**	4.34, q (*14.6, 7.3)*	54.5	4.34, q (*14.1, 7.1*)	55.6
	**3**	a 2.98, dd (*14.6, 7.3*)	36.7	2.97, dd (*14.1, 7.1*)	38.1
		b 2.96, dd (*14.6, 8.0*)		2.95, dd (*14.1, 8.2*)	
	**4**	---	137.2	---	137.2
	**5/9**	7.26, m	129.0	7.26, m	129.1
	**6/8**	7.26, m	128.3	7.26, m	128.2
	**7**	7.20, m	126.5	7.20, m	126.5
	**NH**	8.03, d (*6.7*)		8.03, d (*6.7*)	
**L-Ala[2]**	**1**	---	171.8	---	168.9
	**2**	4.19, dq (*7.9, 7.1*)	47.6	4.19, p (*14.6, 7.2*)	49.0
	**3**	1.15, d (*7.1*)	17.6	1.15, d (*7.2*)	19.0
	**NH**	7.82, d (*7.9*)		7.83, d (*8.0*)	
**D-Leu[3]**	**1**	---	171.0	---	170.8
	**2**	4.03, dd (*10.8, 5.9*)	52.0	4.03, m	52.6
	**3**	1.46, m	38.7	1.50, m	39.7
	**4**	1.63, m	24.1	1.63, m	25.1
	**5**	0.88[b], d (*6.0*)	22.9	0.89, d (*6.3*)	22.9
	**6**	0.82, d (*6.4*)	21.0	0.84, d (*6.3*)	21.4
	**NH**	8.63, d (*5.9*)		8.63, d (*7.2*)	
**L-Val[4]**	**1**	---	171.7	---	171.0
	**2**	4.09, dd (*8.6, 8.2*)	58.3	4.10, t (*8.5*)	59.2
	**3**	1.87, m	29.8	1.87, m	30.7
	**4**	0.87[b], d (*6.8*)	18.9	0.88, d (*6.8*)	18.9
	**5**	0.84[c], d (*6.8*)	18.7	0.86, d (*6.8*)	18.7
	**NH**	7.89, d (*8.2*)		7.88, d (*8.1*)	
**Gly[5]**	**1**	---	168.9	---	171.7
	**2**	a 4.07, dd (*16.8, 5.8*)	42.3	4.05, dd (*16.4, 5.3*)	43.5
		b 3.41, dd (*16.8, 4.1*)		3.42, dd (*16.4, 2.4*)	
	**NH**	7.92, dd (*5.8, 4.1*)		7.93, t (*5.3, 2.4*)	
**HMOA***	**1′**	---	169.8	---	171.8
	**2′**	a 2.47, d (*9.3*)	37.1	2.46, d (*9.3*)	38.4
		b 2.24 d (*14.5*)		2.25, d (*14.5*)	
	**3′**	4.90, ddd (*8.8, 3.7, 1.4*)	75.1	4.90, dd (*9.3, 2.0*)	75.8
	**4′**	1.49, m	36.0	1.51, m	37.1
	**5′**	a 1.18[a], m	31.2	1.15, m	32.5
		b 0.87[b], m			
	**6′**	a 1.18[a], m	28.7	1.14, m	30.8
		b 1.08, m			
	**7′**	1.19, m	22.3	1.26, m	23.1
	**8′**	0.84[c], t (*6.9*)	13.9	0.83, t (*6.7*)	13.9
	**4′-Me**	0.67, d (*6.8*)	14.6	0.67, d (*6.8*)	14.5

* HMOA: *3S*, *4S*-3-Hydroxy-4-methyloctanoic acid, ** NMR data (500 MHz, DMSO-d_6)
(a-b) overlapping resonances within the same letter

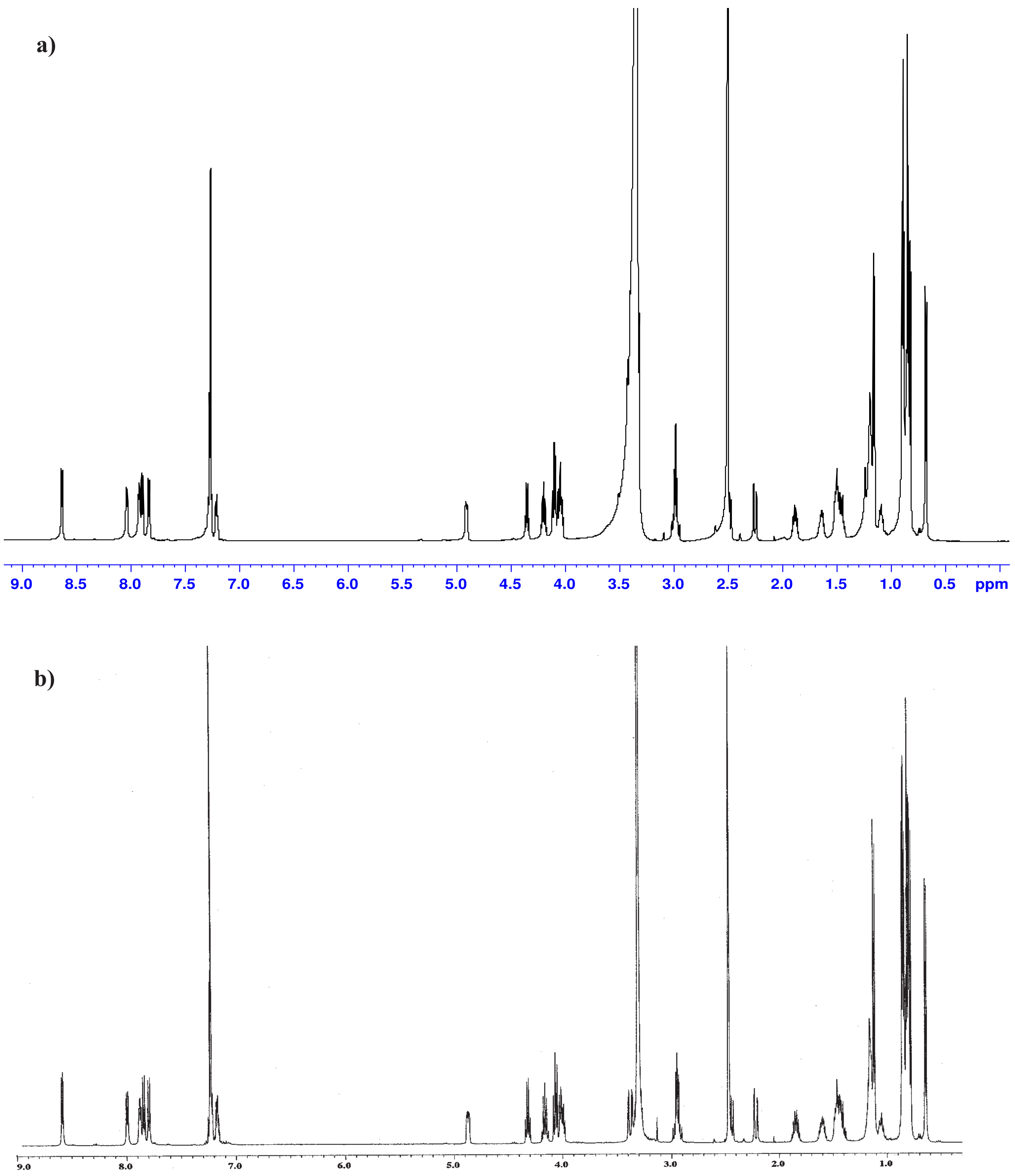

Figure S12. ^{1}H NMR (DMSO-d_6) spectrum for scopularide B (**2**) (600 MHz) compared to b) reported arenamide B [8]

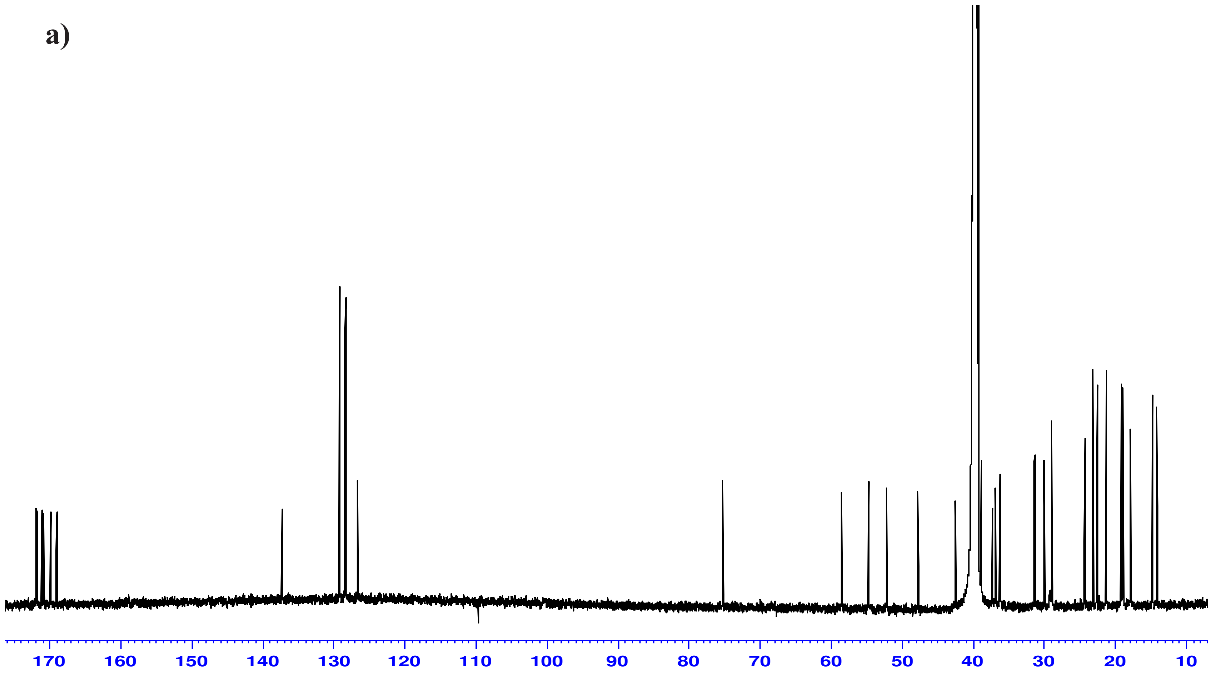

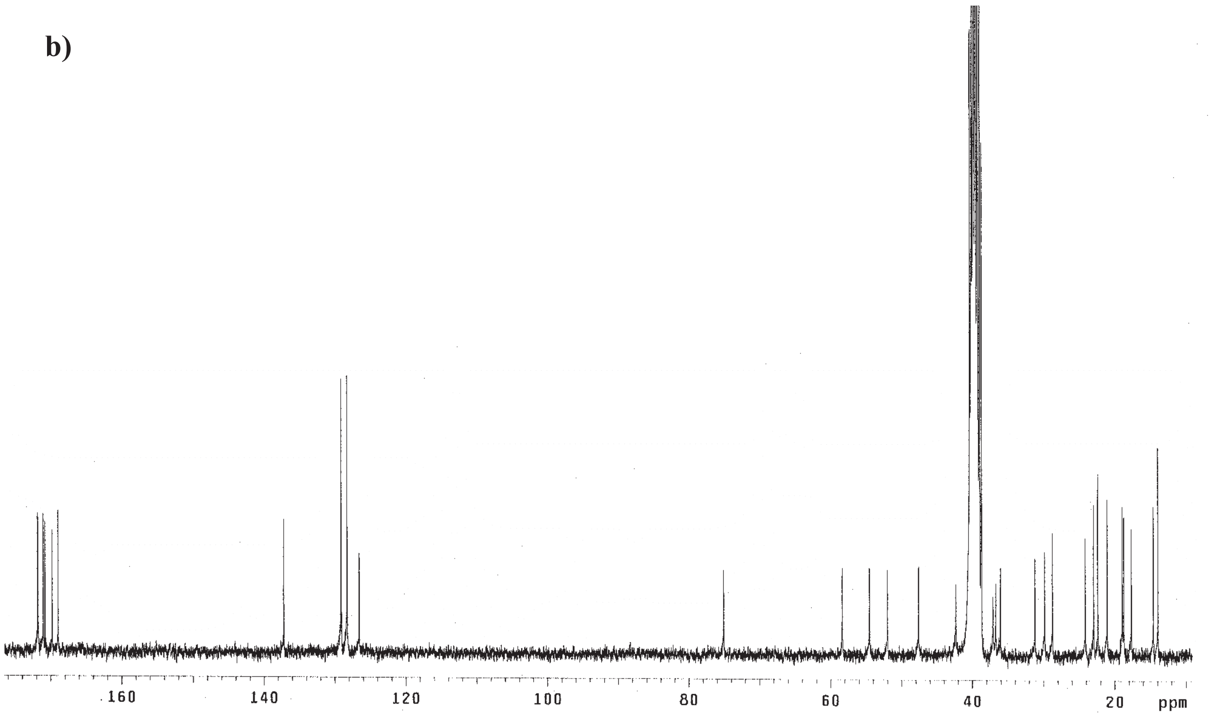

Figure S13. ^{13}C NMR (DMSO-d_6) spectra for scopularide B (**2**) (150 MHz) compared to b) reported arenamide B [8]

2.3 Scopularide C (3)

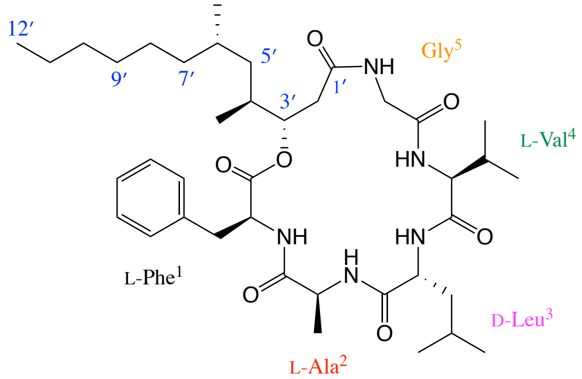

Table S5. 1D and 2D **NMR (600 MHz, DMSO-d_6) data for scopularide C (3)**

**Position**		**δ$_H$, mult (*J* in Hz)**	**δ$_C$**	**COSY**	**^{1}H-^{13}C HMBC**
**L-Phe^1**	**1**	---	170.8	---	---
	**2**	4.38, q (*7.1*)	54.2	3, Phe^1-NH	1, 3, 4
	**3**	a 3.01, dd (*13.9, 6.1*)	36.7	2	1, 2, 5/9
		b 2.93, d (*13.9, 8.7*)		2	1, 2, 5/9
	**4**	---	137.2	---	---
	**5/9**	7.25, m	129.0		3, 5/9, 7
	**6/8**	7.28, m	128.2	7	4, 6/8
	**7**	7.20, m	126.5	6/8	5/9
	**NH**	7.96, d (*7.1*)	---	2	2, 3, Ala^2-1
**L-Ala^2**	**1**	---	171.7[d]	---	---
	**2**	4.16, dq (*7.4, 7.1*)	47.6	3, Ala^2-NH	1, 3
	**3**	1.15, d (*7.1*)	17.6	2	1, 2
	**NH**	7.84, d (*7.4*)	---	2	2, 3, Leu^3-1
**D-Leu^3**	**1**	---	171.0	---	---
	**2**	4.03, m	52.0	3, Leu^3-NH	1, 3, 4
	**3**	1.46, m	38.7	2, 4	1, 2, 4, 5, 6
	**4**	1.63, m	24.1	3, 5, 6	2, 3, 5, 6
	**5**	0.88, d (*6.5*)	22.9	4	3, 4, 6
	**6**	0.81, d (*6.6*)	21.0	4	3, 4, 5
	**NH**	8.62, d (*6.1*)	---	2	2, 3, Val^4-1
**L-Val^4**	**1**	---	171.7[d]	---	---
	**2**	4.10, dd (*8.6, 7.6*)	58.3	3, Val^4-NH	1, 3, 4, 5
	**3**	1.88, m	29.8	2, 4, 5	1, 2, 4, 5
	**4**	0.87, d (*6.4*)	18.9	3	2, 3, 5
	**5**	0.83, d (*6.6*)	18.7	3	2, 3, 4
	**NH**	7.92[a], d (*7.6*)	---	2	2, 3, Gly^5-1
**Gly^5**	**1**	---	168.9	---	---
	**2**	a 4.07, dd (*16.7, 6.6*)	42.3	2b, Gly^5-NH	1
		b 3.41, dd (*16.7, 3.9*)		2a, Gly^5-NH	1
	**NH**	7.91[a], dd (*6.5, 3.9*)	---	2	2, 1′
**HDMLA***	**1′**	---	169.8	---	---
	**2′**	a 2.51, dd (*15.2, 9.8*)	37.7	2′b, 3′	1′, 3′
		b 2.25, d (*15.2, 1.4*)		2′a, 3′	1′, 3′, 4′
	**3′**	4.94, ddd (*9.1, 5.1, 1.8*)	74.8	2′a, 4′	Phe^1-1, 1′, 2′, 4′, 4′-Me, 5′
	**4′**	1.68, m	33.7	3′, 4′-Me, 5′	2′, 3′, 4′-Me, 5′
	**5′**	a 1.18[b], m	39.5[e]	4′, 5′b, 6′	3′, 4′, 4′-Me, 6′-Me, 7′
		b 0.75, m		5′a	3′, 4′, 4′-Me, 6′, 6′-Me, 7′
	**6′**	1.41, m	29.3	5′, 6′-Me, 7′b	7′, 8′
	**7′**	a 1.19[b], m	35.7	7′b	
		b 0.95, m		6′, 7′a	6′-Me, 8′, 9′
	**8′**	1.20[b], m	26.1		
	**9′**	1.22[c], m	29.1		
	**10′**	1.22[c], m	31.3		
	**11′**	1.24, m	22.1	12′	9′, 10′, 12′
	**12′**	0.84, t (*6.9*)	13.9	11′	10′, 11′
	**4′-Me**	0.70, d (*6.7*)	14.8	4′	3′, 4′, 5′
	**6′-Me**	0.80, d (*6.6*)	20.2	6′	5′, 6′, 7′

* HDMLA: *3S*, *4S*, *6S*-3-Hydroxy-4, 6-dimethyllauric acid. (a - c) overlapping resonances within the same letter. (d) signals are interchangeable within the same letter. (e) signal obscured by the solvent.

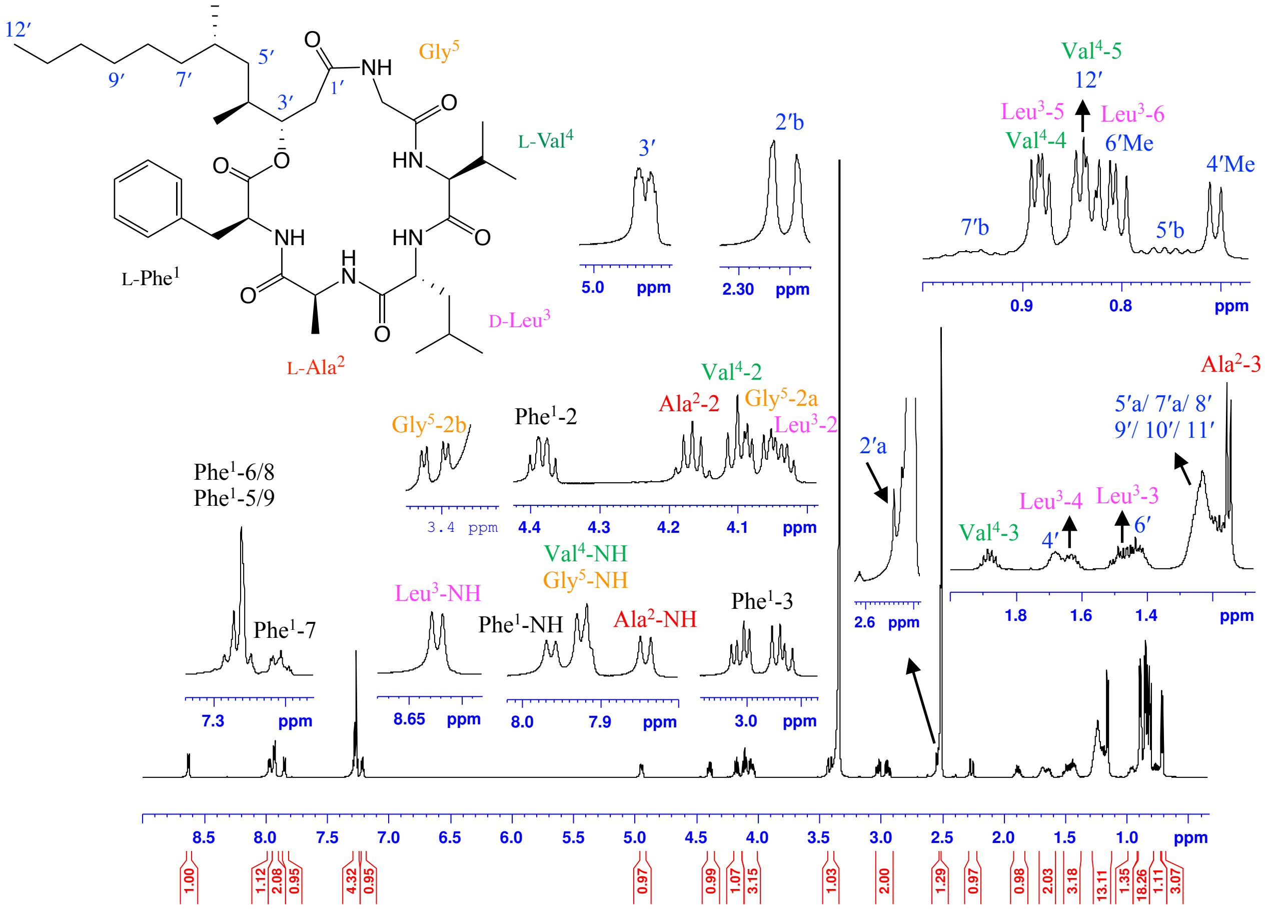

Figure S14. ^{1}H NMR (600 MHz, DMSO-d_6) spectrum for scopularide C (**3**)

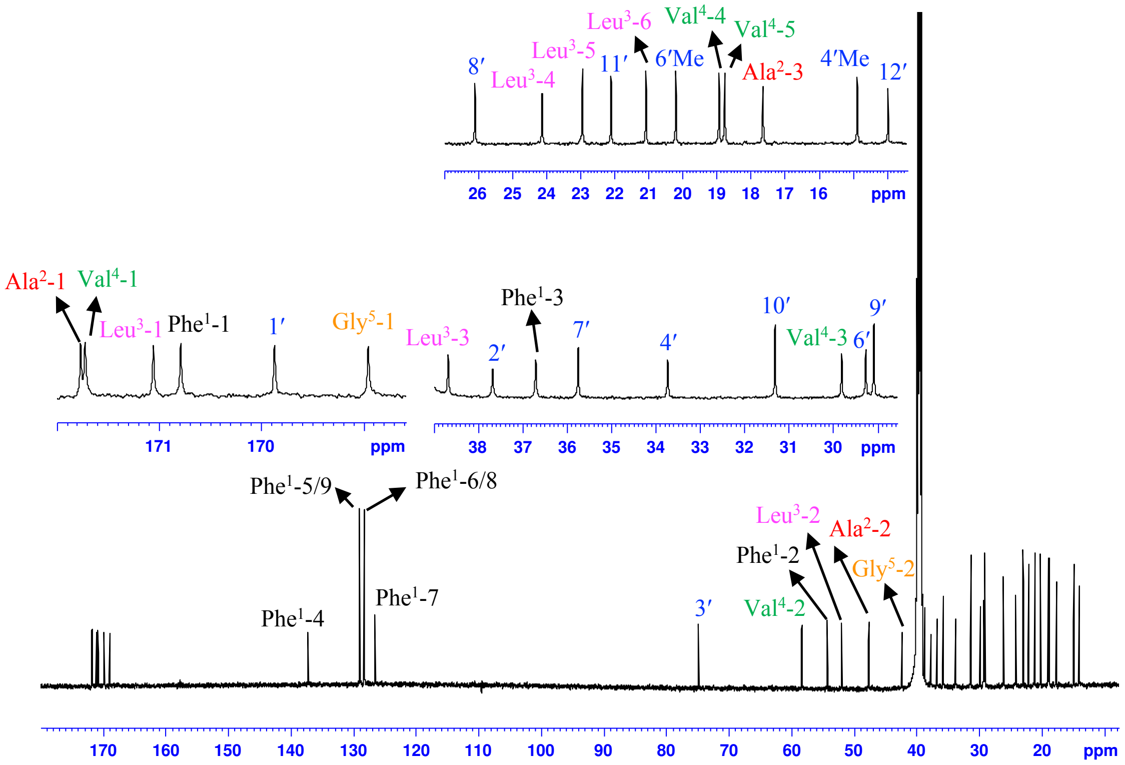

Figure S15. ^{13}C NMR (150 MHz, DMSO-d_6) spectrum for scopularide C (**3**)

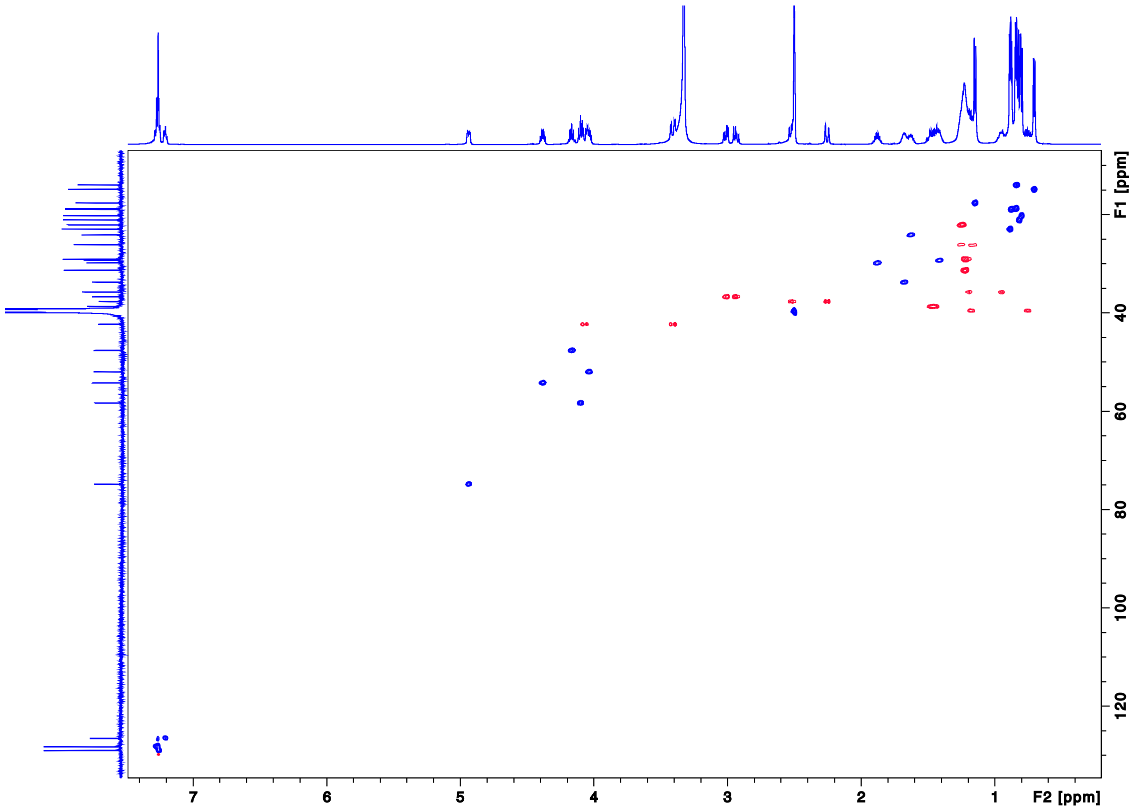

Figure S16. HSQC NMR (600 MHz, DMSO-d_6) spectrum for scopularide C (**3**)

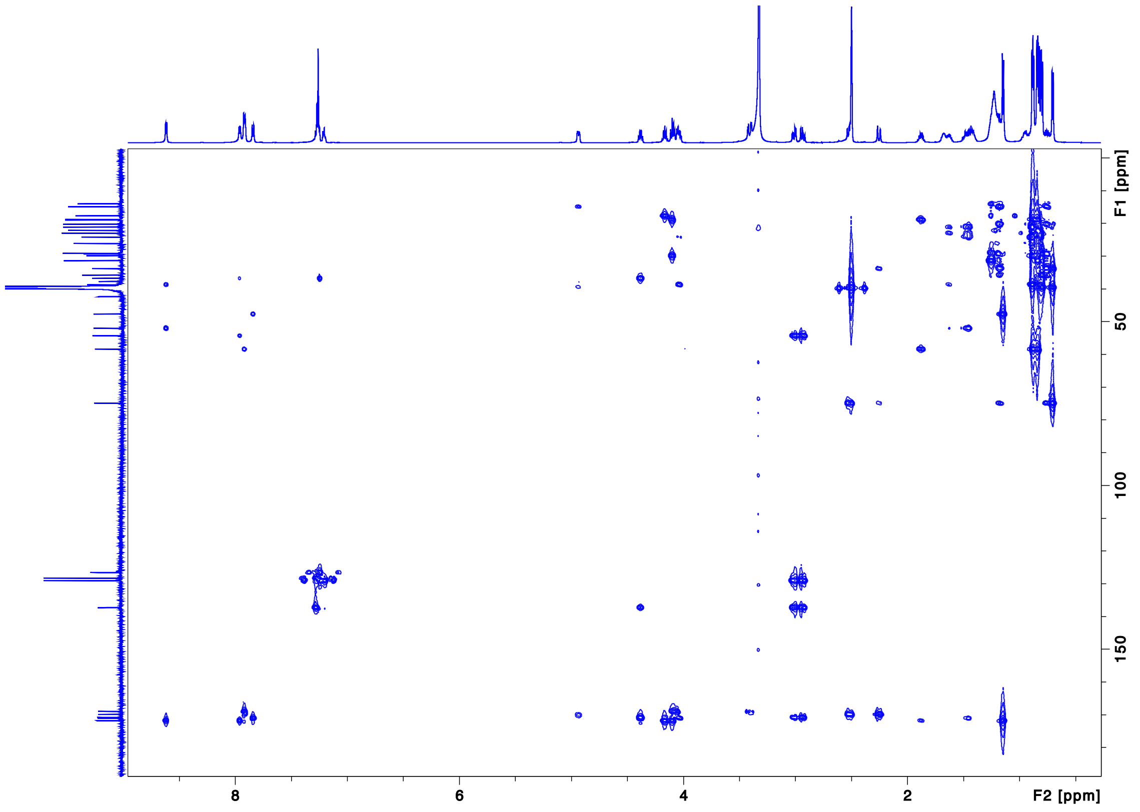

Figure S17. HMBC NMR (600 MHz, DMSO-d_6) spectrum for scopularide C (**3**)

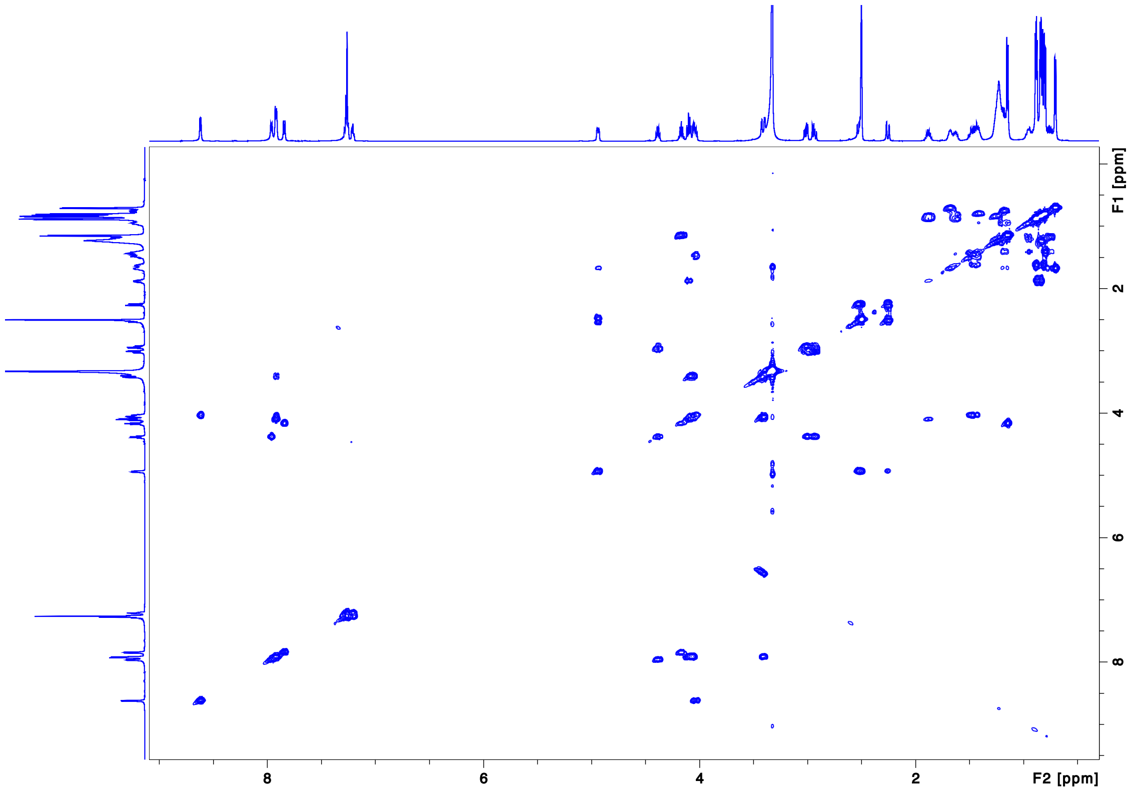

Figure S18. COSY NMR (600 MHz, DMSO-d_6) spectrum for scopularide C (**3**)

2.4 Scopularide D (4)

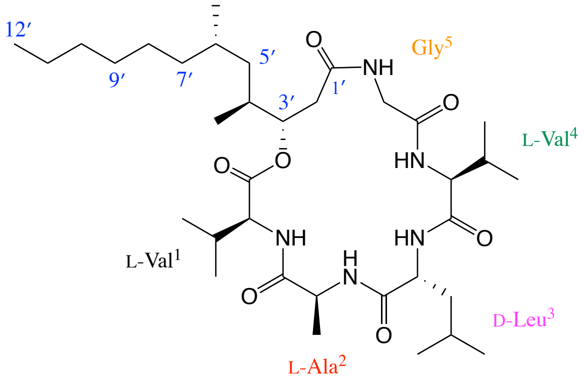

Table S6. 1D and 2D **NMR (600 MHz, DMSO-d_6) data for scopularide D (4)**

**Position**		**δ_H, mult (*J* in Hz)**	**δ_C**	**COSY**	**^{1}H-^{13}C HMBC**
**L-Val^1**	**1**	---	170.7	---	---
	**2**	4.15, dd (*7.8, 5.3*)	57.3	3, Val^1-NH	1, 3, 4, 5
	**3**	2.08, m	29.7	2, 4, 5	1, 2, 4, 5
	**4**	0.87, d (*6.8*)	19.0	3	5
	**5**	0.85[e], d (*7.0*)	17.4	3	4
	**NH**	7.41, d (*7.1*)	---	2	2, Ala^2-1
**L-Ala^2**	**1**	---	171.8	---	---
	**2**	4.19, dq (*8.0, 7.1*)	47.9	3, Ala^2-NH	1, 3
	**3**	1.21, d (*7.1*)	17.5	2	1, 2
	**NH**	8.03, d (*8.0*)	---	2	2, 3, Leu^3-1
**D-Leu^3**	**1**	---	171.3	---	---
	**2**	4.04, dd (*10.1, 6.5*)	51.9	3, Leu^3-NH	1, 3, 4
	**3**	1.48, m	38.7	2, 4	1, 2, 4, 5, 6
	**4**	1.63, m	24.1	3, 5, 6	2, 3, 5, 6
	**5**	0.89[c], d (*6.7*)	22.9	4	3, 4, 6
	**6**	0.81, d (*6.6*)	21.0	4	3, 5
	**NH**	8.63, d (*6.5*)	---	2	2, 3, Val^4-1
**L-Val^4**	**1**	---	171.6	---	---
	**2**	4.06[a], m	58.6	3, Val^4-NH	1, 3, 4, 5
	**3**	1.86, m	29.5	2, 4, 5	1, 2, 5
	**4**	0.88[c], d (*6.8*)	19.0	3	5
	**5**	0.83[d], d (*6.8*)	18.7	3	4
	**NH**	8.06, d (*7.8*)	---	2	2, 3, Gly^5-1
**Gly^5**	**1**	---	169.0	---	---
	**2a**	a 4.06[a], m	42.0	2b, Gly^5-NH	1
	**b**	b 3.44, dd (*17.1, 3.8*)		2a, Gly^5-NH	1
	**NH**	7.90, dd (*5.8, 3.8*)	---	2	2, 1′
**HDMLA***	**1′**	---	170.0	---	---
	**2′a**	a 2.52, dd (*14.5, 9.6*)	38.0	2′b,3′	1′, 3′
	**b**	b 2.23, dd (*14.5, 1.4*)		2′a, 3′	1′, 3′, 4′
	**3′**	4.92, ddd (*9.3, 4.8, 1.4*)	75.2	2′, 4′	Val^1-1, 1′, 2′, 5′, 4′-Me
	**4′**	1.79, m	33.9	3′, 4′-Me, 5′	4′-Me
	**5′a**	a 1.29, m	39.6[f]	4′, 5′b, 6′	3′, 4′, 4′-Me, 6′, 6′-Me, 7′
	**b**	b 0.87, m		5′a	
	**6′**	1.44, m	29.3	5′, 6′-Me, 7′b	
	**7′a**	a 1.22, m	36.0	7′b	
	**b**	b 0.98, m		6′, 7′a, 8′b	
	**8′a**	a 1.26, m	26.1	8′b	10′
	**b**	b 1.16, m		7′b, 8′a	9′
	**9′a**	a 1.22[b], m	29.1		11′
	**b**	b 1.19, m			
	**10′**	1.23[b], m	31.3		
	**11′**	1.25, m	22.1		
	**12′**	0.85[e], t (*7.9*)	13.9	11′	10′, 11′
	**4′-Me**	0.84[d], d (*8.0*)	15.1	4′	3′, 4′, 5′
	**6′-Me**	0.83[d], d (*7.4*)	20.0	6′	6′

* HDMLA: *3S, 4S, 6S*-3-Hydroxy-4, 6-dimethyllauric acid

(a – e) overlapping resonances within the same letter. (f) signal obscured by the solvent

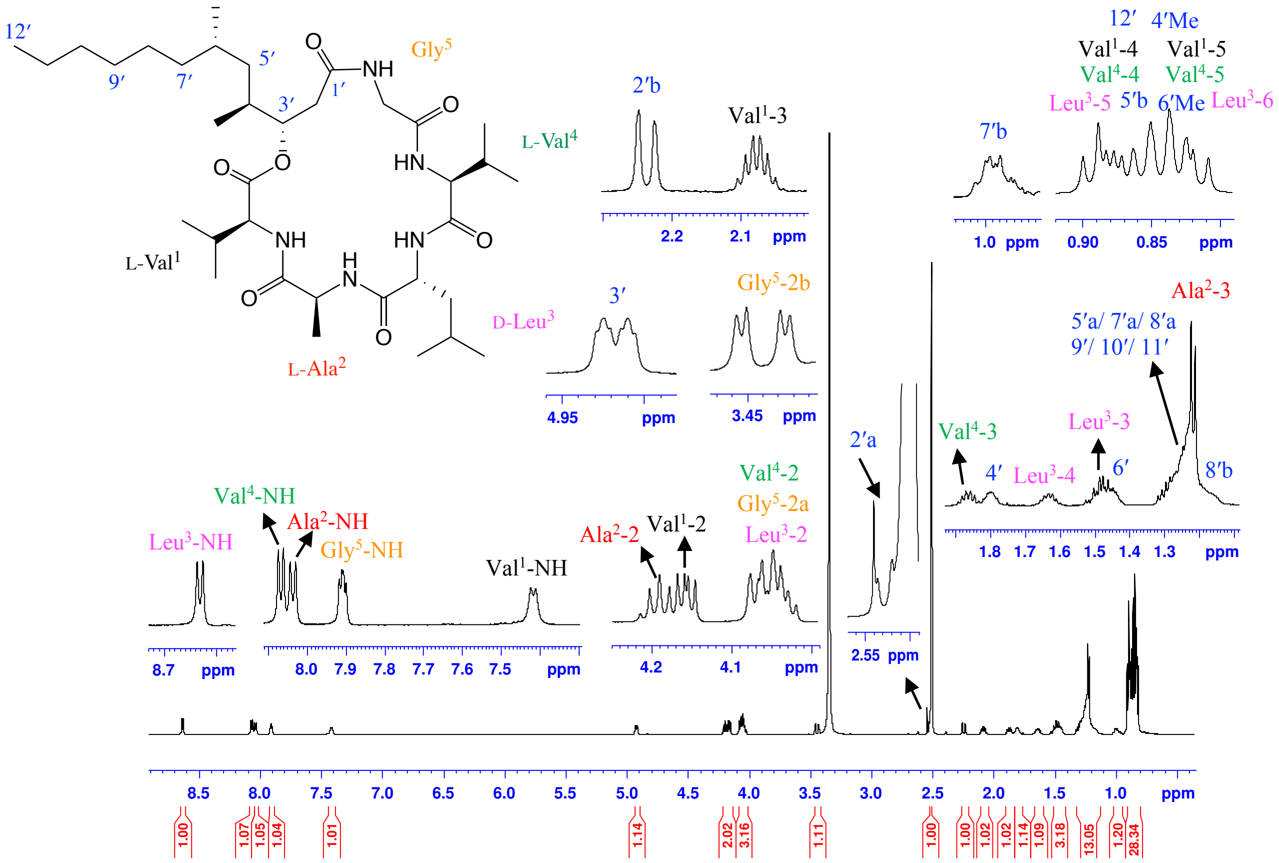

Figure S19. ^{1}H NMR (600 MHz, DMSO-d_6) spectrum for scopularide D (**4**)

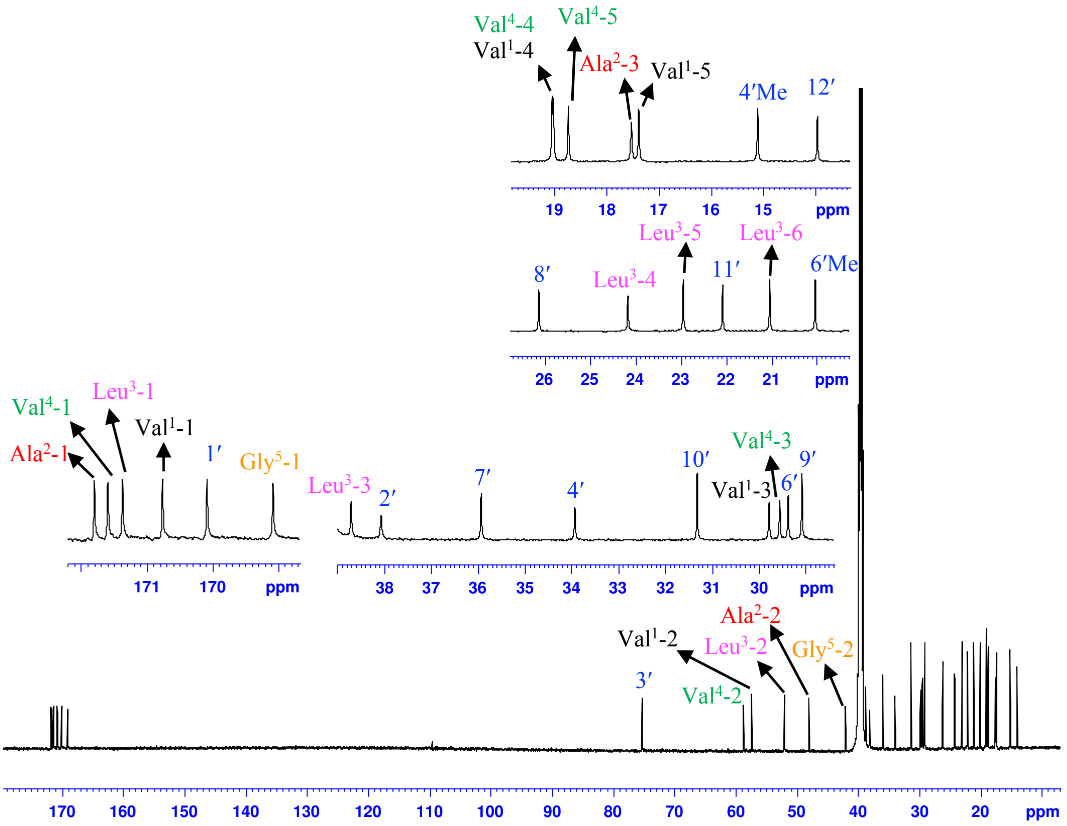

Figure S20. ^{13}C NMR (150 MHz, DMSO-d_6) spectrum for scopularide D (**4**)

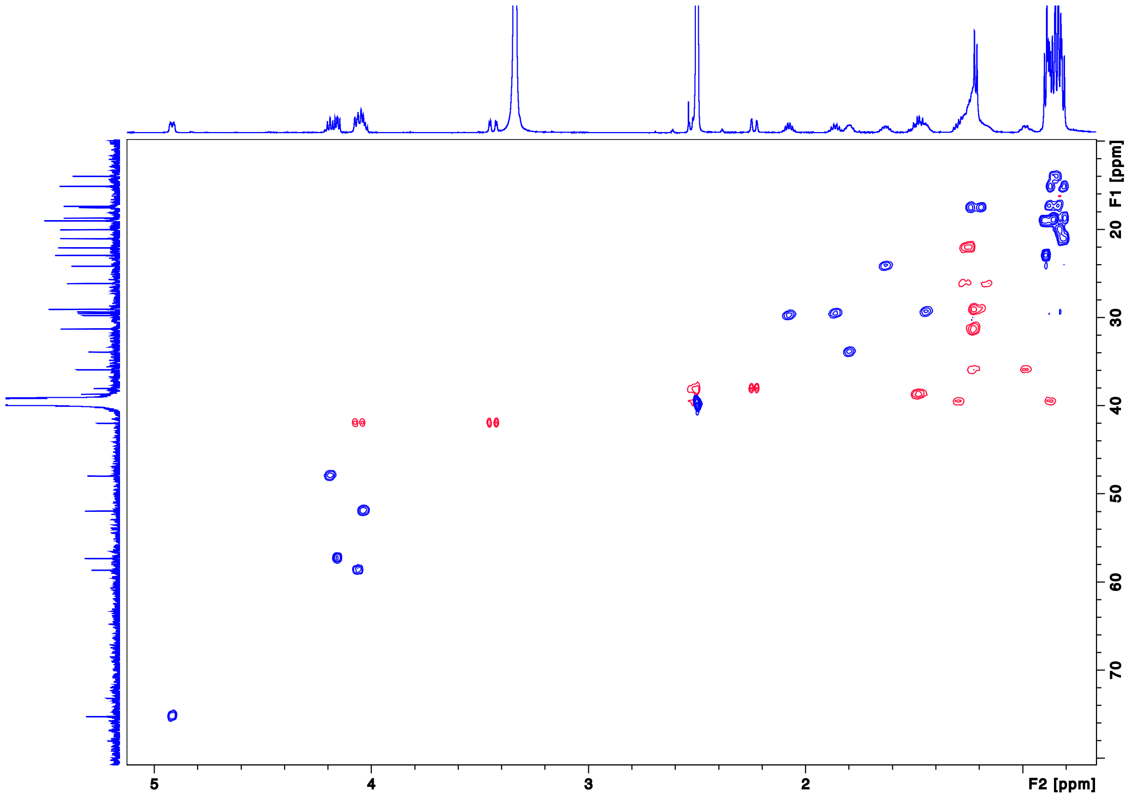

Figure S21. HSQC NMR (600 MHz, DMSO-d_6) spectrum for scopularide D (**4**)

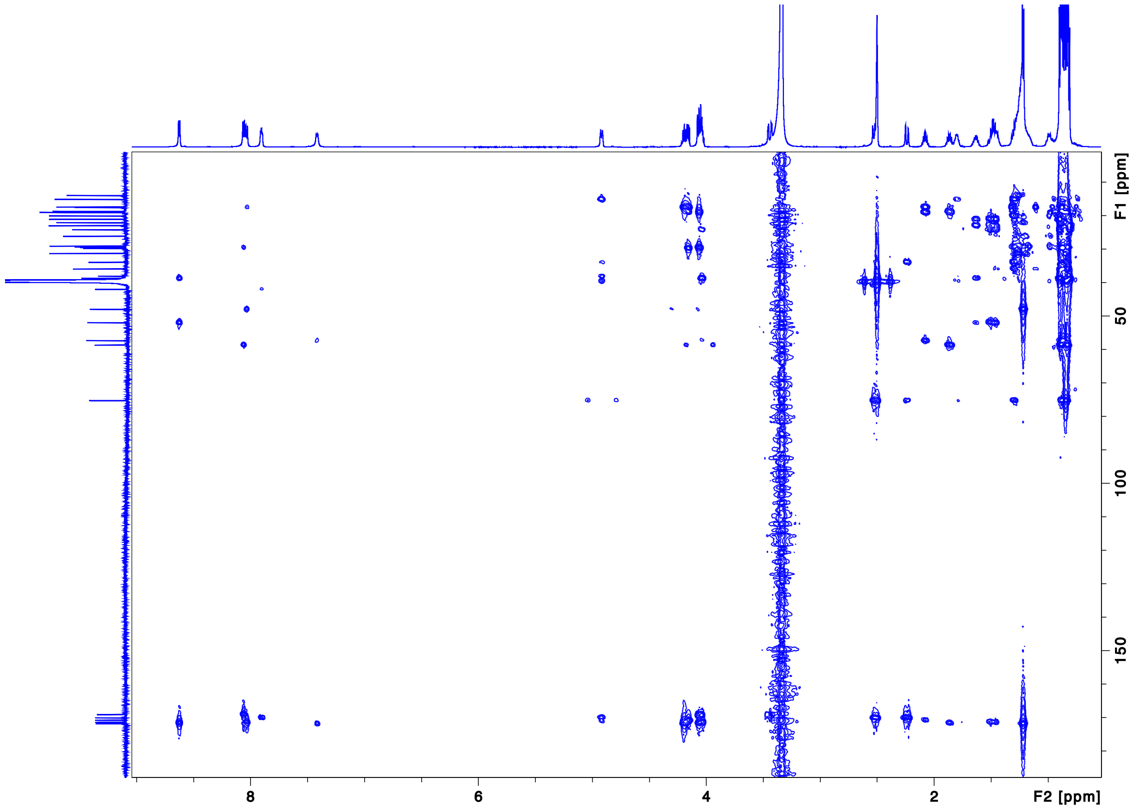

Figure S22. HMBC NMR (600 MHz, DMSO-d_6) spectrum for scopularide D (**4**)

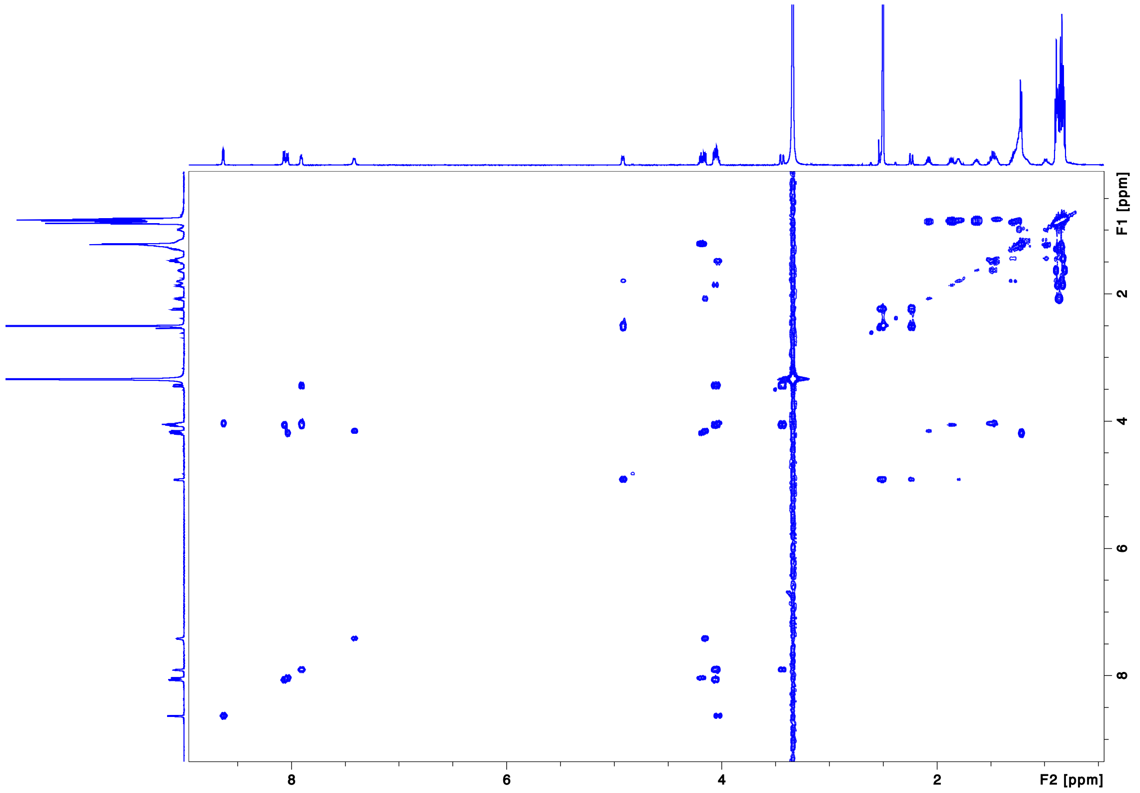

Figure S23. COSY NMR (600 MHz, DMSO-d_6) spectrum for scopularide D (**4**)

2.5 Scopularide E (5)

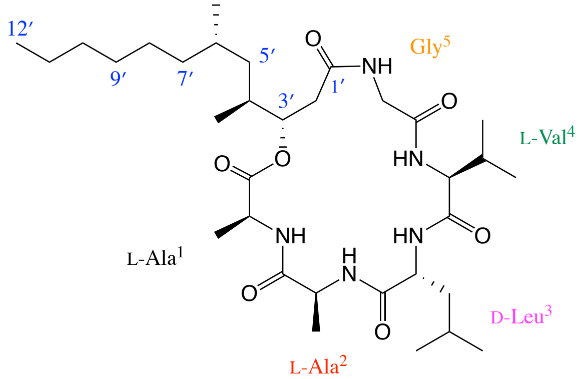

Table S7. 1D and 2D NMR (600 MHz, DMSO-d_6) data for scopularide E (5)

Position		δ_H, mult (*J* in Hz)	δ_C	COSY	^{1}H-^{13}C HMBC
**L-Ala^{1}**	**1**	---	171.7[c]	---	---
	**2**	4.13, dq (*7.4, 6.0*)	48.1	3, Ala^{1}-NH	1, 3
	**3**	1.28, d (*7.4*)	16.7	2	1, 2
	**NH**	7.80, d (*6.0*)	---	2	2, 3, Ala^{2}-1
**L-Ala^{2}**	**1**	---	171.5	---	---
	**2**	4.18, dq (*7.1, 6.5*)	47.5	3, Ala^{2}-NH	1, 3
	**3**	1.19, d (*7.1*)	17.4	2	1, 2
	**NH**	7.89, d (*6.5*)	---	2	2, 3, Leu^{3}-1
**D-Leu^{3}**	**1**	---	171.1	---	---
	**2**	4.03, dd (*11.4, 6.3*)	51.8	3, Leu^{3}-NH	1, 3, 4
	**3**	a 1.49, m	38.6	2, 4	2, 4, 5, 6
		b 1.47[a], m		2, 4	2, 4, 5, 6
	**4**	1.63, m	24.1	3, 5, 6	2, 3, 5, 6
	**5**	0.89, d (*6.7*)	23.0	4	3, 4, 6
	**6**	0.81, d (*6.5*)	21.1	4	3, 4, 5
	**NH**	8.64, d (*6.3*)	---	2	2, 3, Val^{4}-1
**L-Val^{4}**	**1**	---	171.6[c]	---	---
	**2**	4.11, dd (*8.2, 6.6*)	58.1	3, Val^{4}-NH	1, 3, 4, 5, Gly^{5}-1
	**3**	1.85, m	29.9	2, 4, 5	1, 2, 4, 5
	**4**	0.87, d (*6.9*)	18.8	3	2, 3, 5
	**5**	0.83, d (*6.7*)	18.7	3	2, 3, 4
	**NH**	7.87, d (*6.6*)	---	2	2, 3, Gly^{5}-1
**Gly^{5}**	**1**	---	168.8	---	---
	**2**	a 4.06, dd (*16.5, 6.6*)	42.3	2b, Gly^{5}-NH	1
		b 3.43, dd (*16.5, 4.0*)		2a, Gly^{5}-NH	1
	**NH**	7.82, dd (*6.1, 4.0*)	---	2	2, 1′
**HDMLA***	**1′**	---	169.8	---	---
	**2′**	a 2.53, dd (*14.7, 10.1*)	37.7	2′b,3′	1′, 3′
		b 2.24, dd (*14.7, 1.8*)		2′a, 3′	1′, 3′, 4′
	**3′**	4.97, ddd (*10.1, 4.8, 1.8*)	74.2	2′, 4′	Ala^{1}-1, 1′, 2′, 5′, 4′-Me
	**4′**	1.75, m	33.8	3′, 5a′, 4′-Me	5′
	**5′**	a 1.26, m	39.6[d]	4′, 6′	3′, 4′-Me, 6′-Me
		b 0.84, m		4′, 6′	
	**6′**	1.46[a], m	29.2	5′a, 7′b, 6′-Me	7′, 8′
	**7′**	a 1.23[b], m	35.8		
		b 0.99, m		6′, 8′a	
	**8′**	a 1.24, m	26.1		6′
		b 1.16, m		7′b	6′, 9′
	**9′**	1.23[b], m	29.0		
	**10′**	1.23[b], m	31.3		
	**11′**	1.26, m	22.1		
	**12′**	0.85, t (*7.1*)	13.9	11′	10′, 11′
	**4′-Me**	0.82, d (*6.4*)	15.0	4′	3′, 4′
	**6′-Me**	0.83, d (*6.7*)	20.2	6′	7′

* HDMLA: *3S, 4S, 6S*-3-Hydroxy-4, 6-dimethyllauric acid. (a – b) overlapping resonances within the same letter. (c) signals are interchangeable within the same letter. (d) signal obscured by the solvent

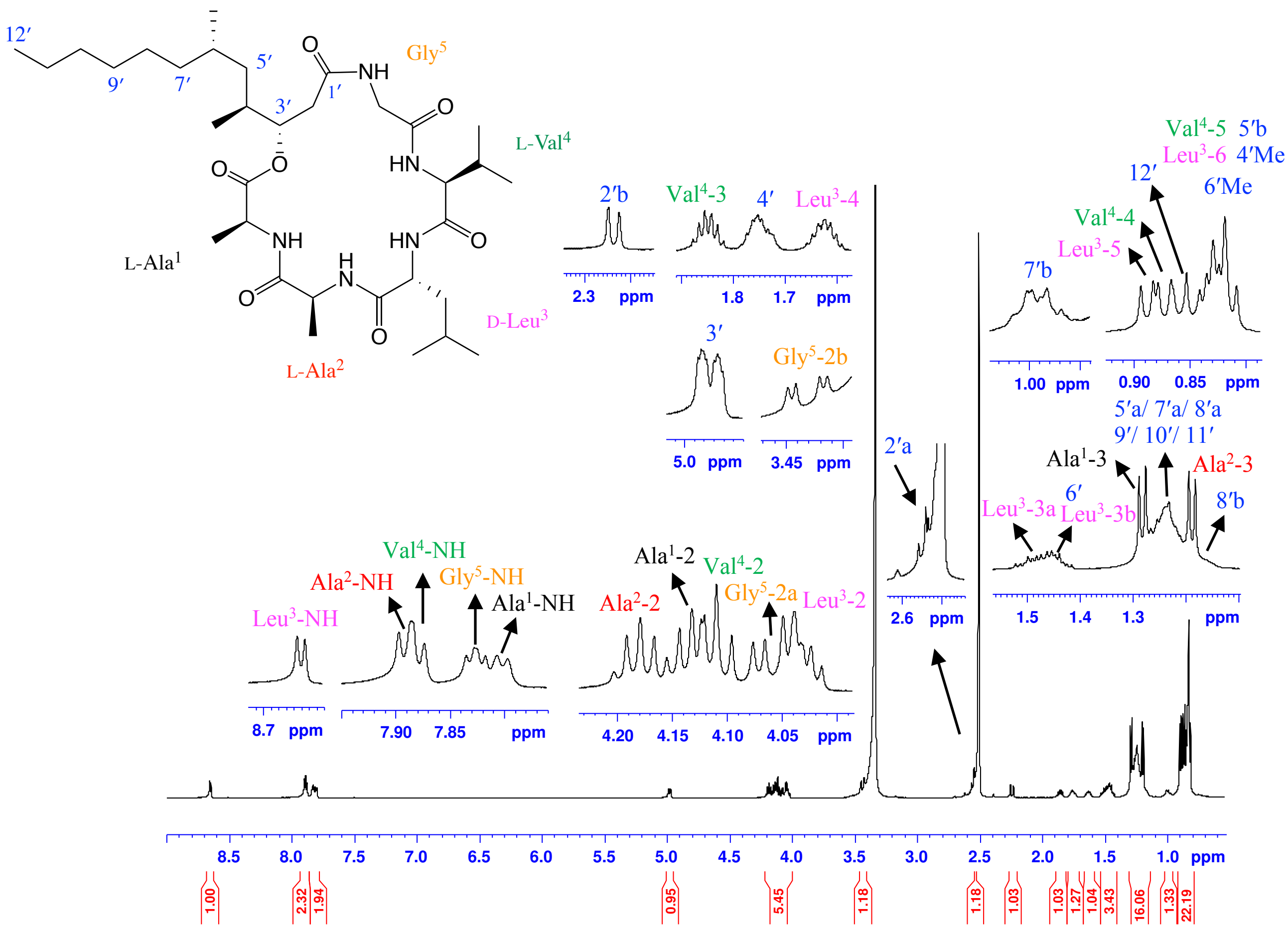

Figure S24. ^{1}H NMR (600 MHz, DMSO-d_6) spectrum for scopularide E (**5**)

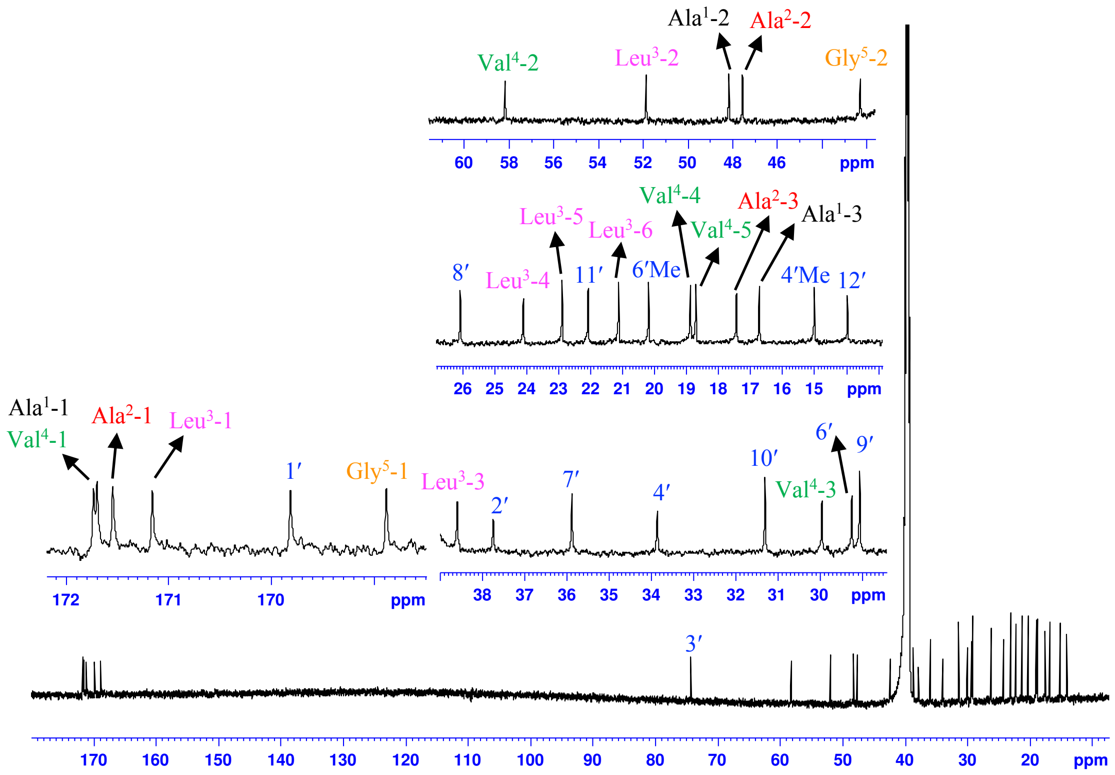

Figure S25. ^{13}C NMR (150 MHz, DMSO-d_6) spectrum for scopularide E (**5**)

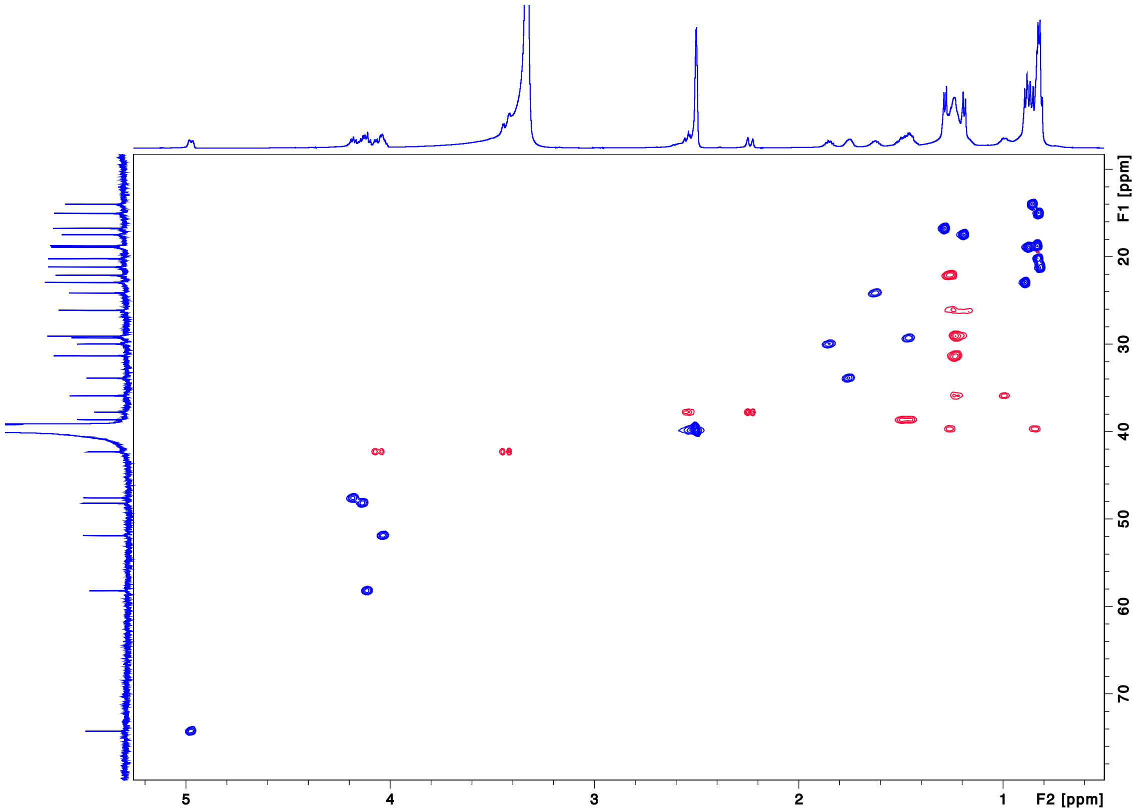

Figure S26. HSQC NMR (600 MHz, DMSO-d_6) spectrum for scopularide E (**5**)

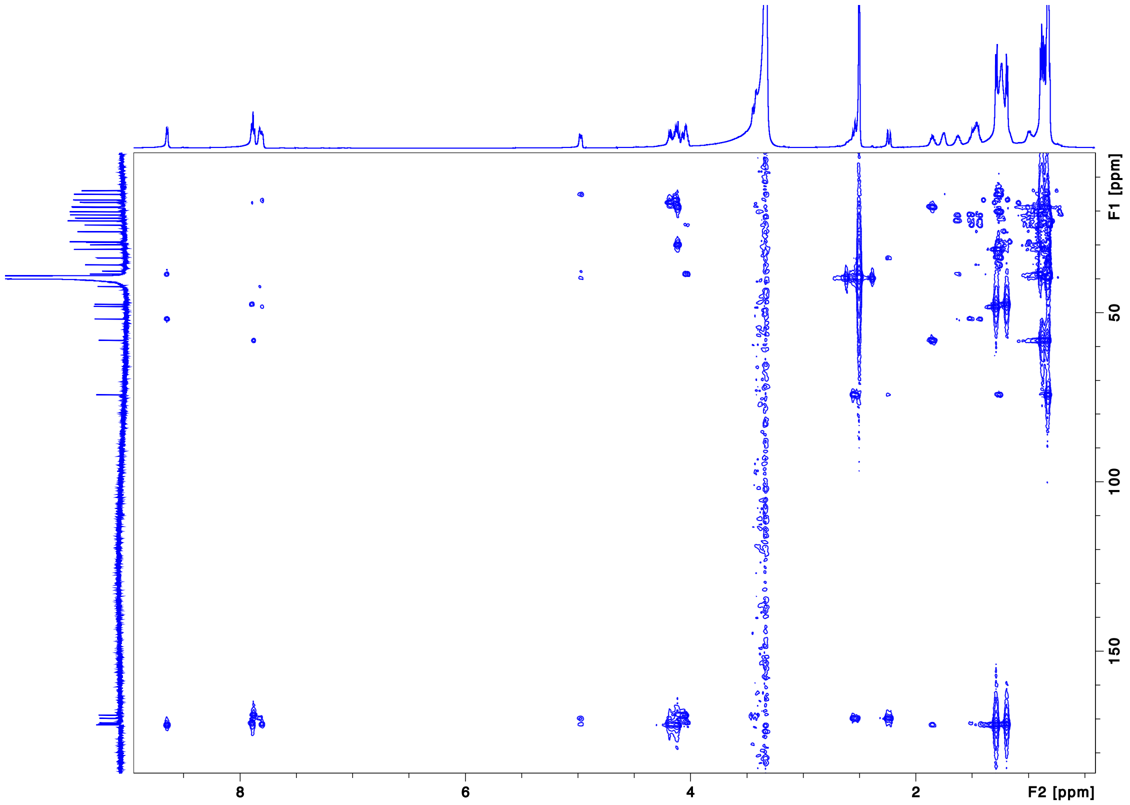

Figure S27. HMBC NMR (600 MHz, DMSO-d_6) spectrum for scopularide E (**5**)

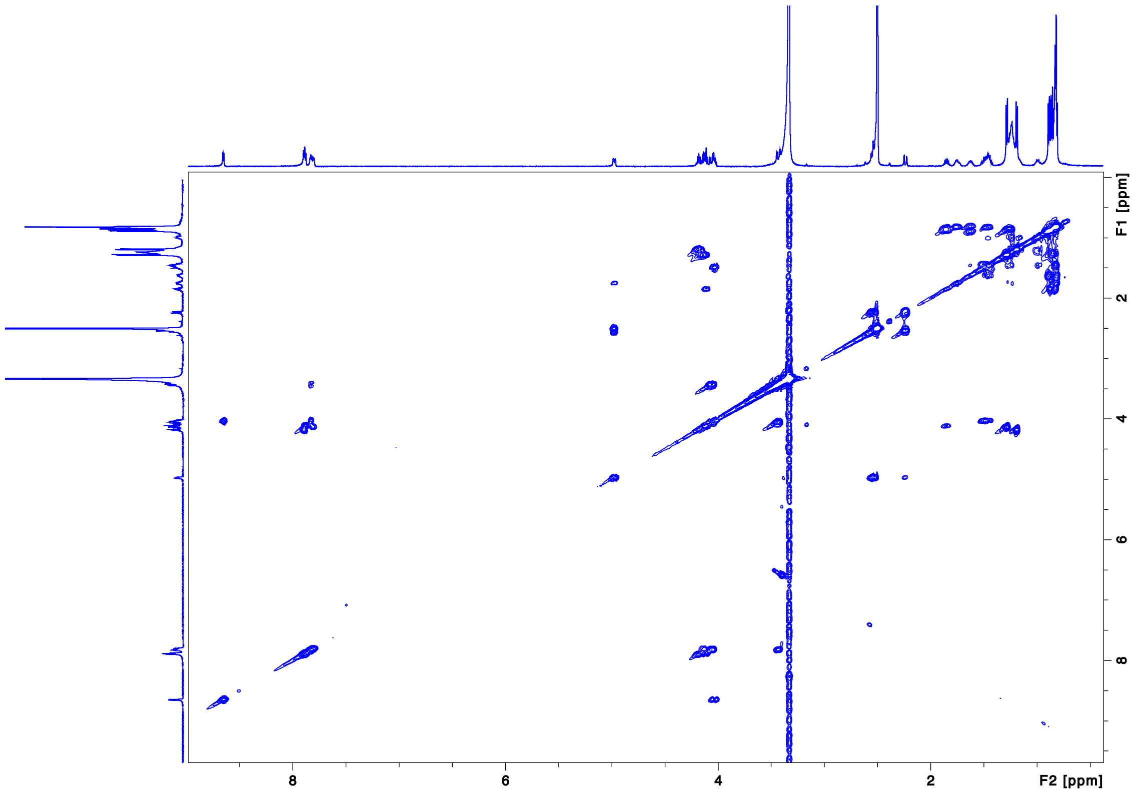

Figure S28. COSY NMR (600 MHz, DMSO-d_6) spectrum for scopularide E (**5**)

2.6 Scopularide F (6)

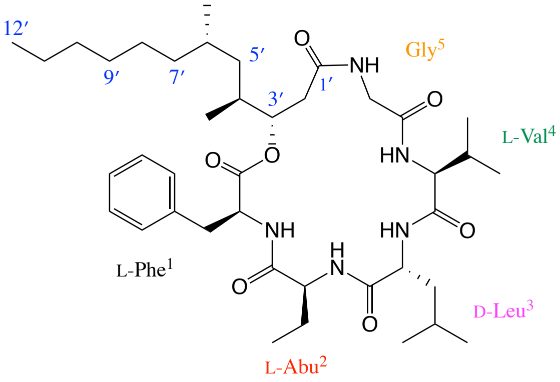

Table S8. 1D and 2D NMR (600 MHz, DMSO-d_6) data for scopularide F (6)

Position		δ_H, mult (*J* in Hz)	δ_C	COSY	^{1}H-^{13}C HMBC
L-Phe^1	1	---	170.9	---	---
	2	4.38, m	54.3	3, Phe^1-NH	1, 3, 4
	3	a 2.97, dd (*13.9, 6.1*)	36.7	2	1, 2, 5/9, 4
		b 2.95, dd (*13.9, 8.4*)			
	4	---	137.1	---	---
	5/9	7.26, m	128.9		3, 5/9, 7
	6/8	7.28, m	128.3	7	4, 6/8
	7	7.21, m	126.6	6/8	5/9
	NH	8.14, d (*6.5*)	---	2	2, 3, Abu^2-1
L-Abu^2*	1	---	171.0	---	---
	2	4.08^a, m	52.9	3a, Abu^2-NH	1, 3, 4
	3	a 1.74, m	24.4	3b, 4	1, 2, 4
		b 1.48^b, m		3a, 4	1, 2, 4
	4	0.73, t (*7.2*)	9.5	3	2, 3
	NH	7.67, d (*8.0*)	---	2	2, Leu^3-1
D-Leu^3	1	---	171.1	---	---
	2	4.09^a, m	51.8	3, Leu^3-NH	1, 3
	3	1.47^b, m	38.6	2, 4	1, 2, 5, 6
	4	1.61, m	24.1	3, 5, 6	2, 3, 5, 6
	5	0.88, d (*6.5*)	22.8	4	3, 4, 6
	6	0.81, d (*6.8*)	21.2	4	3, 4, 5
	NH	8.62, d (*6.3*)	---	2	2, 3, Val^4-1
L-Val^4	1	---	171.6	---	---
	2	4.15, dd (*8.9, 8.7*)	58.1	3, Val^4-NH	1, 3, 4, 5, Gly^5-1
	3	1.88, m	29.9	2, 4, 5	2, 4, 5
	4	0.86, d (*6.7*)	18.8	3	2, 3, 5
	5	0.83^f, d (*6.6*)	18.7	3	2, 3, 4
	NH	7.86, d (*8.9*)	---	2	2, Gly^5-1
Gly^5	1	---	168.6	---	---
	2	a 4.01, dd (*16.7, 6.3*)	42.4	2b, Gly^5-NH	1
		b 3.42, dd (*16.7, 4.7*)		2a, Gly^5-NH	1
	NH	7.85, dd (*5.6, 4.7*)	---	2	1′
HDMLA**	1′	---	169.8	---	---
	2′	a 2.53, dd (*14.8, 9.4*)	37.9	2′b, 3′	1′, 3′
		b 2.24, dd (*14.8, 1.2*)		2′a, 3′	1′, 4′
	3′	4.94, ddd (*9.1, 3.2, 1.8*)	74.7	2′, 4′	Phe^1-1, 1′, 4′-Me, 5′
	4′	1.66, m	33.9	3′, 4′-Me, 5′	
	5′	a 1.17^e, m	39.6^g	4′, 5′b, 6′	3′, 4′, 4′-Me, 6′, 6′-Me, 7′
		b 0.75, m		5′a	3′, 4′, 4′-Me, 6′, 6′-Me, 7′
	6′	1.41, m	29.2	5′a, 6′-Me, 7′b	
	7′	a 1.19, m	35.8	6′, 7′b	
		b 0.95, m		6′, 7′a	
	8′	a 1.25^c, m	26.1		10′
		b 1.17^e, m			
	9′	1.22^d, m	29.1		
	10′	1.22^d, m	31.3		
	11′	1.24^c, m	22.1		
	12′	0.83^f, t (*6.9*)	13.9	11′	10′, 11′
	4′-Me	0.69, d (*6.7*)	14.8	4′	3′, 4′, 5′
	6′-Me	0.80, d (*6.8*)	20.1	6′	5′, 6′, 7′

* L-Abu: L-α amino butyric acid, ** HDMLA: *3S, 4S, 6S*-3-Hydroxy-4, 6-dimethyllauric acid
(a – f) overlapping resonances within the same letter. (g) signal obscured by the solvent

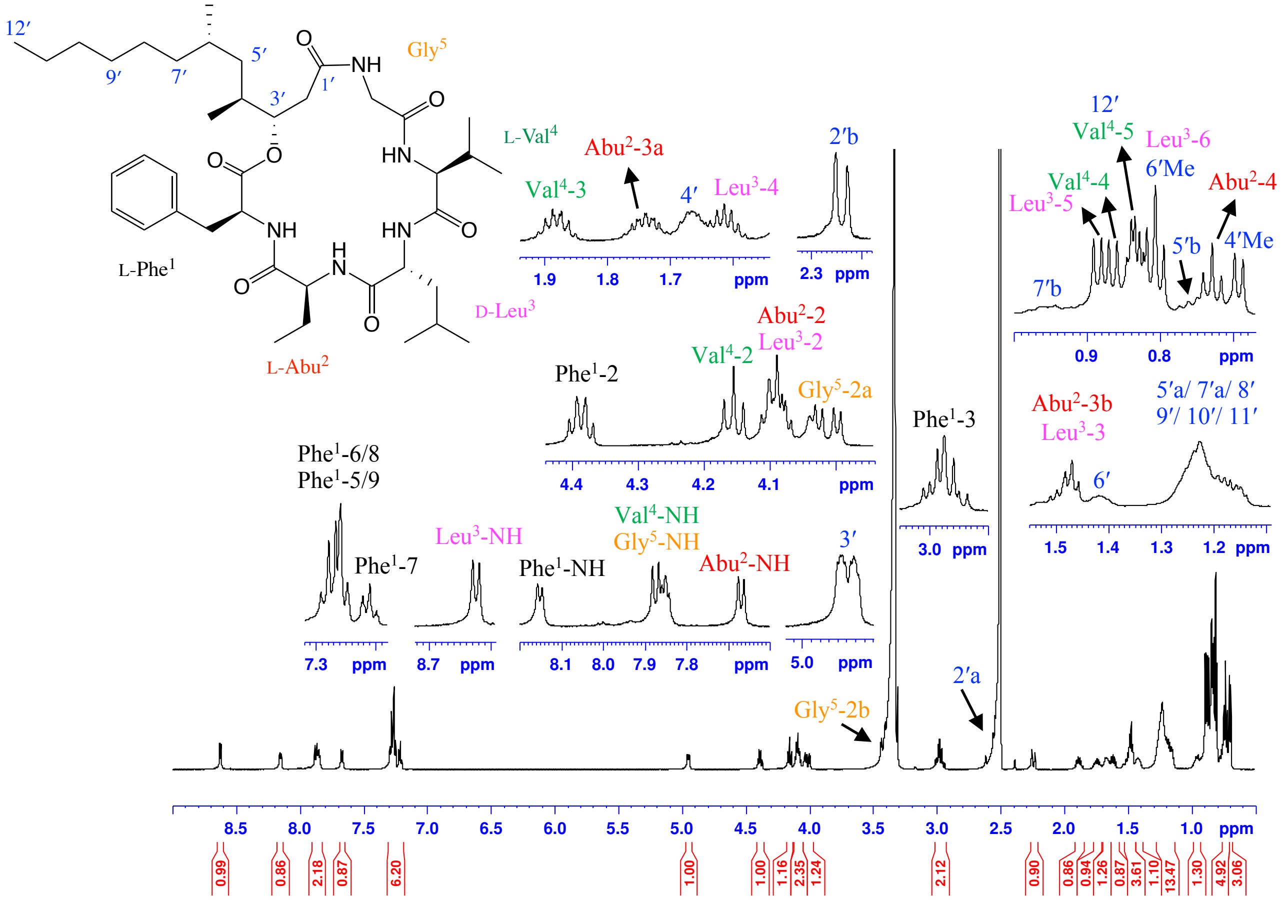

Figure S29. ^{1}H NMR (600 MHz, DMSO-d_6) spectrum for scopularide F (**6**)

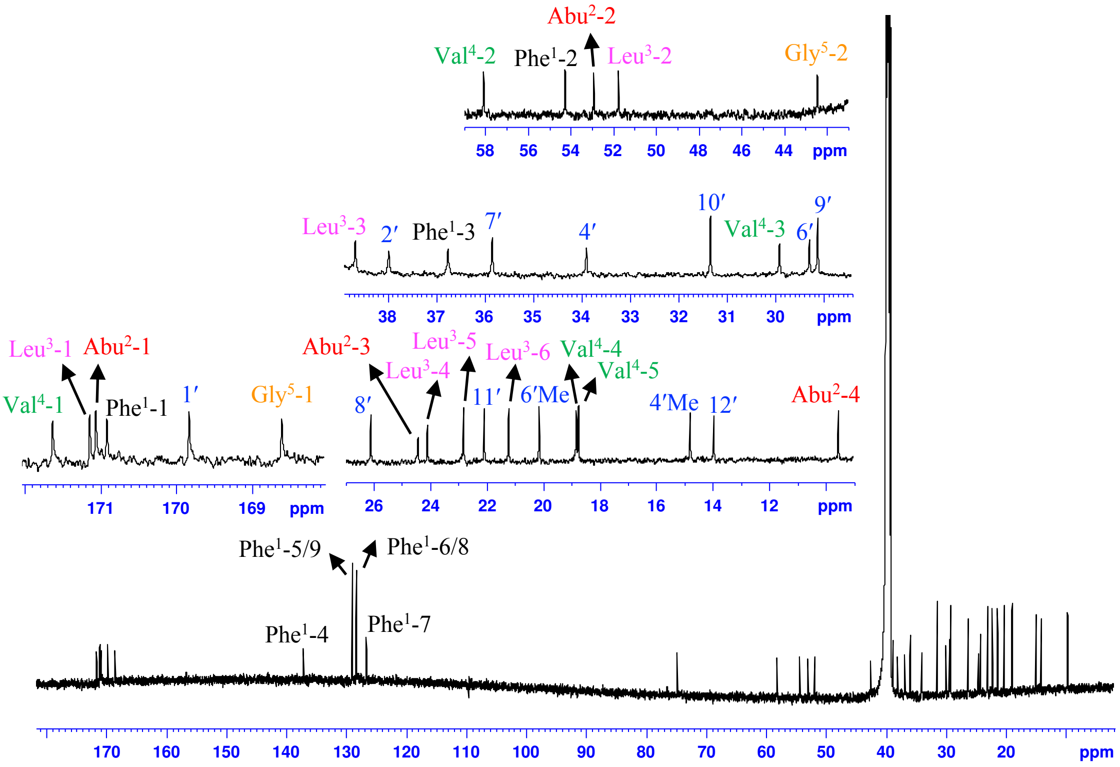

Figure S30. ^{13}C NMR (150 MHz, DMSO-d_6) spectrum for scopularide F (**6**)

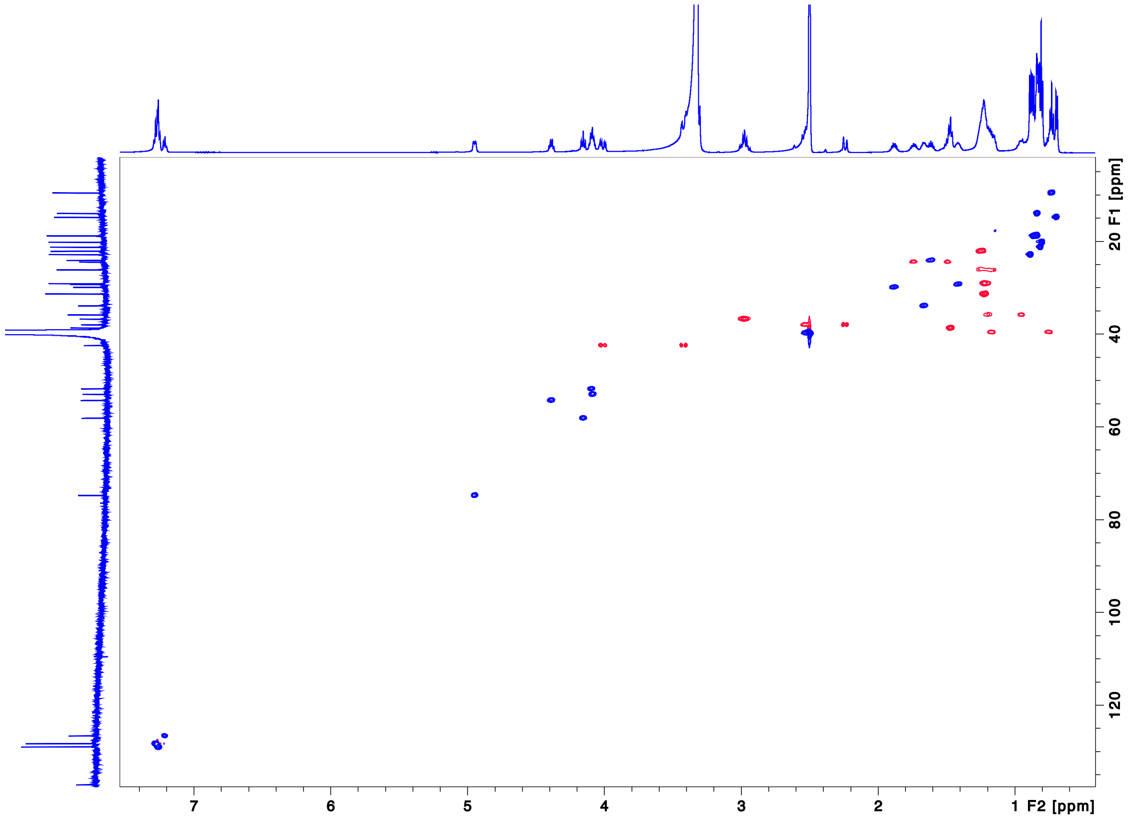

Figure S31. HSQC NMR (600 MHz, DMSO-d_6) spectrum for scopularide F (**6**)

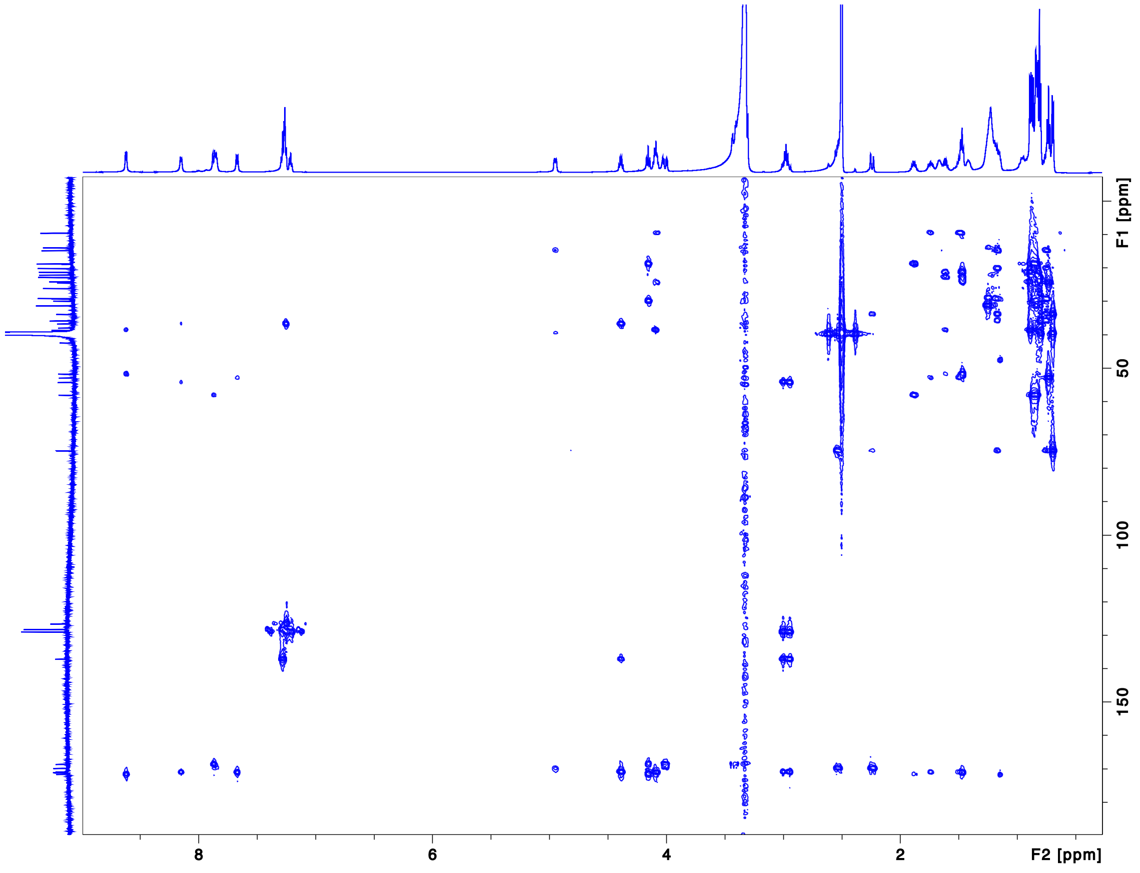

Figure S32. HMBC NMR (600 MHz, DMSO-d_6) spectrum for scopularide F (**6**)

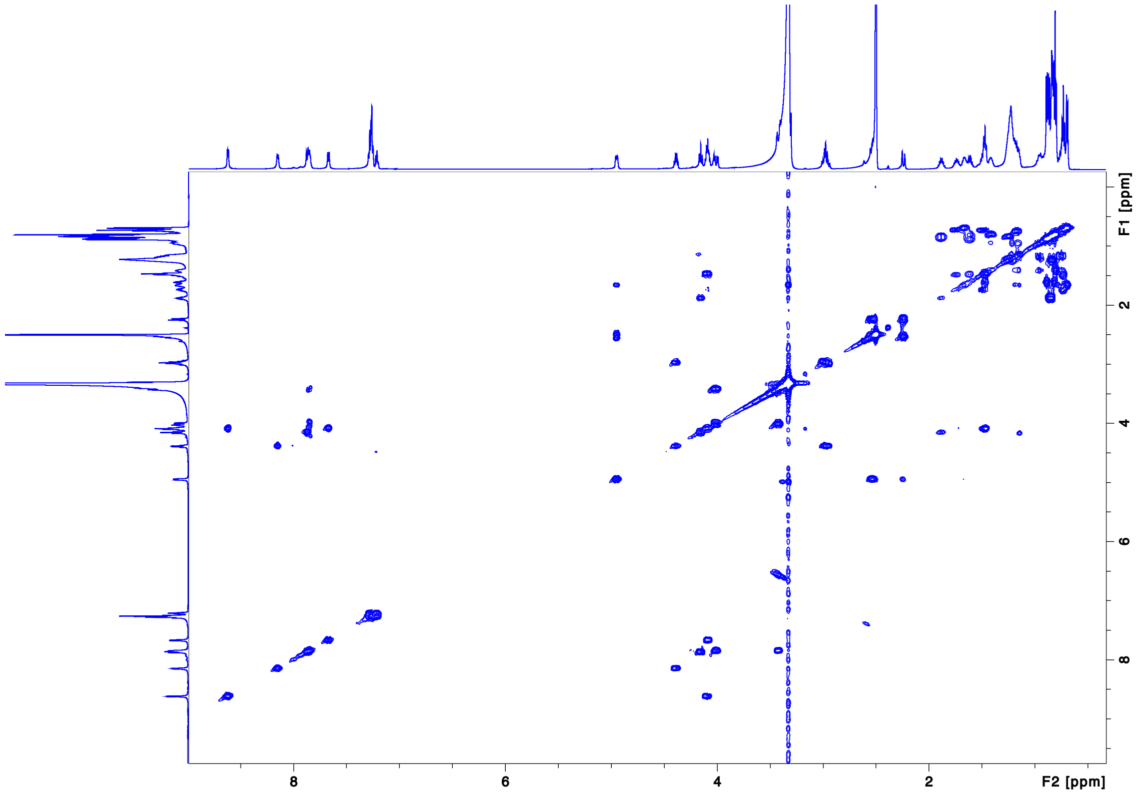

Figure S33. COSY NMR (600 MHz, DMSO-d_6) spectrum for scopularide F (**6**)

2.7 Scopularide H (8)

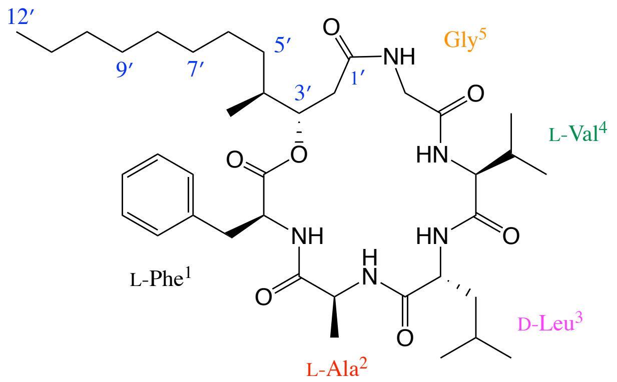

Table S9. 1D and 2D NMR (600 MHz, DMSO-d_6) data for scopularide H (8)

Position		δ_H, mult (*J* in Hz)	δ_C	COSY	^{1}H-^{13}C HMBC
**L-Phe[1]**	**1**	---	170.8	---	---
	**2**	4.34, q (*7.4*)	54.5	3, Phe[1]-NH	1, 3, 4
	**3**	2.98, m	36.7	2	1, 2, 4, 5/9
	**4**	---	137.2	---	---
	**5/9**	7.26, m	129.1		3, 7
	**6/8**	7.26, m	128.2	7	4, 6/8
	**7**	7.20, m	126.5	6/8	5/9
	**NH**	8.03, d (*6.7*)	---	2	2, 3, Ala[2]-1
**L-Ala[2]**	**1**	---	171.8	---	---
	**2**	4.19, dq (*8.1, 7.1*)	47.6	3, Ala[2]-NH	1, 3
	**3**	1.15, d (*7.1*)	17.6	2	1, 2
	**NH**	7.83, d (*8.1*)	---	2	2, 3, Leu[3]-1
**D-Leu[3]**	**1**	---	171.0	---	---
	**2**	4.03, m	52.0	3, Leu[3]-NH	1, 3, 4
	**3**	a 1.47, m	38.7	2, 4	1, 2, 4, 5, 6
		b 1.44, m		2, 4	1, 2, 4, 5, 6
	**4**	1.63, m	24.1	3, 5, 6	2, 3, 5, 6
	**5**	0.89, d (*6.5*)	22.9	4	3, 4, 6
	**6**	0.82[d], d (*6.5*)	21.1	4	3, 4, 5
	**NH**	8.63, d (*6.0*)	---	2	2, 3, Val[4]-1
**L-Val[4]**	**1**	---	171.7	---	---
	**2**	4.10, dd (*8.6, 7.9*)	58.3	3, Val[4]-NH	1, 3, 4, 5
	**3**	1.87, m	29.8	2, 4, 5	1, 2, 4, 5
	**4**	0.88[c], d (*6.2*)	18.9	3	2, 3, 5
	**5**	0.81[d], d (*6.1*)	18.7	3	2, 3, 4
	**NH**	7.88, d (*7.9*)	---	2	2, 3, Gly[5]-1
**Gly[5]**	**1**	---	168.9	---	---
	**2**	a 4.07, dd (*16.6, 6.4*)	42.3	2b, Gly[5]-NH	1
		b 3.40, dd (*16.6, 4.1*)		2a, Gly[5]-NH	1
	**NH**	7.93, dd (*6.4, 4.1*)	---	2	2, 1′
**HMLA***	**1′**	---	169.8	---	---
	**2′**	a 2.48, dd (*14.9, 9.2*)	37.0	2′b, 3′	1′, 3′
		b 2.24, dd (*14.9, 1.8*)		2′a, 3′	1′, 3′, 4′
	**3′**	4.90, ddd (*9.2, 5.7, 1.8*)	75.1	2′b, 4′	Phe[1]-1, 1′, 4′-Me, 5′
	**4′**	1.50, m	36.1	3′, 4′-Me, 5′b	
	**5′**	a 1.19[b], m	31.4	4′, 5′b	
		b 0.87[c], m			4′-Me
	**6′**	a 1.19[b], m	26.5		
		b 1.09, m		5′b, 6′a	8′
	**7′**	1.18[b], m	29.3		9′
	**8′**	1.23[a], m	28.9		
	**9′**	1.23[a], m	28.7		7′, 10′, 11′
	**10′**	1.23[a], m	31.3		
	**11′**	1.25, m	22.1	12′	9′, 10′, 12′
	**12′**	0.85, t (*6.6*)	13.9	11′	10′, 11′
	**4′-Me**	0.67, d (*6.9*)	14.5	4′	3′, 4′, 5′

* HMLA: *3S*, *4S*-3-Hydroxy-4-methyllauric acid
(a – d) overlapping resonances within the same letter

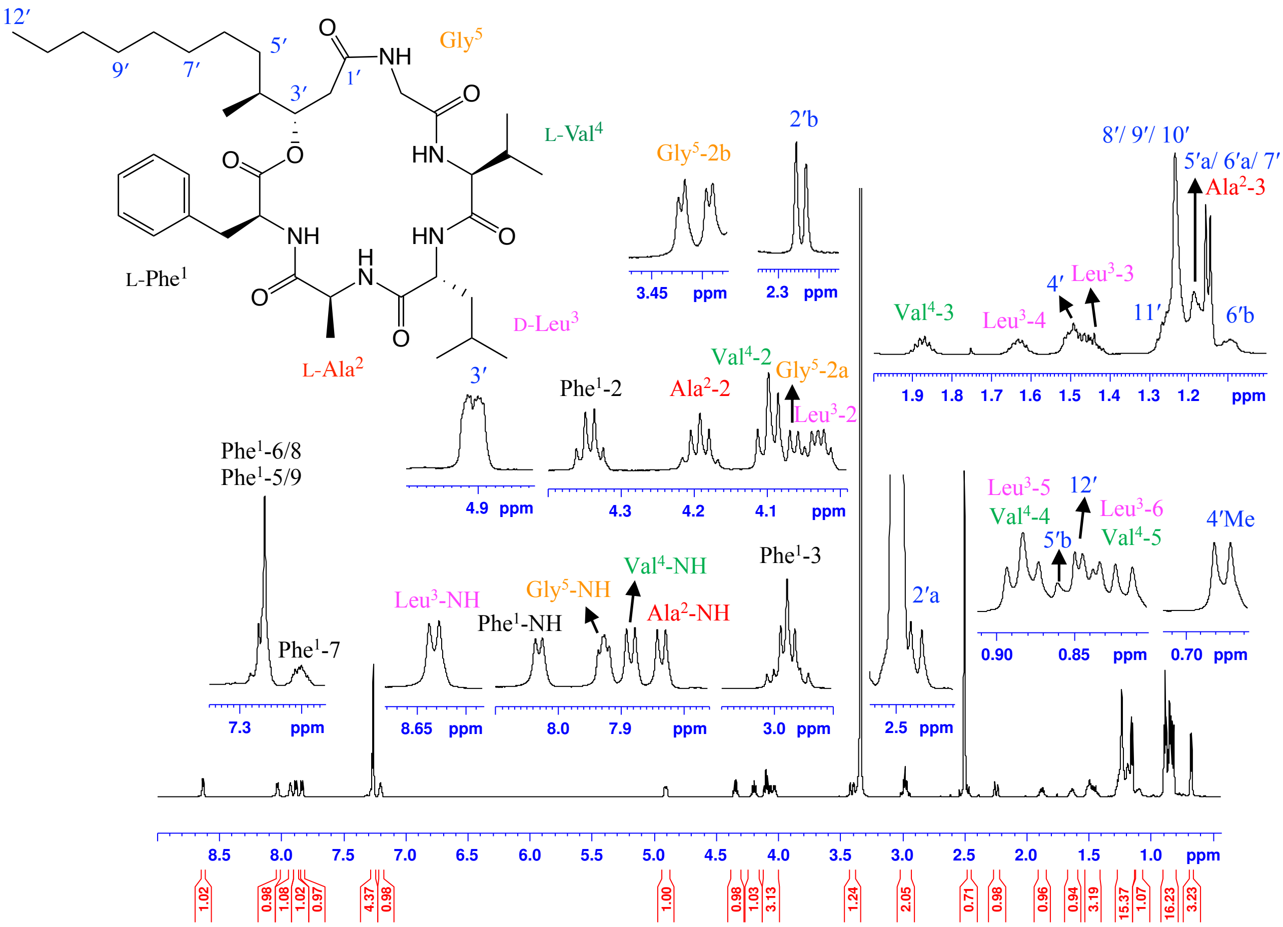

Figure S34. ^{1}H NMR (600 MHz, DMSO-d_6) spectrum for scopularide H (**8**)

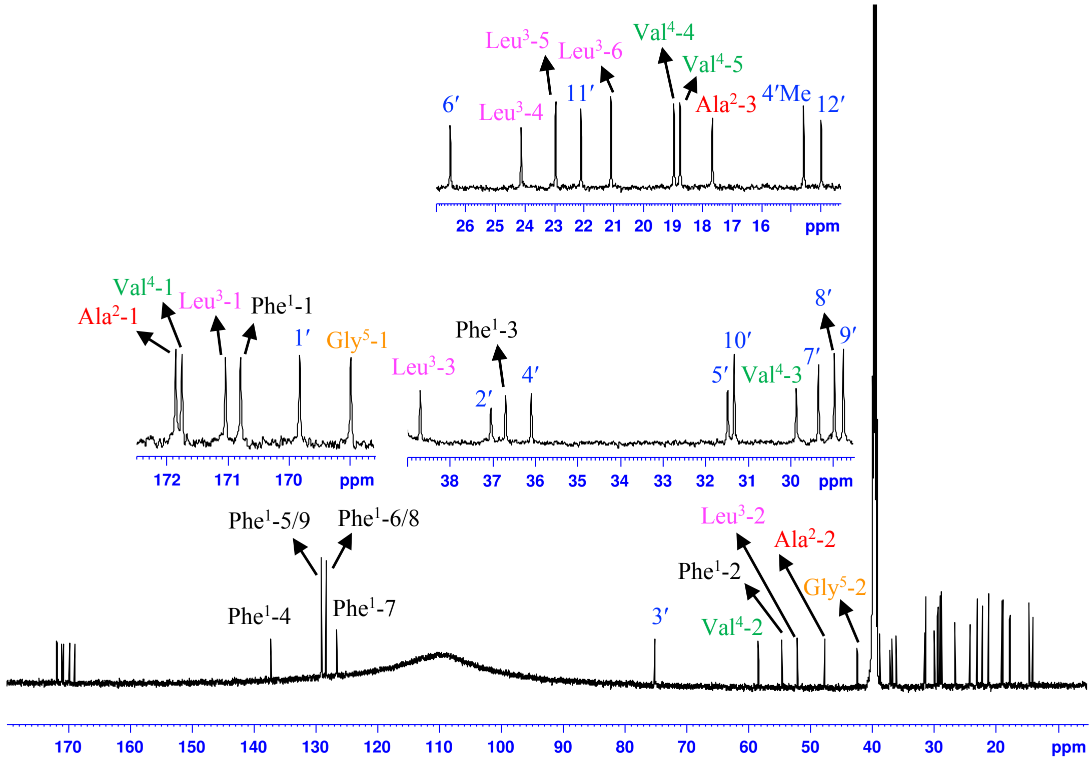

Figure S35. ^{13}C NMR (150 MHz, DMSO-d_6) spectrum for scopularide H (**8**)

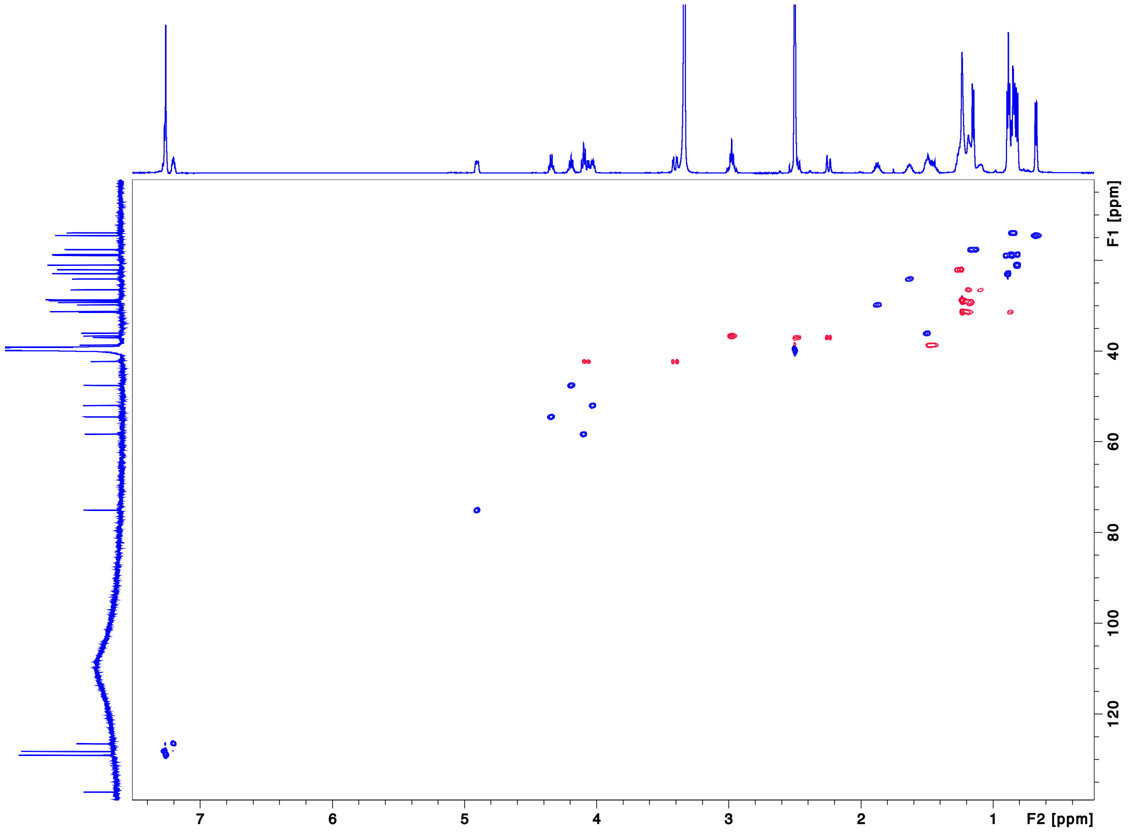

Figure S36. HSQC NMR (600 MHz, DMSO-d_6) spectrum for scopularide H (**8**)

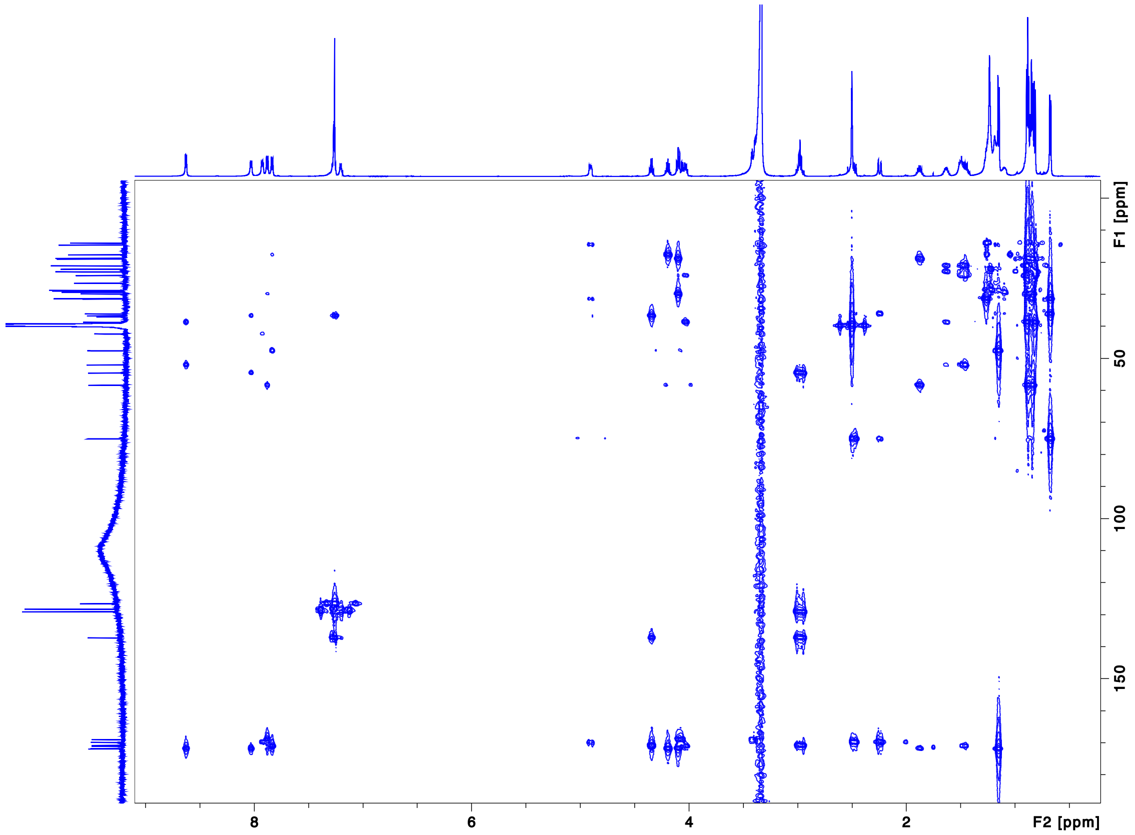

Figure S37. HMBC NMR (600 MHz, DMSO-d_6) spectrum for scopularide H (**8**)

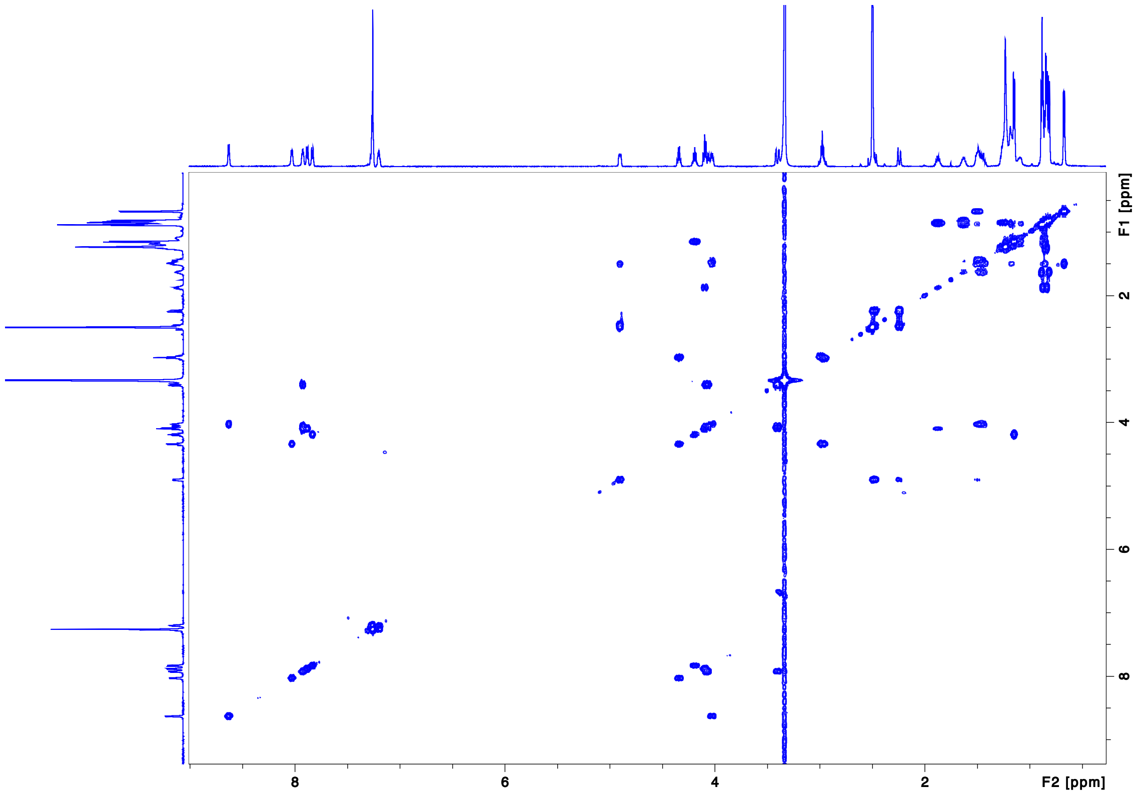

Figure S38. COSY NMR (600 MHz, DMSO-d_6) spectrum for scopularide H (**8**)

3 MS/MS fragmentation of scopularides C-H (3-8)

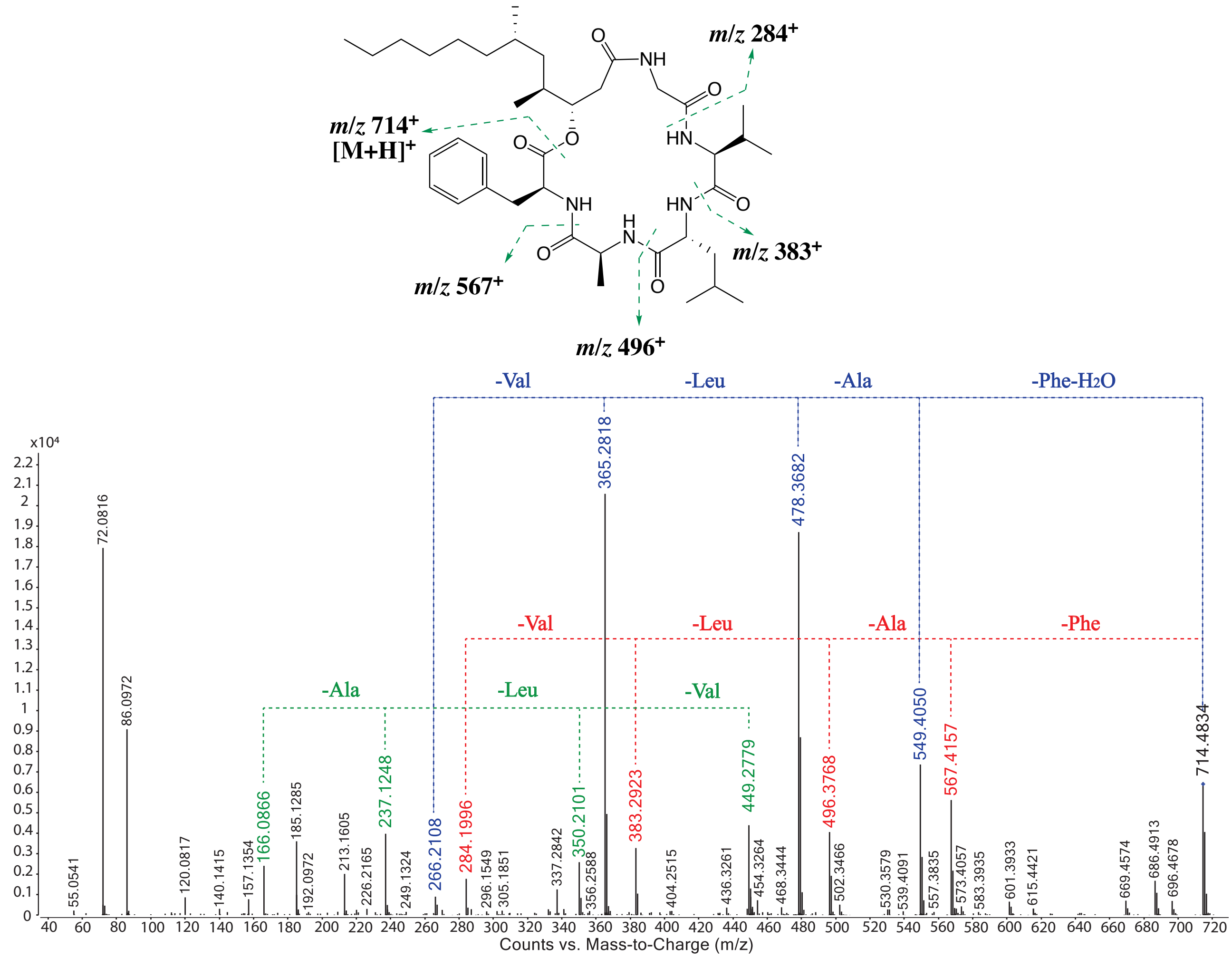

Figure S39. MS/MS fragmentation of scopularide C (**3**)

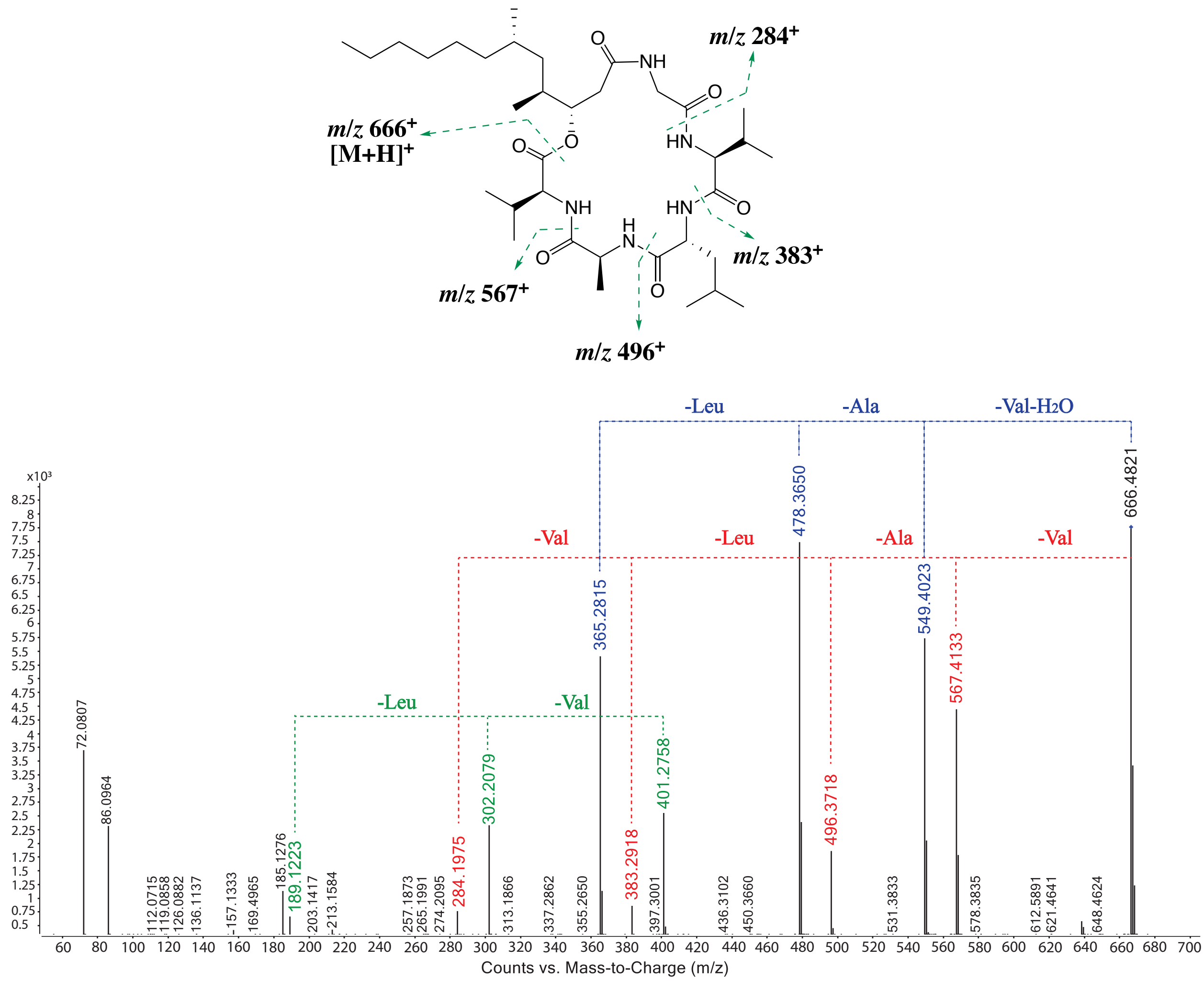

Figure S40. MS/MS fragmentation of scopularide D (**4**)

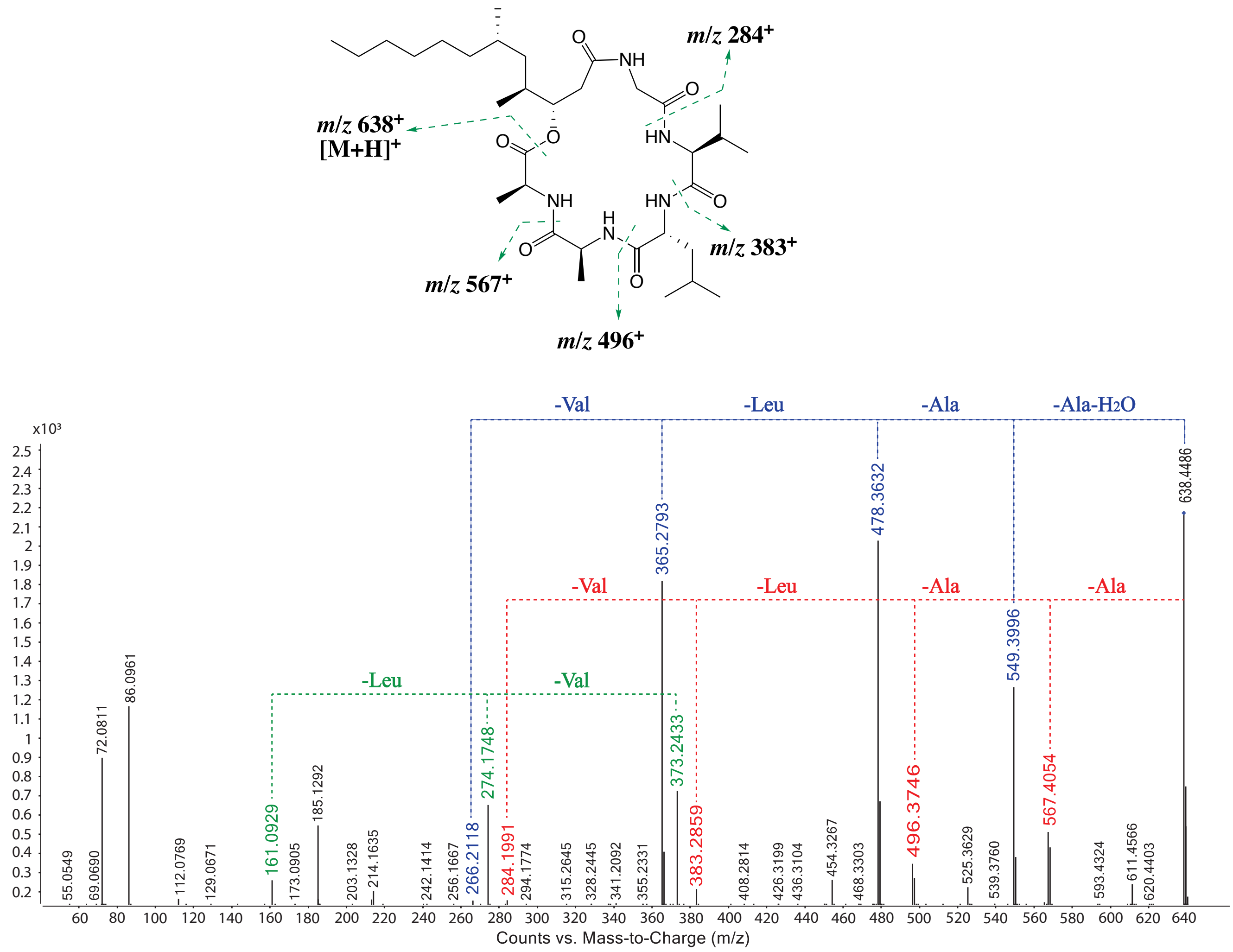

Figure S41. MS/MS fragmentation of scopularide E (**5**)

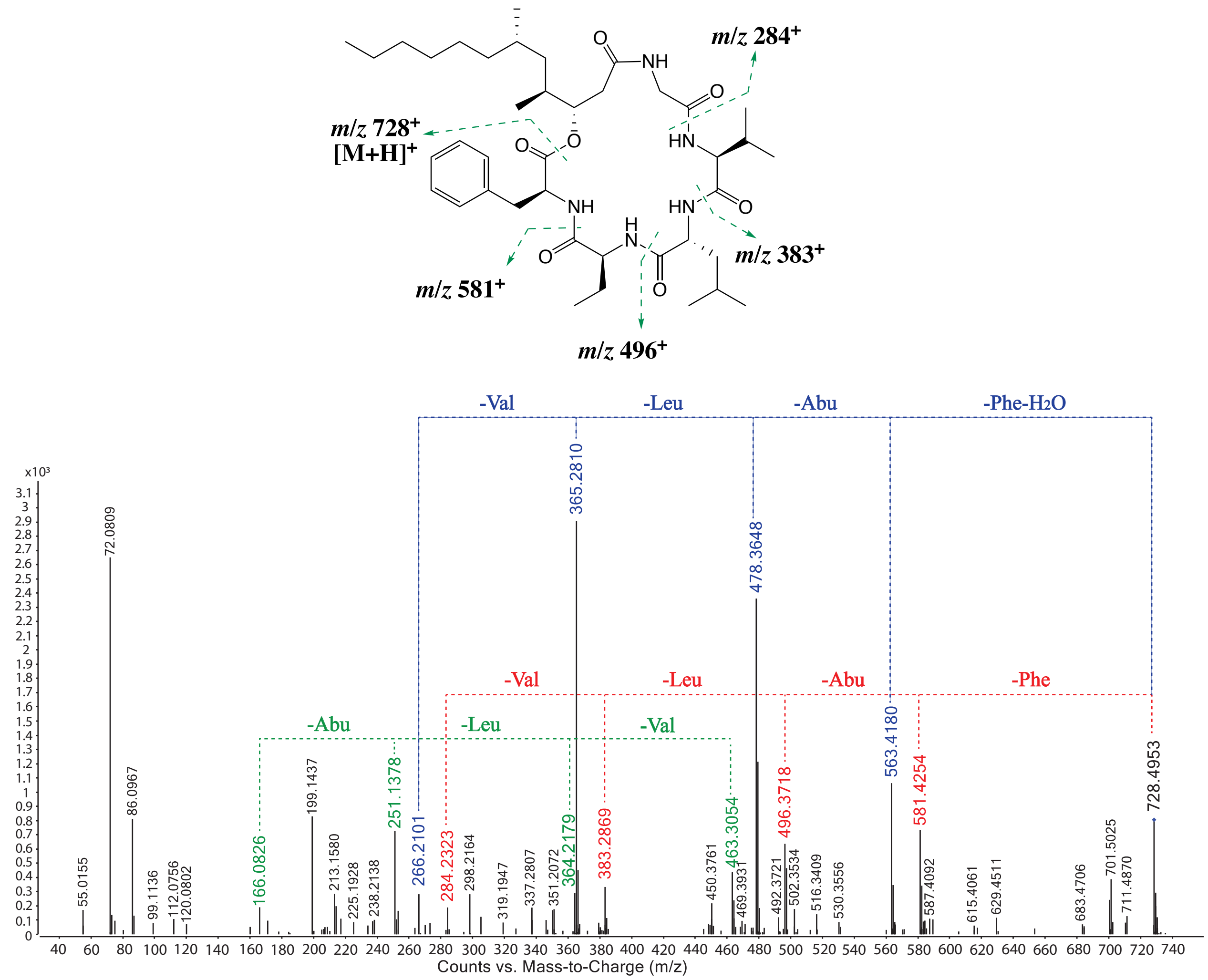

Figure S42. MS/MS fragmentation of scopularide F (**6**)

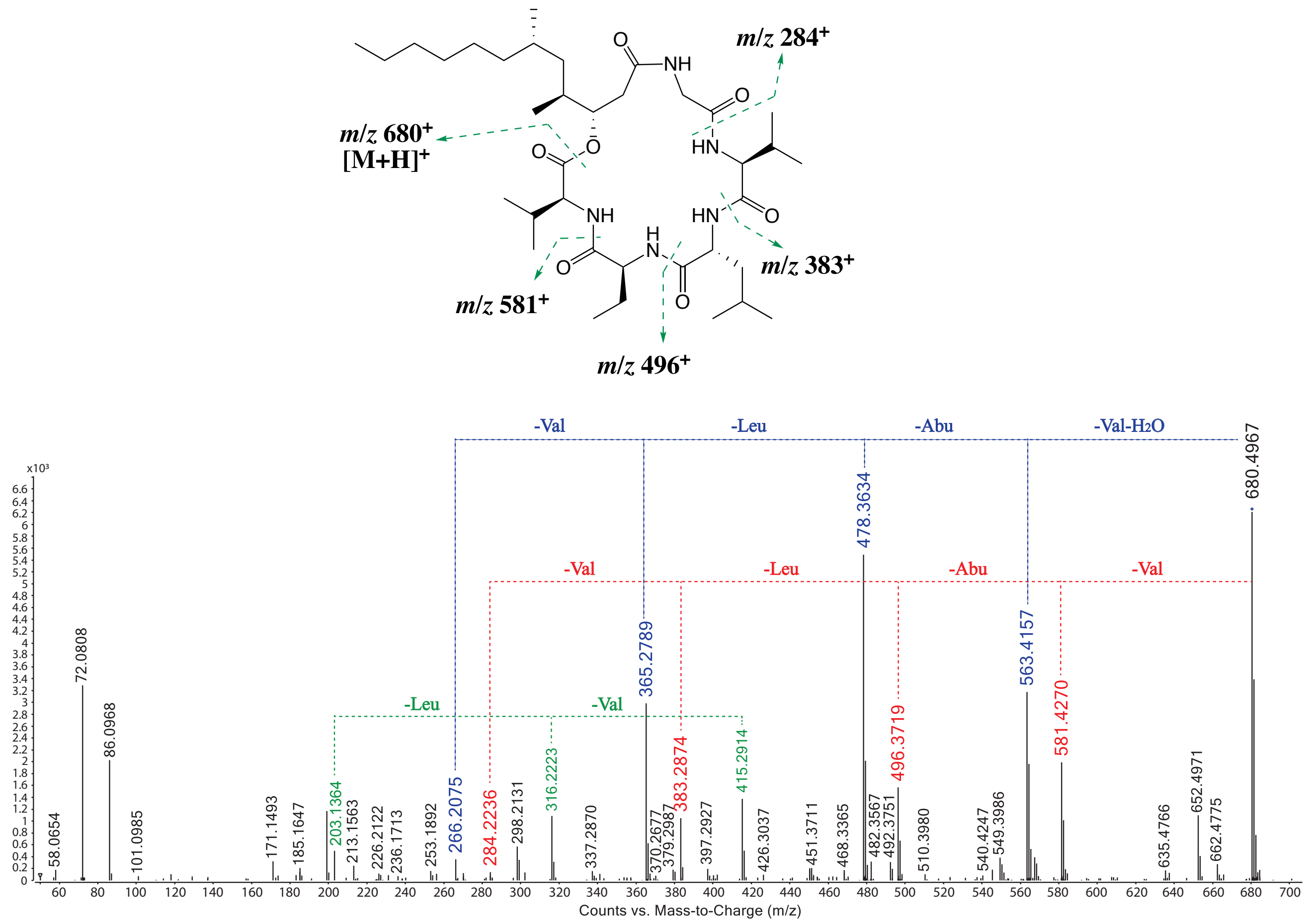

Figure S43. MS/MS fragmentation of scopularide G (**7**)

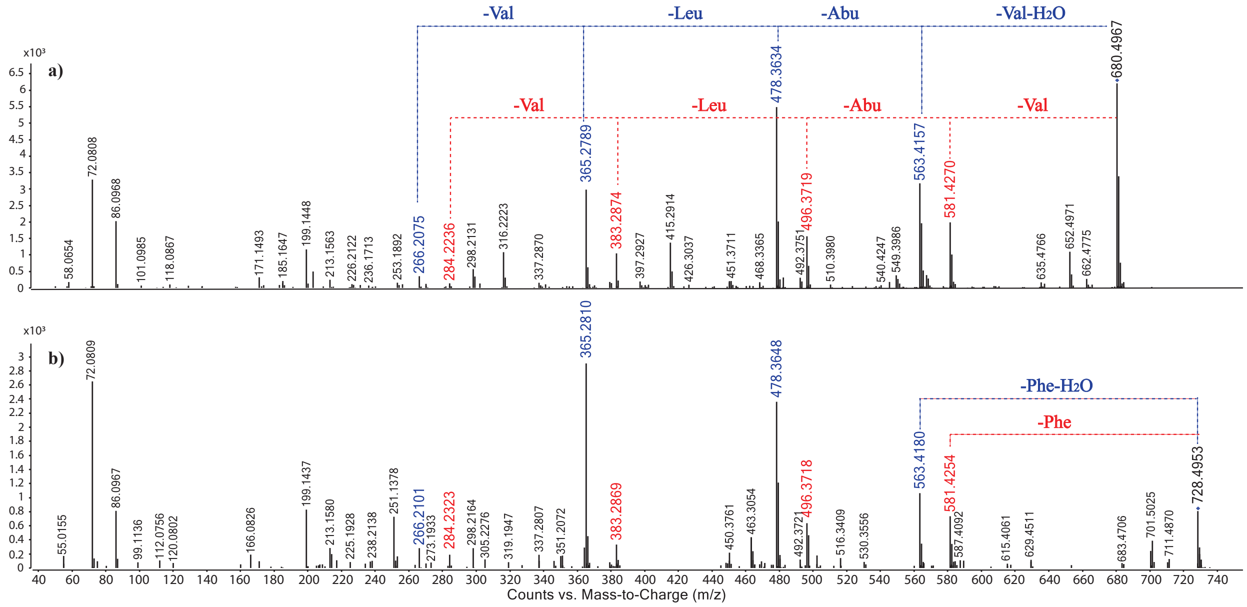

Figure S44. MS/MS fragmentation of a) scopularide G (**7**) compared to b) scopularide F (**6**)

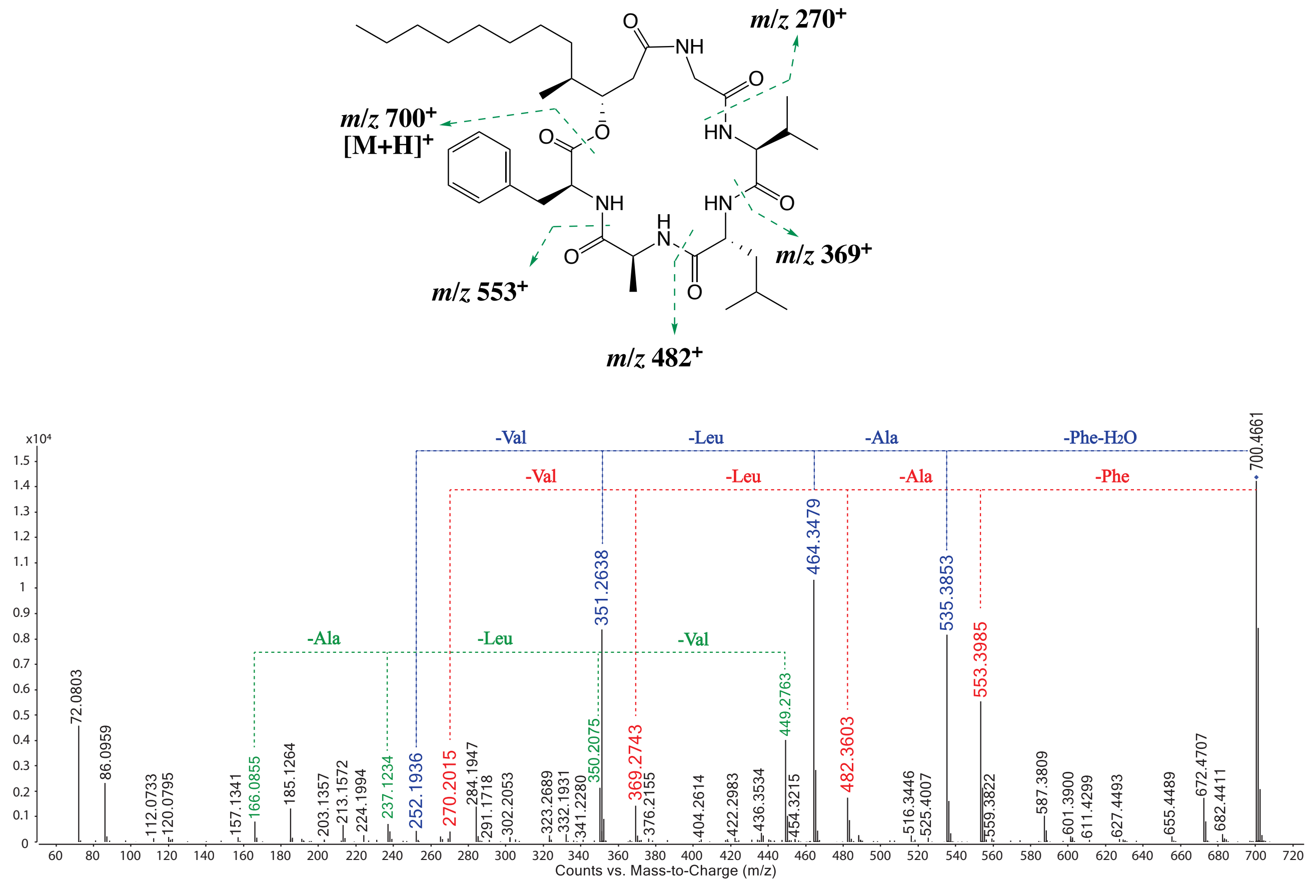

Figure S45. MS/MS fragmentation of scopularide H (**8**)

4 Marfey's analysis of scopularides A-H (1-8)[9]

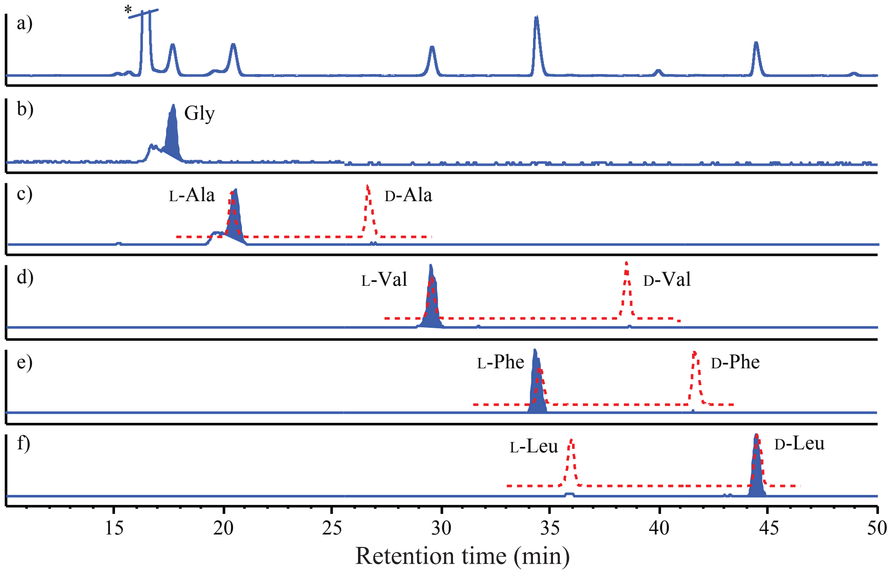

Figure S46. Marfey's analysis of scopularide A (**1**).

(**a**) C_3 HPLC-DAD (340 nm) chromatogram revealing L-FDAA amino acid derivatives. (**b-f**) C_3 HPLC-MS-SIE chromatograms for L-FDAA derivatives of authentic standards (red broken lines) and the acid hydrolysate of scopularide A (**1**) (blue shaded peaks). Traces confirm incorporates of (**b**) Gly (SIE m/z 328), (**c**) L-Ala (SIE *m/z* 342), (**d**) L-Val (SIE *m/z* 370), (**e**) L-Phe (SIE *m/z* 418) and (**f**) D-Leu (SIE *m/z* 384). * Excess Marfey's reagent.

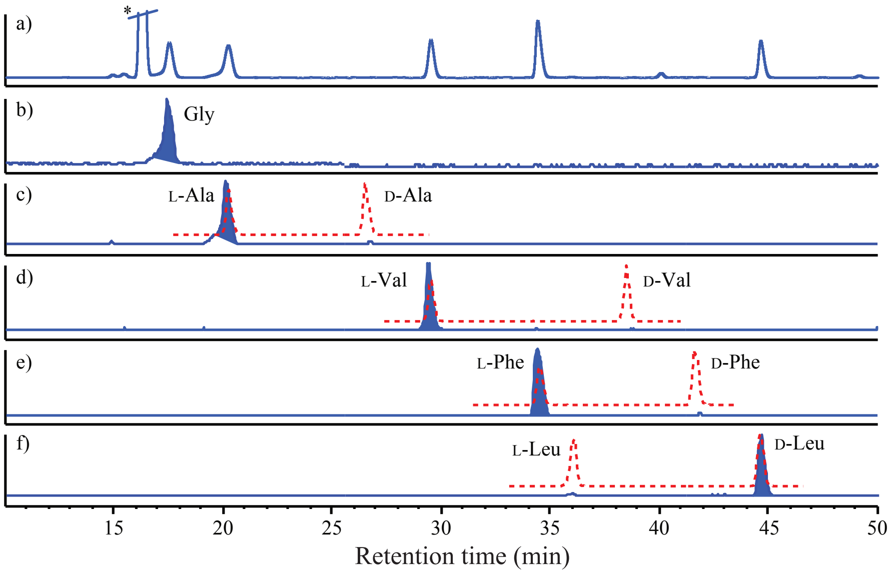

Figure S47. Marfey's analysis of scopularide B (**2**)

(**a**) C_3 HPLC-DAD (340 nm) chromatogram revealing L-FDAA amino acid derivatives. (**b-f**) C_3 HPLC-MS-SIE chromatograms for L-FDAA derivatives of authentic standards (red broken lines) and the acid hydrolysate of scopularide B (**2**) (blue shaded peaks). Traces confirm incorporates of (**b**) Gly (SIE m/z 328), (**c**) L-Ala (SIE *m/z* 342), (**d**) L-Val (SIE *m/z* 370), (**e**) L-Phe (SIE *m/z* 418) and (**f**) D-Leu (SIE *m/z* 384). * Excess Marfey's reagent.

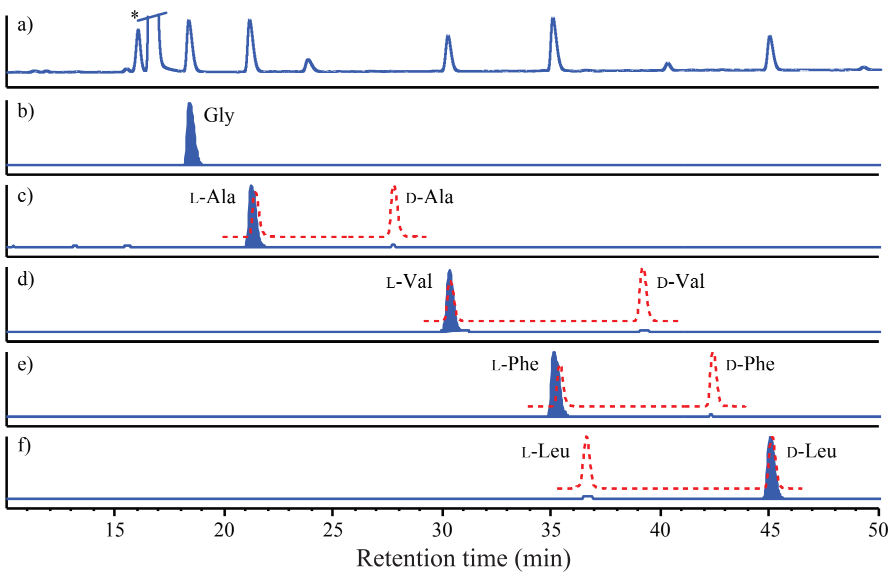

Figure S48. Marfey's analysis of scopularide C (**3**)

(**a**) C_3 HPLC-DAD (340 nm) chromatogram revealing L-FDAA amino acid derivatives. (**b-f**) C_3 HPLC-MS-SIE chromatograms for L-FDAA derivatives of authentic standards (red broken lines) and the acid hydrolysate of scopularide C (**3**) (blue shaded peaks). Traces confirm incorporates of (**b**) Gly (SIE m/z 328), (**c**) L-Ala (SIE *m/z* 342), (**d**) L-Val (SIE *m/z* 370), (**e**) L-Phe (SIE *m/z* 418) and (**f**) D-Leu (SIE *m/z* 384). * Excess Marfey's reagent.

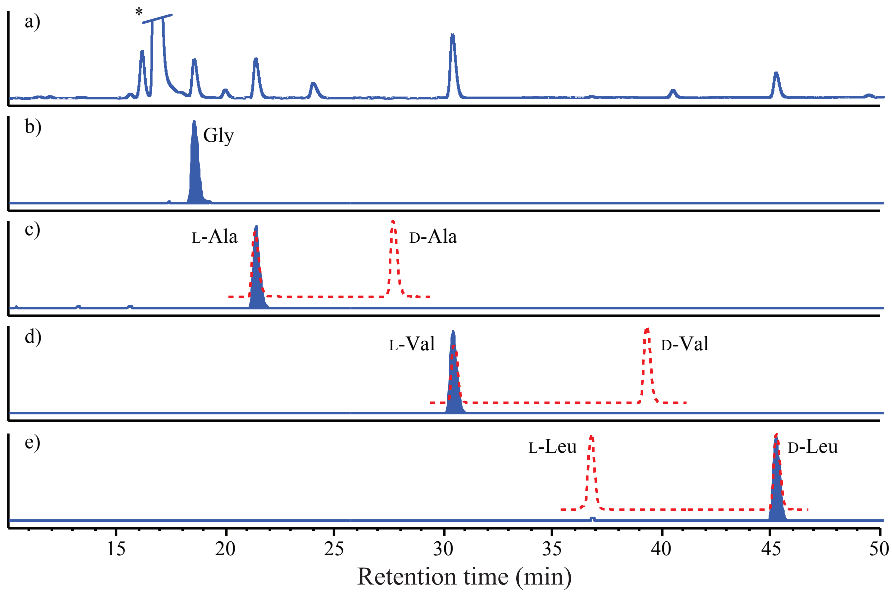

Figure S49. Marfey's analysis of scopularide D (**4**)

(**a**) C_3 HPLC-DAD (340 nm) chromatogram revealing L-FDAA amino acid derivatives. (**b-e**) C_3 HPLC-MS-SIE chromatograms for L-FDAA derivatives of authentic standards (red broken lines) and the acid hydrolysate of scopularide D (**4**) (blue shaded peaks). Traces confirm incorporates of (**b**) Gly (SIE m/z 328), (**c**) L-Ala (SIE *m/z* 342), (**d**) L-Val (SIE *m/z* 370) and (**e**) D-Leu (SIE *m/z* 384). * Excess Marfey's reagent.

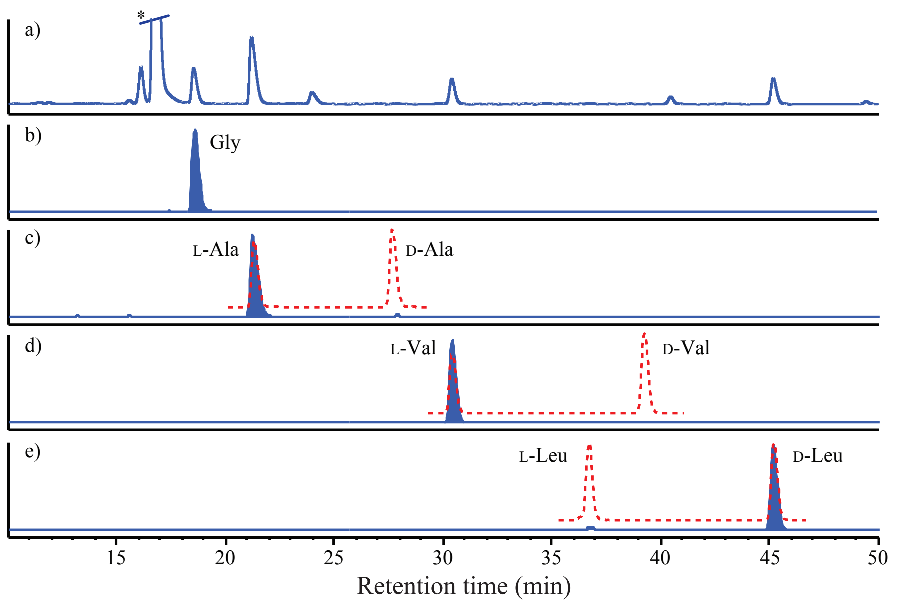

Figure S50. Marfey's analysis of scopularide E (**5**)

(**a**) C_3 HPLC-DAD (340 nm) chromatogram revealing L-FDAA amino acid derivatives. (**b-e**) C_3 HPLC-MS-SIE chromatograms for L-FDAA derivatives of authentic standards (red broken lines) and the acid hydrolysate of scopularide E (**5**) (blue shaded peaks). Traces confirm incorporates of (**b**) Gly (SIE m/z 328), (**c**) L-Ala (SIE *m/z* 342), (**d**) L-Val (SIE *m/z* 370) and (**e**) D-Leu (SIE *m/z* 384). * Excess Marfey's reagent.

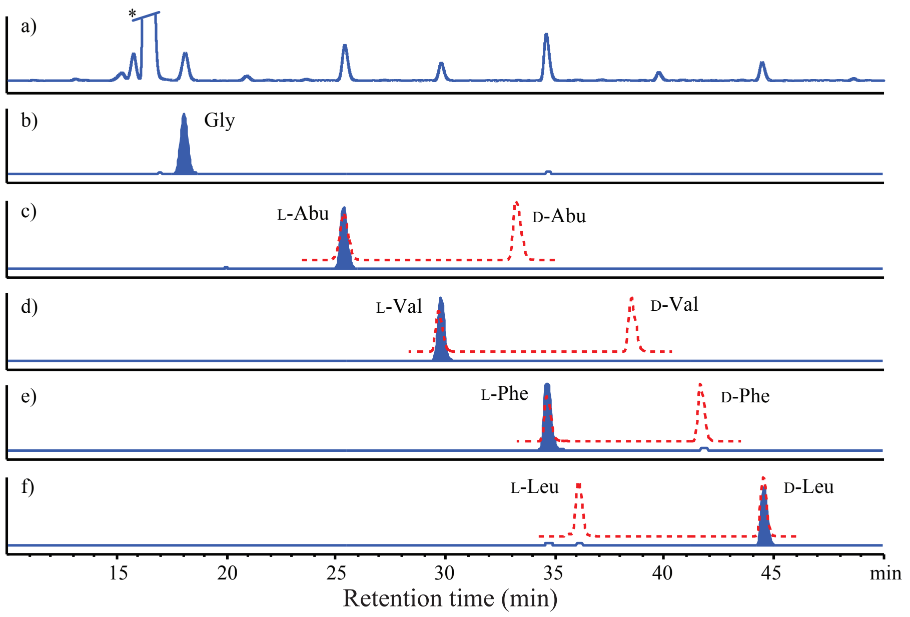

Figure S51. Marfey's analysis of scopularide F (**6**)

(**a**) C_3 HPLC-DAD (340 nm) chromatogram revealing L-FDAA amino acid derivatives. (**b-f**) C_3 HPLC-MS-SIE chromatograms for L-FDAA derivatives of authentic standards (red broken lines) and the acid hydrolysate of scopularide F (**6**) (blue shaded peaks). Traces confirm incorporates of (**b**) Gly (SIE m/z 328), (**c**) L-Abu (SIE *m/z* 356), (**d**) L-Val (SIE *m/z* 370), (**e**) L-Phe (SIE *m/z* 418) and (**f**) D-Leu (SIE *m/z* 384). * Excess Marfey's reagent.

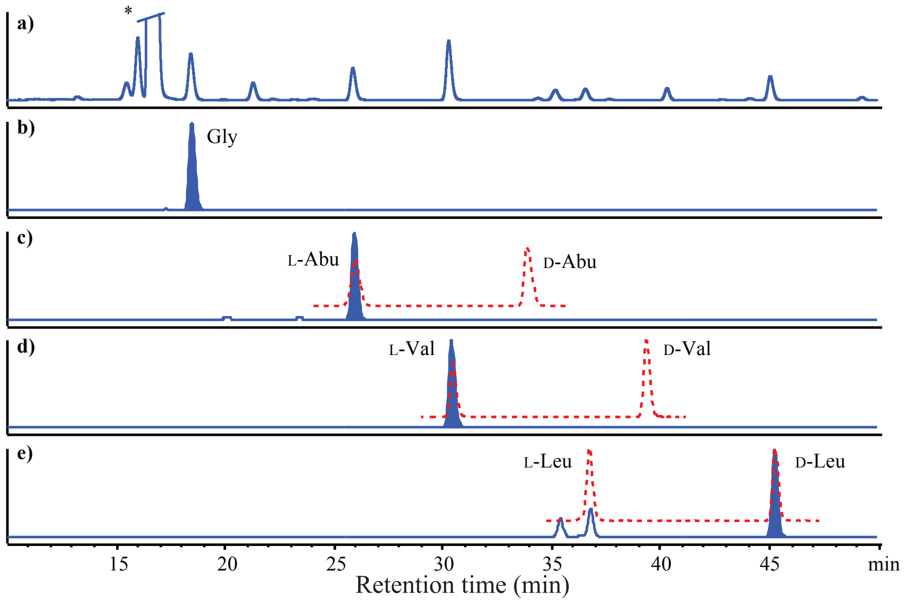

Figure S52. Marfey's analysis of scopularide G (**7**)

(**a**) C_3 HPLC-DAD (340 nm) chromatogram revealing L-FDAA amino acid derivatives. (**b-e**) C_3 HPLC-MS-SIE chromatograms for L-FDAA derivatives of authentic standards (red broken lines) and the acid hydrolysate of scopularide G (**7**) (blue shaded peaks). Traces confirm incorporates of (**b**) Gly (SIE m/z 328), (**c**) L-Abu (SIE *m/z* 356), (**d**) L-Val (SIE *m/z* 370) and (**e**) D-Leu (SIE *m/z* 384). * Excess Marfey's reagent.

5 GNPS molecular networking

5.1 GNPS networking of fish crude extract library

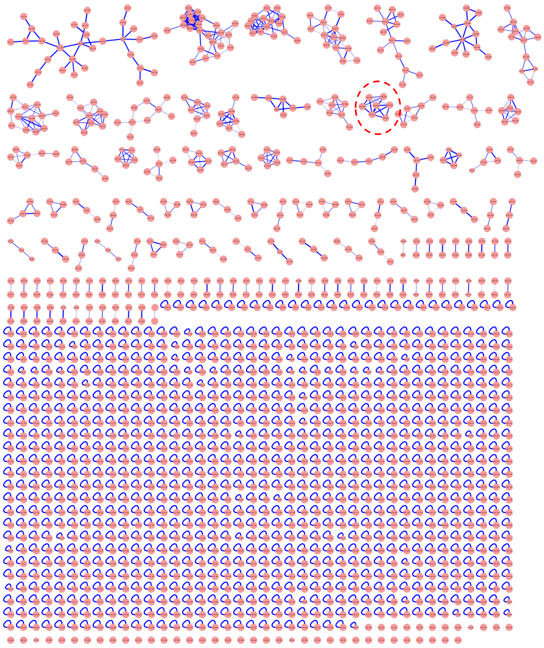

Figure S53. GNPS molecular network for a comparative analysis of ×63 Mugil mullet GIT-derived fungal extracts. Red circle represents GNPS cluster for scopularides detected in CMB-F458, CMB-F585 and CMB-F115.

5.2 GNPS networking of CMB-F458

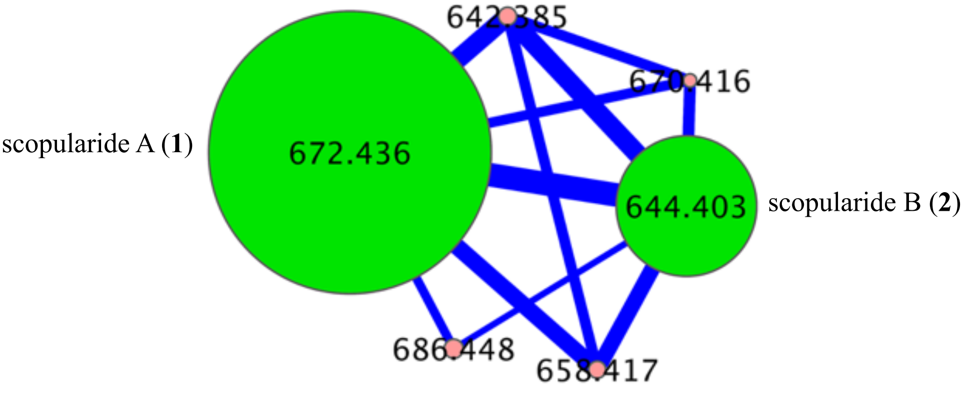

Figure S54. GNPS cluster for scopularides detected in a M1S solid phase cultivation of *Scopulariopsis* sp. CMB-F458. Green nodes are scopularides A-B (**1**-**2**), and pink nodes are minor analogues.

6 X-ray analysis of scopularide C (3) and scopularide H (8)

6.1 Scopularide C (3)

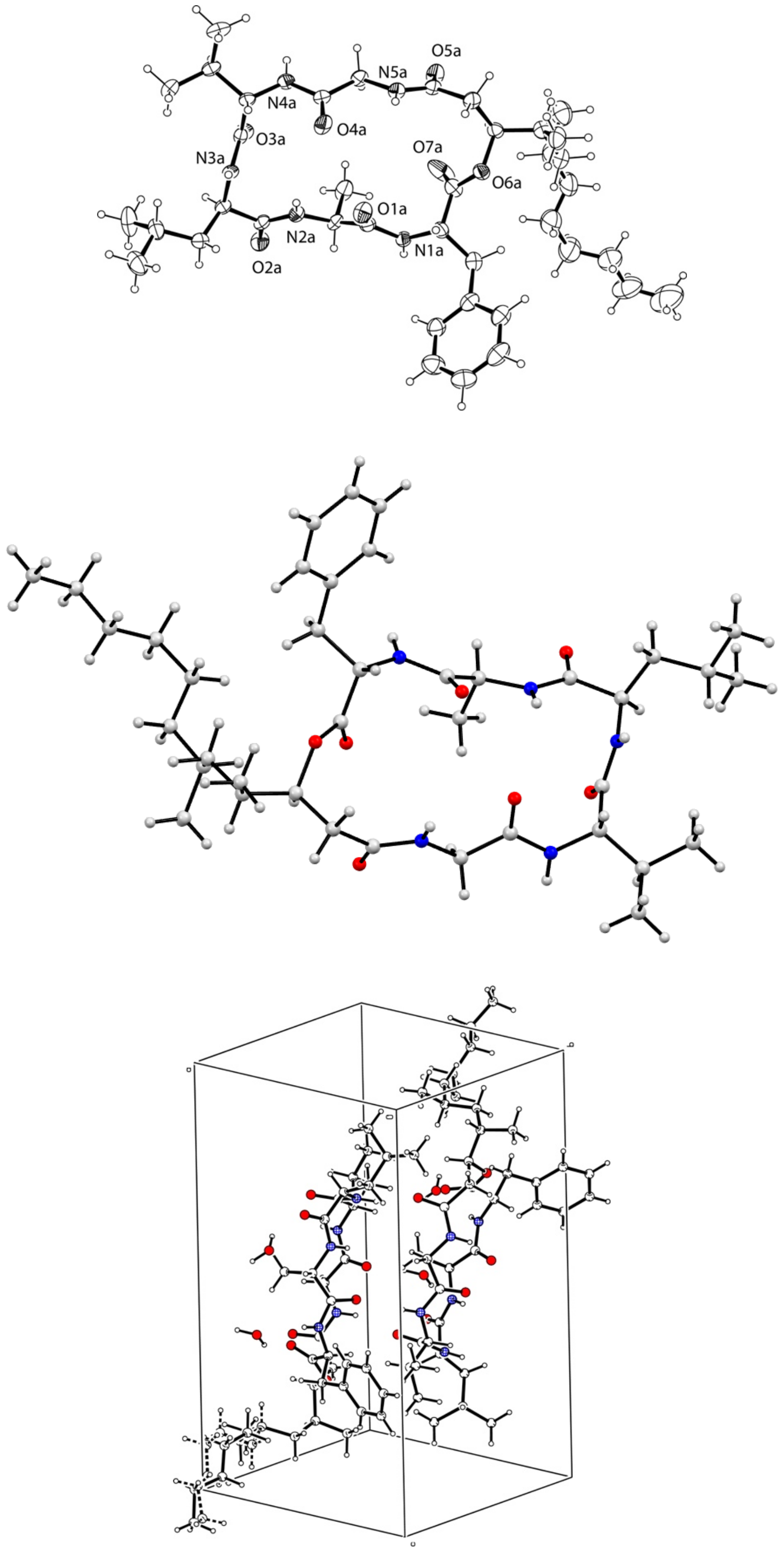

Figure S55. X-ray crystal structure of scopularide C **(3)**

Table S10. Crystal data and structure refinement for scopularide C (3)

Identification code	shelx	
Empirical formula	$C_{39} H_{63} N_5 O_7.H_2O$	
Formula weight	731.96	
Temperature	190(2) K	
Wavelength	1.54184 Å	
Crystal system	Triclinic	
Space group	*P* 1	
Unit cell dimensions	a = 9.2703(5) Å	α= 93.426(5)°.
	b = 13.5498(7) Å	β= 102.465(5)°.
	c = 19.0693(13) Å	γ = 109.998(5)°.
Volume	2174.4(2) $Å^3$	
Z	2	
Density (calculated)	1.118 Mg/m^3	
Absorption coefficient	0.630 mm^{-1}	
F(000)	796	
Crystal size	0.300 x 0.080 x 0.050 mm^3	
Theta range for data collection	3.952 to 61.358°.	
Index ranges	-10<=h<=10, -15<=k<=15, -21<=l<=21	
Reflections collected	19658	
Independent reflections	9235 [R(int) = 0.0654]	
Completeness to theta = 61.358°	98.9 %	
Absorption correction	Semi-empirical from equivalents	
Max. and min. transmission	1 and 0.728	
Refinement method	Full-matrix least-squares on F^2	
Data / restraints / parameters	9235 / 12 / 932	
Goodness-of-fit on F^2	1.054	
Final R indices [I>2sigma(I)]	R1 = 0.0667, wR2 = 0.1648	
R indices (all data)	R1 = 0.0865, wR2 = 0.1791	
Absolute structure parameter	0.2(2)	
Extinction coefficient	n/a	
Largest diff. peak and hole	0.362 and -0.284 e.$Å^{-3}$	

Bond lengths [Å] and angles [°] for scopularide C (3)

C(1A)-N(1A)	1.438(9)	C(1A)-C(13A)	1.510(9)
C(1A)-C(14A)	1.535(10)	C(2A)-O(1A)	1.218(8)
C(2A)-N(1A)	1.372(8)	C(2A)-C(3A)	1.508(10)
C(3A)-N(2A)	1.453(8)	C(3A)-C(21A)	1.505(9)
C(4A)-O(2A)	1.245(8)	C(4A)-N(2A)	1.322(8)
C(4A)-C(5A)	1.525(9)	C(5A)-N(3A)	1.458(8)
C(5A)-C(22A)	1.522(9)	C(6A)-O(3A)	1.214(7)
C(6A)-N(3A)	1.341(7)	C(6A)-C(7A)	1.531(9)
C(7A)-N(4A)	1.432(8)	C(7A)-C(26A)	1.545(8)
C(8A)-O(4A)	1.205(7)	C(8A)-N(4A)	1.348(8)
C(8A)-C(9A)	1.497(9)	C(9A)-N(5A)	1.444(8)
C(10A)-O(5A)	1.232(7)	C(10A)-N(5A)	1.328(8)
C(10A)-C(11A)	1.511(9)	C(11A)-C(12A)	1.515(10)
C(12A)-O(6A)	1.460(7)	C(12A)-C(29A)	1.510(10)
C(13A)-O(7A)	1.196(8)	C(13A)-O(6A)	1.330(8)
C(14A)-C(15A)	1.513(10)	C(15A)-C(20A)	1.381(11)
C(15A)-C(16A)	1.391(10)	C(16A)-C(17A)	1.369(13)
C(17A)-C(18A)	1.368(14)	C(18A)-C(19A)	1.368(14)
C(19A)-C(20A)	1.354(12)	C(22A)-C(23A)	1.517(11)
C(23A)-C(24A)	1.515(14)	C(23A)-C(25A)	1.531(12)
C(26A)-C(28A)	1.502(10)	C(26A)-C(27A)	1.520(10)
C(29A)-C(30A)	1.492(12)	C(29A)-C(31A)	1.536(13)
C(31A)-C(32A)	1.531(15)	C(32A)-C(33A)	1.520(15)
C(32A)-C(34A)	1.534(15)	C(34A)-C(35A)	1.426(16)
C(35A)-C(36A)	1.590(19)	C(36A)-C(37A)	1.45(2)
C(37A)-C(38A)	1.510(19)	C(38A)-C(39A)	1.47(3)
C(1B)-N(1B)	1.446(8)	C(1B)-C(13B)	1.519(10)
C(1B)-C(14B)	1.542(9)	C(2B)-O(1B)	1.229(7)
C(2B)-N(1B)	1.356(8)	C(2B)-C(3B)	1.499(9)
C(3B)-N(2B)	1.460(8)	C(3B)-C(21B)	1.523(10)
C(4B)-O(2B)	1.242(7)	C(4B)-N(2B)	1.315(8)
C(4B)-C(5B)	1.540(9)	C(5B)-N(3B)	1.460(8)
C(5B)-C(22B)	1.517(10)	C(6B)-O(3B)	1.219(6)
C(6B)-N(3B)	1.327(8)	C(6B)-C(7B)	1.529(8)
C(7B)-N(4B)	1.446(9)	C(7B)-C(26B)	1.538(9)
C(8B)-O(4B)	1.195(8)	C(8B)-N(4B)	1.338(8)
C(8B)-C(9B)	1.522(9)	C(9B)-N(5B)	1.450(8)

C(10B)-O(5B)	1.226(7)	C(10B)-N(5B)	1.321(8)
C(10B)-C(11B)	1.516(9)	C(11B)-C(12B)	1.505(9)
C(12B)-O(6B)	1.474(8)	C(12B)-C(29B)	1.551(10)
C(13B)-O(7B)	1.187(8)	C(13B)-O(6B)	1.337(8)
C(14B)-C(15B)	1.491(10)	C(15B)-C(16B)	1.383(11)
C(15B)-C(20B)	1.385(10)	C(16B)-C(17B)	1.362(13)
C(17B)-C(18B)	1.345(14)	C(18B)-C(19B)	1.362(14)
C(19B)-C(20B)	1.373(13)	C(22B)-C(23B)	1.536(11)
C(23B)-C(24B)	1.479(16)	C(23B)-C(25B)	1.525(16)
C(26B)-C(27B)	1.497(11)	C(26B)-C(28B)	1.529(10)
C(29B)-C(30B)	1.510(11)	C(29B)-C(31B)	1.546(11)
C(31B)-C(32B)	1.526(13)	C(32B)-C(34B)	1.485(17)
C(32B)-C(33B)	1.494(17)	C(32B)-C(34C)	1.71(2)
C(34B)-C(35B)	1.541(18)	C(35B)-C(36B)	1.55(2)
C(36B)-C(37B)	1.38(2)	C(37B)-C(38B)	1.66(2)
C(38B)-C(39B)	1.46(2)	C(34C)-C(35C)	1.541(18)
C(35C)-C(36C)	1.55(2)	C(36C)-C(37C)	1.38(2)
C(37C)-C(38C)	1.66(2)	C(38C)-C(39C)	1.46(2)
		N(1A)-C(1A)-C(13A)	108.2(5)
N(1A)-C(1A)-C(14A)	112.0(6)	C(13A)-C(1A)-C(14A)	112.2(6)
O(1A)-C(2A)-N(1A)	122.4(6)	O(1A)-C(2A)-C(3A)	123.4(6)
N(1A)-C(2A)-C(3A)	114.1(6)	N(2A)-C(3A)-C(21A)	109.8(5)
N(2A)-C(3A)-C(2A)	108.7(5)	C(21A)-C(3A)-C(2A)	111.6(6)
O(2A)-C(4A)-N(2A)	122.9(6)	O(2A)-C(4A)-C(5A)	118.9(6)
N(2A)-C(4A)-C(5A)	118.2(6)	N(3A)-C(5A)-C(22A)	110.8(5)
N(3A)-C(5A)-C(4A)	111.6(5)	C(22A)-C(5A)-C(4A)	110.7(5)
O(3A)-C(6A)-N(3A)	122.3(6)	O(3A)-C(6A)-C(7A)	121.0(5)
N(3A)-C(6A)-C(7A)	116.7(5)	N(4A)-C(7A)-C(6A)	108.3(5)
N(4A)-C(7A)-C(26A)	112.1(4)	C(6A)-C(7A)-C(26A)	110.2(5)
O(4A)-C(8A)-N(4A)	121.6(5)	O(4A)-C(8A)-C(9A)	123.3(5)
N(4A)-C(8A)-C(9A)	115.1(5)	N(5A)-C(9A)-C(8A)	111.3(4)
O(5A)-C(10A)-N(5A)	121.4(5)	O(5A)-C(10A)-C(11A)	119.0(5)
N(5A)-C(10A)-C(11A)	119.6(5)	C(10A)-C(11A)-C(12A)	111.2(5)
O(6A)-C(12A)-C(29A)	107.5(5)	O(6A)-C(12A)-C(11A)	107.6(5)
C(29A)-C(12A)-C(11A)	116.1(6)	O(7A)-C(13A)-O(6A)	123.1(6)
O(7A)-C(13A)-C(1A)	124.8(7)	O(6A)-C(13A)-C(1A)	112.0(5)
C(15A)-C(14A)-C(1A)	114.5(6)	C(20A)-C(15A)-C(16A)	116.7(7)
C(20A)-C(15A)-C(14A)	122.8(6)	C(16A)-C(15A)-C(14A)	120.5(7)

C(17A)-C(16A)-C(15A)	121.3(8)	C(18A)-C(17A)-C(16A)	120.7(8)
C(17A)-C(18A)-C(19A)	118.4(8)	C(20A)-C(19A)-C(18A)	121.3(9)
C(19A)-C(20A)-C(15A)	121.6(8)	C(23A)-C(22A)-C(5A)	113.8(6)
C(24A)-C(23A)-C(22A)	113.2(7)	C(24A)-C(23A)-C(25A)	111.6(7)
C(22A)-C(23A)-C(25A)	109.6(7)	C(28A)-C(26A)-C(27A)	112.4(6)
C(28A)-C(26A)-C(7A)	110.6(5)	C(27A)-C(26A)-C(7A)	109.6(6)
C(30A)-C(29A)-C(12A)	113.6(6)	C(30A)-C(29A)-C(31A)	111.0(7)
C(12A)-C(29A)-C(31A)	111.3(7)	C(32A)-C(31A)-C(29A)	118.5(7)
C(33A)-C(32A)-C(31A)	112.0(9)	C(33A)-C(32A)-C(34A)	108.2(9)
C(31A)-C(32A)-C(34A)	113.2(10)	C(35A)-C(34A)-C(32A)	115.5(10)
C(34A)-C(35A)-C(36A)	112.6(11)	C(37A)-C(36A)-C(35A)	114.0(12)
C(36A)-C(37A)-C(38A)	116.1(15)	C(39A)-C(38A)-C(37A)	110.6(17)
C(2A)-N(1A)-C(1A)	119.7(5)	C(4A)-N(2A)-C(3A)	123.9(5)
C(6A)-N(3A)-C(5A)	119.8(5)	C(8A)-N(4A)-C(7A)	121.3(4)
C(10A)-N(5A)-C(9A)	121.2(4)	C(13A)-O(6A)-C(12A)	118.2(5)
N(1B)-C(1B)-C(13B)	108.4(5)	N(1B)-C(1B)-C(14B)	111.7(5)
C(13B)-C(1B)-C(14B)	112.5(6)	O(1B)-C(2B)-N(1B)	122.2(6)
O(1B)-C(2B)-C(3B)	123.3(6)	N(1B)-C(2B)-C(3B)	114.4(5)
N(2B)-C(3B)-C(2B)	108.9(5)	N(2B)-C(3B)-C(21B)	108.9(6)
C(2B)-C(3B)-C(21B)	111.3(6)	O(2B)-C(4B)-N(2B)	123.6(6)
O(2B)-C(4B)-C(5B)	118.5(6)	N(2B)-C(4B)-C(5B)	117.9(5)
N(3B)-C(5B)-C(22B)	110.5(5)	N(3B)-C(5B)-C(4B)	110.5(5)
C(22B)-C(5B)-C(4B)	110.2(5)	O(3B)-C(6B)-N(3B)	123.0(5)
O(3B)-C(6B)-C(7B)	120.7(5)	N(3B)-C(6B)-C(7B)	116.3(4)
N(4B)-C(7B)-C(6B)	109.0(5)	N(4B)-C(7B)-C(26B)	111.1(5)
C(6B)-C(7B)-C(26B)	110.6(5)	O(4B)-C(8B)-N(4B)	123.4(6)
O(4B)-C(8B)-C(9B)	122.5(5)	N(4B)-C(8B)-C(9B)	114.1(5)
N(5B)-C(9B)-C(8B)	110.9(5)	O(5B)-C(10B)-N(5B)	121.6(5)
O(5B)-C(10B)-C(11B)	119.3(6)	N(5B)-C(10B)-C(11B)	119.0(5)
C(12B)-C(11B)-C(10B)	110.4(5)	O(6B)-C(12B)-C(11B)	108.5(5)
O(6B)-C(12B)-C(29B)	106.3(5)	C(11B)-C(12B)-C(29B)	114.5(5)
O(7B)-C(13B)-O(6B)	124.0(6)	O(7B)-C(13B)-C(1B)	125.4(6)
O(6B)-C(13B)-C(1B)	110.6(5)	C(15B)-C(14B)-C(1B)	115.8(5)
C(16B)-C(15B)-C(20B)	116.0(7)	C(16B)-C(15B)-C(14B)	122.3(6)
C(20B)-C(15B)-C(14B)	121.8(7)	C(17B)-C(16B)-C(15B)	120.3(8)
C(18B)-C(17B)-C(16B)	122.5(10)	C(17B)-C(18B)-C(19B)	119.3(9)
C(18B)-C(19B)-C(20B)	118.6(8)	C(19B)-C(20B)-C(15B)	123.2(8)
C(5B)-C(22B)-C(23B)	115.1(7)	C(24B)-C(23B)-C(25B)	112.4(10)

C(24B)-C(23B)-C(22B)	113.3(8)	C(25B)-C(23B)-C(22B)	109.6(8)
C(27B)-C(26B)-C(28B)	110.6(6)	C(27B)-C(26B)-C(7B)	111.2(6)
C(28B)-C(26B)-C(7B)	110.3(6)	C(30B)-C(29B)-C(31B)	111.1(7)
C(30B)-C(29B)-C(12B)	112.8(6)	C(31B)-C(29B)-C(12B)	110.0(6)
C(32B)-C(31B)-C(29B)	117.0(7)	C(34B)-C(32B)-C(31B)	120.8(11)
C(33B)-C(32B)-C(31B)	112.5(8)	C(33B)-C(32B)-C(34C)	124.0(11)
C(31B)-C(32B)-C(34C)	98.0(9)	C(32B)-C(34B)-C(35B)	112.2(13)
C(34B)-C(35B)-C(36B)	114.0(12)	C(37B)-C(36B)-C(35B)	116.2(13)
C(36B)-C(37B)-C(38B)	114.8(13)	C(39B)-C(38B)-C(37B)	110.2(14)
C(35C)-C(34C)-C(32B)	104.3(15)	C(34C)-C(35C)-C(36C)	114.1(14)
C(37C)-C(36C)-C(35C)	117.3(15)	C(36C)-C(37C)-C(38C)	113.0(14)
C(39C)-C(38C)-C(37C)	109.9(16)	C(2B)-N(1B)-C(1B)	120.4(5)
C(4B)-N(2B)-C(3B)	124.4(5)	C(6B)-N(3B)-C(5B)	119.5(4)
C(8B)-N(4B)-C(7B)	121.2(5)	C(10B)-N(5B)-C(9B)	120.5(5)
C(13B)-O(6B)-C(12B)	116.7(5)		

6.2 Scopularide H (8)

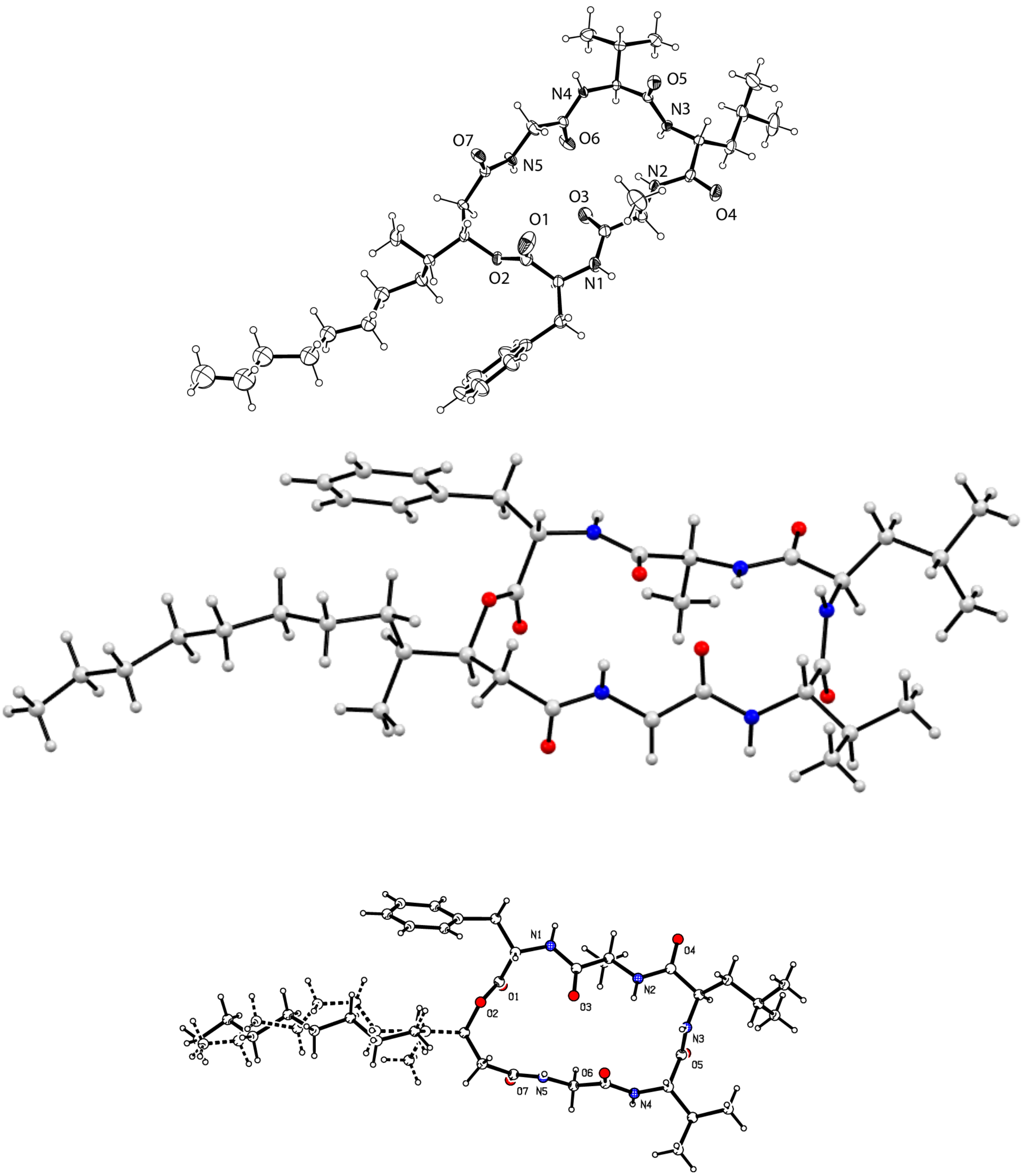

Figure S56. X-ray crystal structure of scopularide H **(8)**

Table S11. Crystal data and structure refinement for scopularide H (8)

Identification code	shelx	
Empirical formula	$C_{38} H_{61} N_5 O_7$	
Formula weight	699.91	
Temperature	190(2) K	
Wavelength	1.54184 Å	
Crystal system	Monoclinic	
Space group	*I* 2	
Unit cell dimensions	a = 18.6272(10) Å	α= 90°.
	b = 9.3679(5) Å	β= 111.432(6)°.
	c = 24.5547(12) Å	γ = 90°.
Volume	3988.5(4) Å^3	
Z	4	
Density (calculated)	1.166 Mg/m^3	
Absorption coefficient	0.647 mm^{-1}	
F(000)	1520	
Crystal size	0.400 x 0.015 x 0.015 mm^3	
Theta range for data collection	3.720 to 61.651°.	
Index ranges	-21<=h<=14, -10<=k<=10, -28<=l<=26	
Reflections collected	11831	
Independent reflections	5392 [R(int) = 0.0860]	
Completeness to theta = 61.651°	99.0 %	
Absorption correction	Semi-empirical from equivalents	
Max. and min. transmission	1 and 0.896	
Refinement method	Full-matrix least-squares on F^2	
Data / restraints / parameters	5392 / 18 / 445	
Goodness-of-fit on F^2	1.042	
Final R indices [I>2sigma(I)]	R1 = 0.0604, wR2 = 0.1394	
R indices (all data)	R1 = 0.0779, wR2 = 0.1545	
Absolute structure parameter	0.0(3)	
Extinction coefficient	n/a	
Largest diff. peak and hole	0.363 and -0.245 e.Å^{-3}	

Bond lengths [Å] and angles [°] for scopularide H (8)

C(1)-O(1)	1.191(7)	C(1)-O(2)	1.335(6)
C(1)-C(2)	1.512(8)	C(2)-N(1)	1.453(7)
C(2)-C(14)	1.537(7)	C(3)-O(3)	1.227(6)
C(3)-N(1)	1.352(7)	C(3)-C(4)	1.514(8)
C(4)-N(2)	1.452(6)	C(4)-C(21)	1.510(11)
C(5)-O(4)	1.243(6)	C(5)-N(2)	1.332(6)
C(5)-C(6)	1.522(7)	C(6)-N(3)	1.456(6)
C(6)-C(22)	1.514(7)	C(7)-O(5)	1.234(6)
C(7)-N(3)	1.348(6)	C(7)-C(8)	1.514(7)
C(8)-N(4)	1.449(6)	C(8)-C(26)	1.527(6)
C(9)-O(6)	1.214(7)	C(9)-N(4)	1.343(6)
C(9)-C(10)	1.503(7)	C(10)-N(5)	1.443(7)
C(11)-O(7)	1.229(6)	C(11)-N(5)	1.320(6)
C(11)-C(12)	1.513(7)	C(12)-C(13)	1.531(7)
C(13)-O(2)	1.460(6)	C(13)-C(29')	1.527(7)
C(13)-C(29)	1.527(7)	C(14)-C(15)	1.495(8)
C(15)-C(16)	1.381(9)	C(15)-C(20)	1.393(9)
C(16)-C(17)	1.387(10)	C(17)-C(18)	1.391(12)
C(18)-C(19)	1.370(11)	C(19)-C(20)	1.371(9)
C(22)-C(23)	1.531(8)	C(23)-C(24)	1.495(12)
C(23)-C(25)	1.531(10)	C(26)-C(28)	1.515(8)
C(26)-C(27)	1.525(7)	C(29)-C(30)	1.519(8)
C(29)-C(31)	1.530(8)	C(31)-C(32)	1.544(9)
C(32)-C(33)	1.507(13)	C(33)-C(34)	1.489(13)
C(34)-C(35)	1.518(15)	C(35)-C(36)	1.618(15)
C(36)-C(37)	1.513(18)	C(37)-C(38)	1.395(17)
C(29')-C(30')	1.519(8)	C(29')-C(31')	1.530(8)
C(31')-C(32')	1.545(9)	C(32')-C(33')	1.507(13)
C(33')-C(34')	1.489(13)	C(34')-C(35')	1.517(15)
C(35')-C(36')	1.621(15)	C(36')-C(37')	1.513(18)
C(37')-C(38')	1.394(17)		
O(1)-C(1)-O(2)	124.7(5)	O(1)-C(1)-C(2)	123.9(5)
O(2)-C(1)-C(2)	111.4(5)	N(1)-C(2)-C(1)	111.8(5)
N(1)-C(2)-C(14)	105.5(4)	C(1)-C(2)-C(14)	110.9(4)
O(3)-C(3)-N(1)	123.8(5)	O(3)-C(3)-C(4)	123.0(5)
N(1)-C(3)-C(4)	113.1(4)	N(2)-C(4)-C(21)	111.0(5)
N(2)-C(4)-C(3)	110.1(4)	C(21)-C(4)-C(3)	110.7(5)

O(4)-C(5)-N(2)	121.4(5)	O(4)-C(5)-C(6)	119.9(4)
N(2)-C(5)-C(6)	118.7(4)	N(3)-C(6)-C(22)	111.4(4)
N(3)-C(6)-C(5)	113.1(4)	C(22)-C(6)-C(5)	111.8(4)
O(5)-C(7)-N(3)	122.7(4)	O(5)-C(7)-C(8)	121.7(4)
N(3)-C(7)-C(8)	115.5(4)	N(4)-C(8)-C(7)	105.8(4)
N(4)-C(8)-C(26)	112.1(4)	C(7)-C(8)-C(26)	111.9(4)
O(6)-C(9)-N(4)	123.8(5)	O(6)-C(9)-C(10)	122.5(5)
N(4)-C(9)-C(10)	113.7(4)	N(5)-C(10)-C(9)	110.9(4)
O(7)-C(11)-N(5)	122.4(5)	O(7)-C(11)-C(12)	119.8(5)
N(5)-C(11)-C(12)	117.8(5)	C(11)-C(12)-C(13)	110.8(4)
O(2)-C(13)-C(29')	109.7(4)	O(2)-C(13)-C(29)	109.7(4)
O(2)-C(13)-C(12)	106.5(4)	C(29')-C(13)-C(12)	114.6(4)
C(29)-C(13)-C(12)	114.6(4)	C(15)-C(14)-C(2)	117.3(4)
C(16)-C(15)-C(20)	117.3(6)	C(16)-C(15)-C(14)	122.9(5)
C(20)-C(15)-C(14)	119.7(5)	C(15)-C(16)-C(17)	121.2(7)
C(16)-C(17)-C(18)	119.9(7)	C(19)-C(18)-C(17)	119.5(7)
C(18)-C(19)-C(20)	119.9(7)	C(19)-C(20)-C(15)	122.2(7)
C(6)-C(22)-C(23)	115.3(5)	C(24)-C(23)-C(22)	113.1(6)
C(24)-C(23)-C(25)	111.9(6)	C(22)-C(23)-C(25)	108.3(5)
C(28)-C(26)-C(27)	111.0(5)	C(28)-C(26)-C(8)	110.0(4)
C(27)-C(26)-C(8)	110.4(4)	C(30)-C(29)-C(13)	108.3(4)
C(30)-C(29)-C(31)	113.0(5)	C(13)-C(29)-C(31)	113.8(4)
C(29)-C(31)-C(32)	115.4(5)	C(33)-C(32)-C(31)	116.0(8)
C(34)-C(33)-C(32)	114.4(8)	C(33)-C(34)-C(35)	115.9(8)
C(34)-C(35)-C(36)	112.0(9)	C(37)-C(36)-C(35)	109.6(10)
C(38)-C(37)-C(36)	111.0(15)	C(30')-C(29')-C(13)	108.3(4)
C(30')-C(29')-C(31')	113.0(5)	C(13)-C(29')-C(31')	113.8(4)
C(29')-C(31')-C(32')	115.1(6)	C(33')-C(32')-C(31')	116.6(8)
C(34')-C(33')-C(32')	114.2(9)	C(33')-C(34')-C(35')	116.4(9)
C(34')-C(35')-C(36')	111.5(10)	C(37')-C(36')-C(35')	109.0(10)
C(38')-C(37')-C(36')	110.9(16)	C(3)-N(1)-C(2)	123.1(4)
C(5)-N(2)-C(4)	122.8(4)	C(7)-N(3)-C(6)	121.3(4)
C(9)-N(4)-C(8)	122.3(4)	C(11)-N(5)-C(10)	118.8(4)
C(1)-O(2)-C(13)	118.7(4)		

7 Biological assays

7.1 Antibacterial and antifungal assays

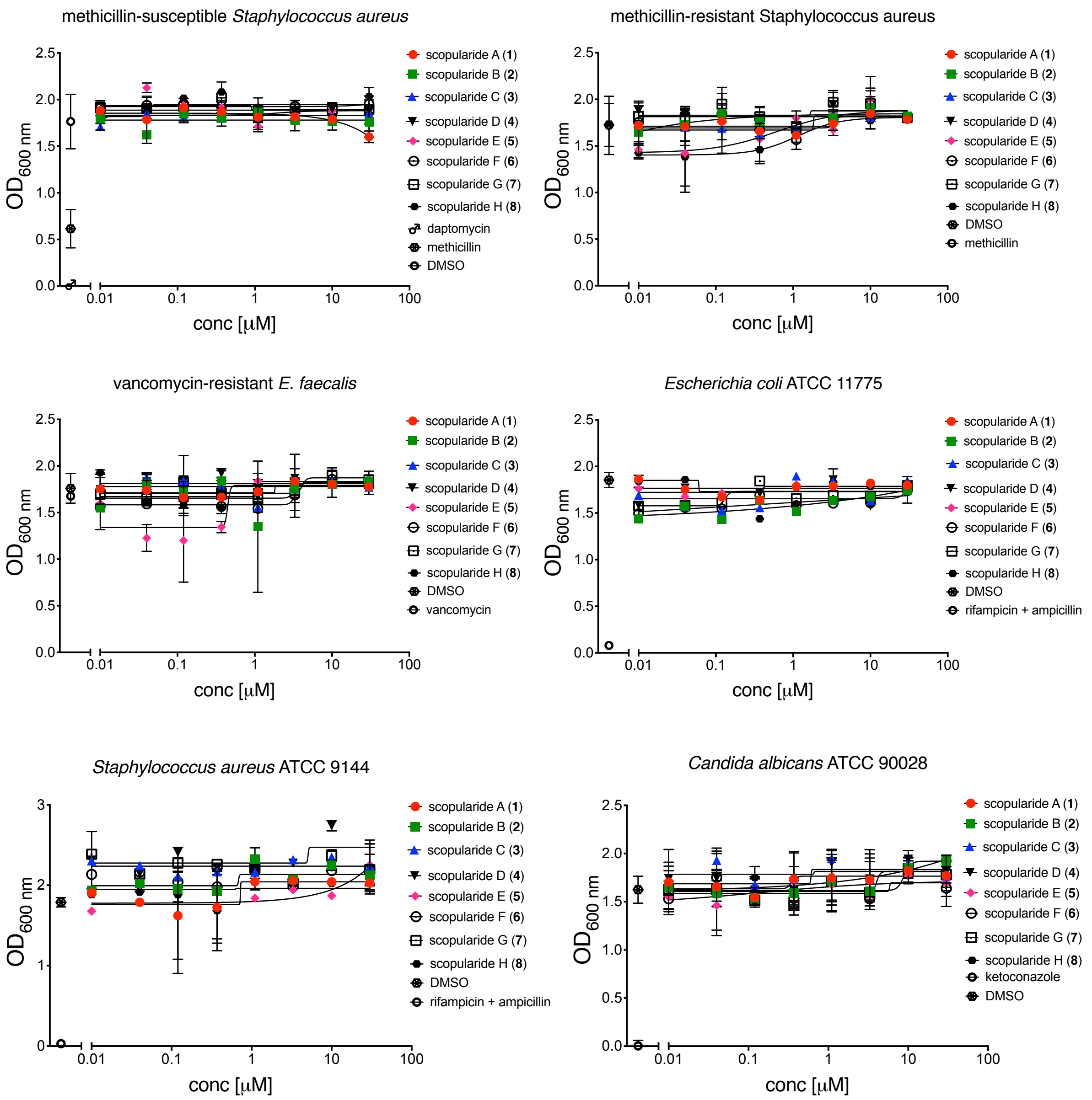

Figure S57. Growth inhibitory activity of scopularides A-H (**1**-**8**)

7.2 Cytotoxicity assay

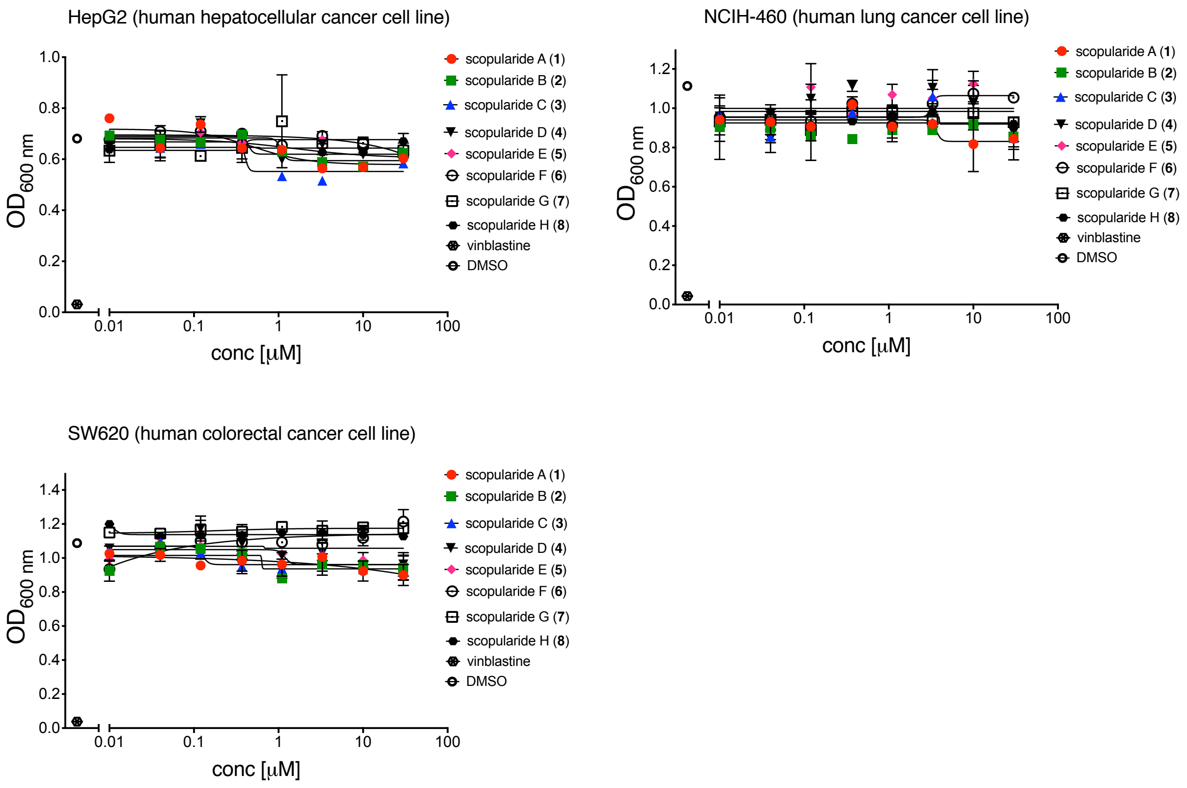

Figure S58. Cytotoxicity assay of the scopularides A-H (**1-8**) against (a) HepG2 (human hepatocellular cancer cell line), (b) NCI-H460 (human lung cancer cell line) and (c) SW620 (human colorectal cancer cell line)

8 References

1. Yang, Z.; Goldman, N.; Friday, A. Comparison of models for nucleotide substitution used in maximum-likelihood phylogenetic estimation. *Mol. Biol. Evol.* **1994**, *11*, 316–324.

2. Edgar, R. C. MUSCLE: a multiple sequence alignment method with reduced time and space complexity. *BMC Bioinformatics.* **2004**, *5*, 113.

3. Okonechnikov, K.; Golosova, O.; Fursov, M.; UGENE team Unipro UGENE: a unified bioinformatics toolkit. *Bioinformatics.* **2012**, *28*, 1166–1167.

4. Sheldrick, G. M. A short history of SHELX. *Acta Crystallogr., A, Found. Crystallogr.* **2008**, *64*, 112–122.

5. Farrugia, L. J. ORTEP-3 for Windows - a version of ORTEP-III with a Graphical User Interface (GUI). *J. Appl. Crystallogr.* **1997**, *30*, 565–565.

6. Farrugia, L. J. WinGX suite for small-molecule single-crystal crystallography. *J. Appl. Crystallogr.* **1999**, *32*, 837–838.

7. Yu, Z.; Lang, G.; Kajahn, I.; Schmaljohann, R.; Imhoff, J. F. Scopularides A and B, cyclodepsipeptides from a marine sponge-derived fungus, *Scopulariopsis brevicaulis*. *J. Nat. Prod.* **2008**, *71*, 1052–1054.

8. Asolkar, R. N.; Freel, K. C.; Jensen, P. R.; Fenical, W.; Kondratyuk, T. P.; Park, E.-J.; Pezzuto, J. M. Arenamides A-C, cytotoxic NFkappaB inhibitors from the marine actinomycete *Salinispora arenicola*. *J. Nat. Prod.* **2009**, *72*, 396–402.

9. Vijayasarathy, S.; Prasad, P.; Fremlin, L. J.; Ratnayake, R.; Salim, A. A.; Khalil, Z.; Capon, R. J. C_3 and 2D C_3 Marfey's Methods for Amino Acid Analysis in Natural Products. *J. Nat. Prod.* **2016**, *79*, 421–427.
